# Theoretical Study of the Mechanism of the Formation of Azomethine Ylide from Isatine and Sarcosine and Its Reactivity in 1,3-Dipolar Cycloaddition Reaction with 7-Oxabenzonorbornadiene †

**DOI:** 10.3390/ijms25126524

**Published:** 2024-06-13

**Authors:** Ivana Antol, Petar Štrbac, Yasujiro Murata, Davor Margetić

**Affiliations:** 1 Laboratory for Physical Organic Chemistry, Division of Organic Chemistry and Biochemistry, Ruđer Bošković Institute, Bijenička Cesta 54, HR-10002 Zagreb, Croatia; 2 Structural Organic Chemistry Laboratory, Division of Synthetic Chemistry, Institute for Chemical Research, Kyoto University, Gokasho, Uji, Kyoto 611-0011, Japan

**Keywords:** 1,3-dipolar cycloadditions, DFT calculations, isatine, azomethine ylide, reaction mechanism

## Abstract

The reaction mechanism of tthe formation of azomethine ylides from isatins and sarcosine is addressed in the literature in a general manner. This computational study aims to explore the mechanistic steps for this reaction in detail and to assess the reactivity of formed ylide in a 1,3-dipolar cycloaddition reaction with 7-oxabenzonorbornadiene. For this purpose, density functional theory (DFT) calculations at the M06-2X(SMD,EtOH)/6-31G(d,p) level were employed. The results indicate that CO_2_ elimination is the rate-determining step, the activation barrier for 1,3-dipolar cycloaddition is lower, and the formed ylide will readily react with dipolarophiles. The substitution of isatine with electron-withdrawal groups slightly decreases the activation barrier for ylide formation.

## 1. Introduction

**Figure 1:**
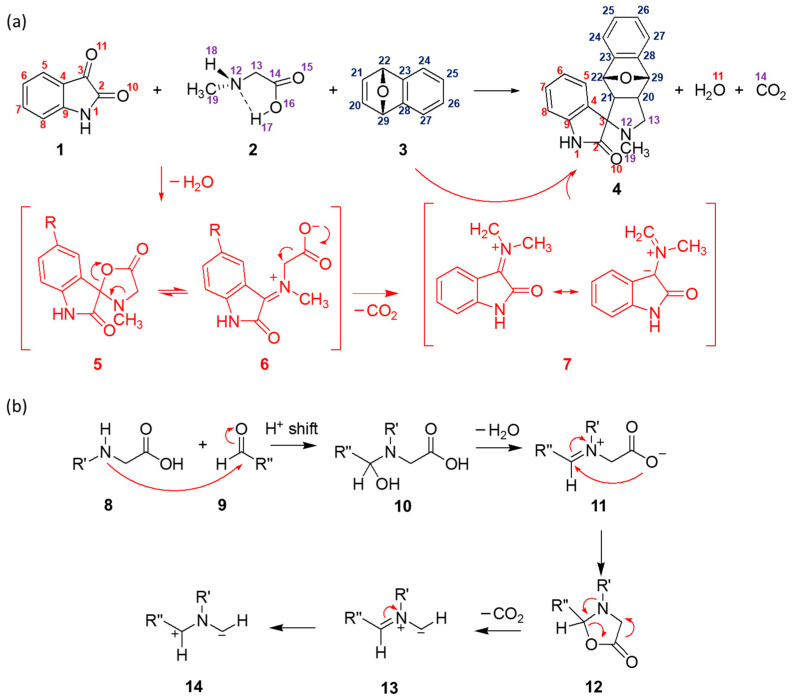
(**a**) Model reaction (black) studied herein with reaction mechanism (red) from Ref. [4]. (**b**) Mechanism of formation of azomethine ylide from modified glycines and aldehydes from Ref. [8].

The synthetic utility of azomethine ylides in the formation of heterocyclic molecules via their involvement in 1,3-dipolar cycloaddition with olefinic bonds has been well recognized and explored in numerous reactions [1,2]. An important strategy for the synthesis of complex structures possessing pyrrolidine rings is via 1,3-dipolar cycloadditions of azomethine ylides that are generated in situ from carbonyl compounds and appropriate amino acids. This approach has found wide applications, such as functionalization of fullerenes through the Prato reaction, in which aldehydes and sarcosine (*N*-methylglycine, **2**) are employed [3]. Similarly, isatine (**1**) and sarcosine (**2**) have been used as precursors for the in situ generation of azomethine ylide reagent for subsequent 1,3-dipolar cycloaddition with the C=C bond of 7-oxabenzonorbornadiene (**3**) and its derivatives [4]. The reaction is shown in black color in Figure 1a. It is important to note that the reaction benefits from polar (alcoholic) solvents such as ethanol and methanol, while lower yields can be achieved with less polar solvents.

While the syntheses of pyrrolidines utilizing the formation of azomethine ylides from carbonyl compounds and sarcosine have been described, mechanistic studies are scarce. As far as we are aware, published computational studies are focused on the regio- and stereoselectivities of 1,3-dipolar cycloadditions [5,6], while studies which explore the full reaction mechanism and, particularly, the formation of azomethine ylides are rather limited [7].

In the literature, the reaction mechanism is, in general, just briefly outlined. For example, the proposed reaction mechanism (red color, Figure 1a) involves the formation of cyclic intermediate **5** via dehydration in the first step, followed by opening of the 5-membered ring and decarboxylation. Once formed, azomethine ylide **7** reacts with 7-oxabenzonorbornadiene **3**, resulting in the formation of adduct **4**. Also, a more detailed mechanism of the formation of azomethine ylides from modified glycine and aldehydes (containing the desired R″ group) was proposed by Henderson et al. [8] (Figure 1b). The latter mechanism could be utilized for the description of the formation of azomethine ylide from isatine and sarcosine as well. This is exactly the main goal of this study. We reinvestigate the reaction mechanism of the model reaction in detail using DFT calculations. The effect of functionalization of isatine in position C6 (the numbering scheme is given in Figure 1a) is studied as well.

## 2. Results and Discussion

**Figure 2:**
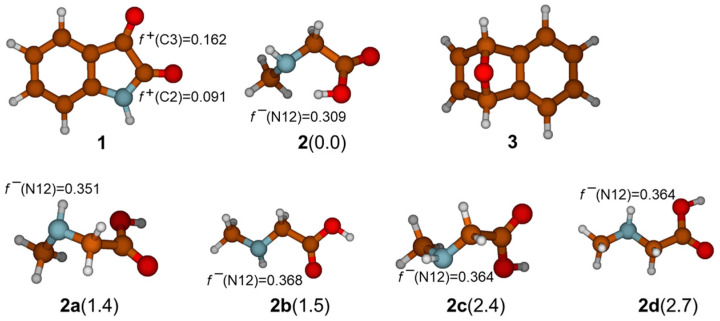
Optimized structures of reactants by M06-2X/6-31G(d,p) methods using SMD(EtOH) solvation model (relative energies of different conformers of sarcosine **2** are given in kcal mol^−1^ in parentheses). The condensed-to-atoms Fukui functions (*f*) for selected atoms are given as well.

Mechanistic investigations of the reaction were started by geometry optimization of reactants: isatine **1**, sarcosine **2**, and 7-oxabenzonorbornadiene **3** (Figure 2). Contrary to **1** and **3**, sarcosine is conformationally flexible, and several minima on the potential energy surface were found (**2**, **2a**–**2d**, Figure 2). The most stable structure (global minimum **2**) had an intramolecular hydrogen bond between the amino nitrogen atom (N12) and the hydrogen (H17) atom from the carboxylic group.

**Figure 3:**
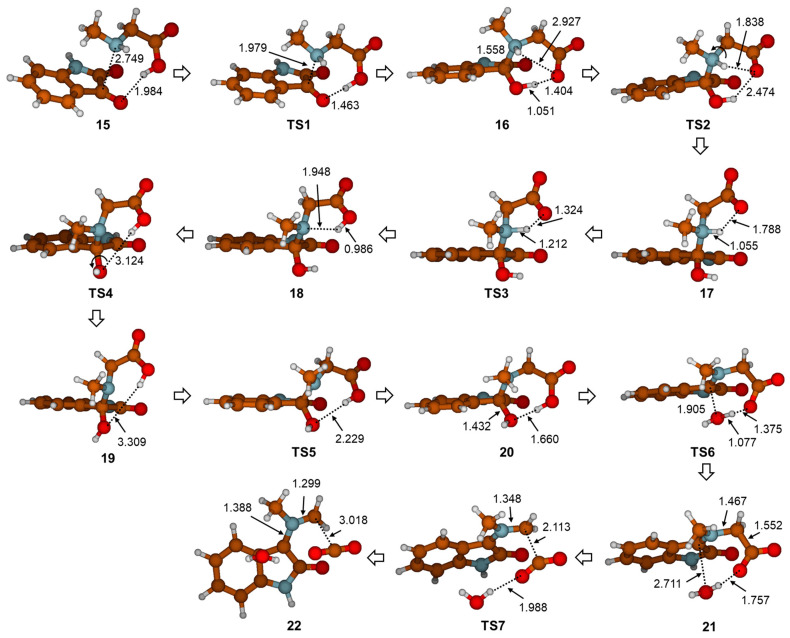
Stationary points along the reaction path describing the generation of azomethine ylide (**22**) from isatine **1** optimized with the M06-2X(SMD,EtOH)/6-31G(d,p) method. Selected geometry parameters are given in Å.

Although other stretched-out structures **2a**–**2d** (found as local minima on the potential energy surface of **2**) are a few kcal mol^−1^ less stable, they have better nucleophilic properties. The nucleophilic power of the N12 atom in these structures was evaluated from calculated values of the condensed-to-atoms Fukui functions (*f*) (see Figure 2). Parr and Yang proposed that a larger Fukui function value at an atom favors its reactivity [9,10]. Therefore, it is assumed that a stretched molecule is better for the formation of the van der Waals complex when isatine and sarcosine are in favorable orientation for the first reaction step. Indeed, in structure **15** (Figure 3), a lone pair from N12 is in electrostatic interaction with a positively charged C3 atom from the carbonyl group on isatine, and also, a new H bond between H17 and O11 is formed. This orientation allows for the simultaneous closing of the bond between C3 and N12 atoms and the proton transfer of H17 from O16 to O11. In the **TS1** structure, the C3-N12 bond length is 1.979 Å, and in intermediate zwitterion structure **16**, it assumes a value of 1.558 Å. At the same time, the distance between O11 and H17 decreases from 1.463 in **TS1** to 1.051 Å in **16**, confirming the simultaneous occurrence of these two geometrical changes (Figure 3 and Table 1).

**Figure 4:**
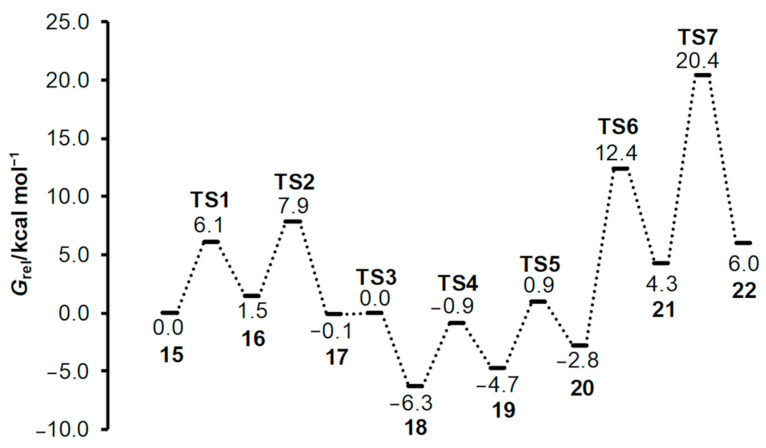
Energy profile along reaction path describing the generation of azomethine ylide (**22**). Gibbs free energies at M06-2X(SMD,EtOH)/6-31G(d,p) level of theory are given relative to structure **15**. All structures are shown in Figure 3.

Inspection of the energy profile along the reaction path leading to the generation of azomethine ylide (**22**), as shown in Figure 4, reveals that the energy barrier for the primary nucleophilic addition of sarcosine to **1** is only 6.1 kcal mol^−1^. The relative Gibbs free energy of addition product **16** is 1.5 kcal mol^−1^. The corresponding addition of **1** to the other isatine carbonyl group (C2=O10) was also considered. Although the C2 atom had a larger Hirsfeld positive partial atomic charge (0.277|e^−^|) than the C3 atom (0.196|e^−^|), indicating higher electrostatic interactions with the N12 reactive center, the calculated values of Fukui functions for the C2 atom were smaller (see Figure 2) by 0.071, meaning that the electrons received in the nucleophilic addition of sarcosine to the C2=O carbonyl group were poorly accommodated. Indeed, it was energetically more demanding, with an energy barrier of 10.1 kcal mol^−1^, which indicates high regioselectivity of the first mechanistic step. This is consistent with the high regioselectivity of this reaction, which was experimentally observed earlier [4], and therefore, this direction of the potential energy surface will not be considered further.

Charge separation within intermediate **16**, proven by the calculated high total dipole moment (17.95 Debye), could be minimized by rotation around the C13-N12 bond via **TS2** to **17**, where the carboxyl group was oriented toward H18 from the NH bond and energetically favorable second proton transfer via **TS3** to structure **18**. The dipole moment in structure **18** drastically decreased with respect to **16**, to 8.2 Debye. Structure **18** was the most stable intermediate (7.8 and 6.3 kcal mol^−1^ more stable than zwitterion **16** and the starting van der Waals complex **15**, respectively) (Figure 4). Before the dehydration step, two minor intramolecular conformational changes should occur. One is OH group rotation around the C3-O11 bond (see **TS4**, Figure 3 and Table 1) to achieve better orientation of the oxygen lone pair toward H18 from the COOH group in structure **19**. The second one is pyramidalization on the N12 (see **TS5**, Figure 3 and Table 1). In the resulting structure **20**, the distance between O11 and H18 decreased to 1.660 Å. This interaction facilitated the dissociation of the C3-O11 bond via **TS6** because the H18 proton transfer to the OH group formed a leaving water molecule and initiated dehydration. The C-O distance in the **TS6** was 1.905 Å, and is shown to stretch further to 2.711 Å in structure **21**. The intermediate **21** was zwitterionic, with a total dipole moment of 19.52 Debye. The last step in the reaction path for azomethine ylide was decarboxylation. It can be described with **TS7**. The most important geometrical changes along the decarboxylation path were C13-C14, N12-C13 distances, and O16-C14-O15 valence angle. While the C13-C14 bond lengths increased in the sequence 1.552, 2.113, and 3.018 Å, the N-C13 lengths decreased in the order 1.467, 1.348, and 1.299 Å in structures **21**, **TS7,** and **22**, respectively (Figure 3). The angle O16-C14-O15 gradually expanded due to the formation of linear atom arrangement in the CO_2_ molecule. Energy barriers for H_2_O and CO_2_ cleavages were 15.2 and 16.1 kcal mol^−1^ in structures **20** and **21**, respectively. Finally, based on the relative free energies calculated for all transition structures along the reaction pathway (Figure 4), one can conclude that the decarboxylation step was the key step that determined the energetics of azomethine ylide formation, starting with complex **15**. The overall process was endergonic, since *G*_rel_(**22**) = 6.0 kcal mol^−1^. 

**Figure 5:**
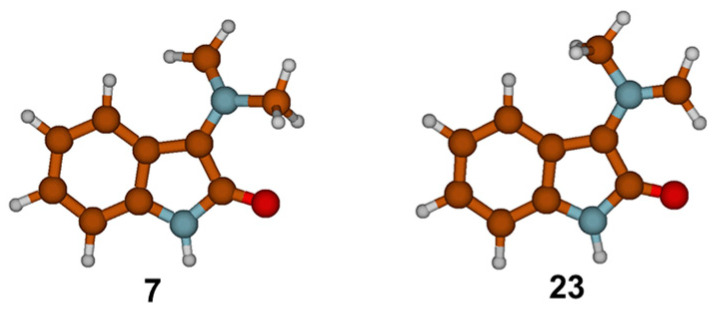
Optimized structures of two possible isomers of azomethine ylide by M06-2X/6-31G(d,p) methods using SMD(EtOH) solvation model.

**Figure 6:**
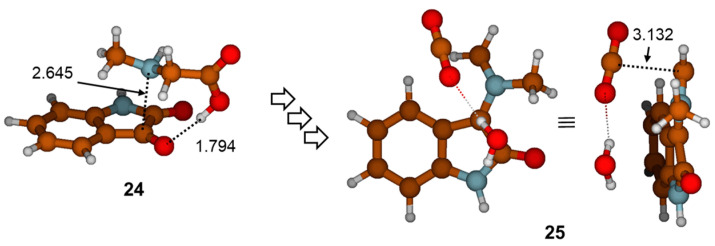
Energy minima **24** and **25** optimized with M06-2X(SMD,EtOH)/6-31G(d,p) method. Selected geometry parameters are given in Å. All details on the reaction path between **24** and **25** are given in Figures S1 and S2 in Supplementary Materials.

Azomethine ylide within structure **22**, generated through the reaction path described in Figure 3 and Figure 4, corresponds to azomethine ylide structure **23** (see Figure 5). The formation of the other isomer **7** (less stable by 0.8 kcal mol^−1^), which is related to **23** via *E/Z* isomerization, started with van der Waals complex **24** and ended with structure **25** (see Figure 6). The details of the reaction path between **24** and **25** are given in Figures S1 and S2 in the Supplementary Materials. The reaction path, as found before, involved nucleophilic addition to the carbonyl bond, several intramolecular proton transfers, and conformational changes, followed by dehydration and decarboxylation. The values of the relative free energies of significant TS structures are also comparable. Hence, it appears that, mechanistically, there is no difference between the formation of azomethine ylides **7** and **23**. Thermodynamically, there is a slight difference: starting van der Waals complex **24** is 0.8 kcal mol^−1^ less stable than **15**.

**Figure 7:**
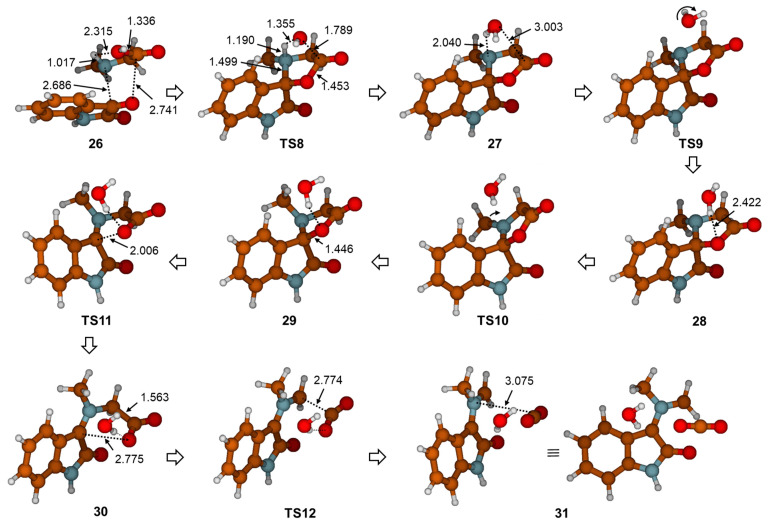
Stationary points along reaction path describing the generation of azomethine ylide (**23** = **31**) optimized with M06-2X(SMD,EtOH)/6-31G(d,p) method. Selected geometry parameters are given in Å.

**Figure 8:**
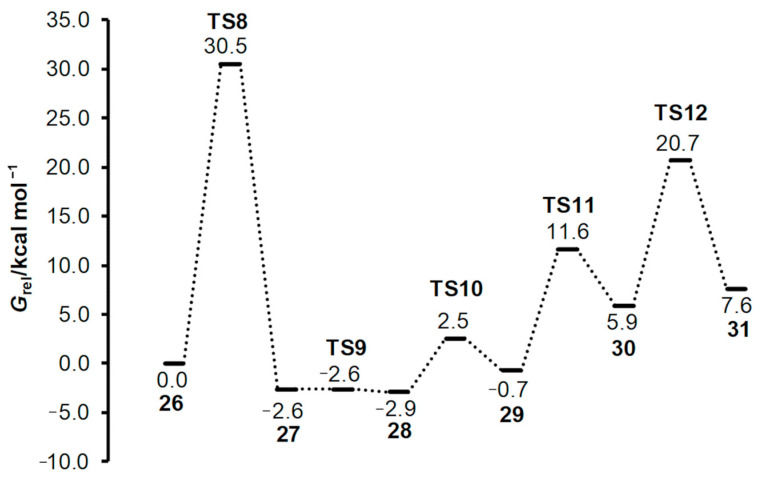
Energy profile along reaction path describing the generation of azomethine ylide (**23** = **31**). Gibbs free energies at M06-2X(SMD,EtOH)/6-31G(d,p) level of theory are given relative to the structure **26**. All structures are shown in Figure 7.

The mechanism for the formation of azomethine ylide described herein differs from the mechanism proposed by Parthasarathy et al. [4]. To test whether it is possible to form the cyclic intermediate **5** via dehydration in the first step of the reaction, as assumed, we calculated the **TS8** structure (Figure 7). The intrinsic reaction coordinate (IRC) following in a reversed direction gave the van der Waals complex **26**. On the other side, IRC calculation in the forward direction gave the structure **27**, which corresponds to the water-coordinated intermediate **5**. This means that the **TS8** is, indeed, the transition structure for synchronous addition and dehydration. Further stationary points and the energy profile for generating azomethine ylide after the formation of intermediate **27** are given in Figure 7 and Figure 8, respectively. The processes that follow were relocation of water molecule on very flat part of the potential energy surface via **TS9**; pyramidalization of the N12 atom via **TS10**; ring opening by means of C3-O11 bond dissociation via **TS11**; and, lastly, decarboxylation via **TS12**. We note in passing that, in the complex intermediate structure **31** produced in the later step, azomethine ylide assumed the conformation equivalent to **23** (the same configuration was already found in the outcome of a previously described reaction path in Figure 3 and Figure 4, structure **22**). The slight difference in electronic energies between the two complexes **31** and **22** was due to different H_2_O position. The data presented in Figure 8 illustrate that the **TS8** was the highest-energy transition structure (*G*_rel_(**TS8**) = 30.5 kcal mol^−1^ above complex **26**), and activation of this reaction path (associated with the **TS8**) is very improbable. 

The next point of great interest to us is the effect of substitution in the C6 position of isatine **1** on the reactivity of ylide formation for the planned application of isatins as cycloaddition delivery reagents for guanidine functionality [11]. The influence of substituents at C6 was assessed by examination of the decarboxylation reaction—the rate-determining step defined by **TS7**. The relative energies for **TS7** and **22** with the amino (**TS7a**, **22a**), nitro (**TS7b**, **22b**), and guanidino (**TS7c**, **22c**) groups, with respect to the corresponding **21a**, **21b**, and **21c** structures, respectively, are given in Table 2. Due to the high basicity of guanidines [12], it was likely that the guanidine functional group would be protonated. Therefore, the effect of protonation of the guanidino group in structures **TS7d**, **21d,** and **22d** was studied as well. 

During the cleavage of CO_2_ from **21**, a substantial redistribution of atomic charges took place. For instance, the positive partial charge on the C1 atom increased by 0.12 and 0.20 |e^−^| in **TS7** and structure **22**, respectively (see **Δ***q*(C1) in Table 2). This decrease was not affected by substitution, probably because the substituents attached on the C6 were far from the reaction center. Subsequently, the influence of different functional groups on the reactivity of decarboxylation was low. The obtained activation energies were in the 12.0–16.4 kcal mol^−1^ range. The largest effects were noticed for nitro (-NO_2_) and protonated guanidino (-NHC(NH_2_)_2_^+^) substituents. In those cases, the calculated energy barriers were decreased by 4.1 and 3.0 kcal mol^−1^, respectively, showing that an electron-withdrawing group favorably affects the reactivity.

**Figure 9:**
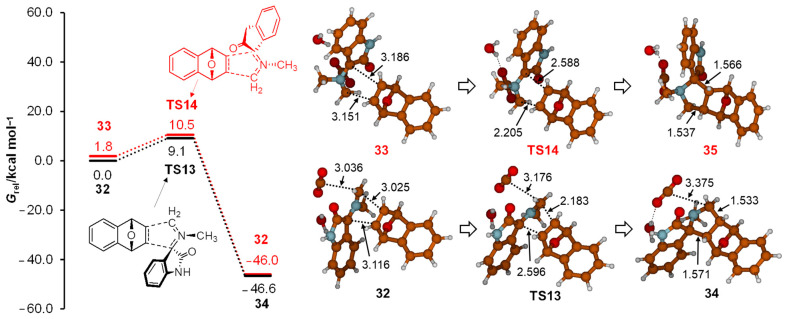
Energy profile and structures of stationary points along the reaction path describing 1,3-dipolar cycloaddition of 7-oxabenzonorbornadiene with azomethine ylide. Gibbs free energies at M06-2X(SMD,EtOH)/6-31G(d,p) level of theory are given relative to structure **32**. Selected geometric parameters are given in Å.

Once the mechanism of azomethine ylide intermediate formation was established, we next turned to study its 1,3-dipolar cycloaddition with the 7-oxabenzonorbornadiene **3**. It is well known that due to the slight *endo*-pyramidalization of the C20-C21 double bond in **3** [13], cycloadditions preferentially take place in the *exo* fashion [14]. Therefore, the azomethine ylide **22** was oriented toward **3** on the *exo* side for the optimization of complex structures **32** and **33** (Figure 9). In both structures, the C20-C21 bond was more than 3 Å distant from the ylide plane. Corresponding transition structures for cycloaddition **TS13** and **TS14** were located at 9.1 and 10.5 kcal mol^−1^ above **32** minimum. Further shortening of these distances led to the formation of new carbon–carbon bonds in the final adduct **34**. The minima **34** was the most stable structure *G*_rel_(**34**) = −46.6 kcal mol^−1^. The calculated stereospecificity of the cycloaddition was in full agreement with the experimentally observed structures of cycloadducts **34** [4] and the formation of a single product. Employing the energies of stationary points along the reaction path for 1,3-dipolar cycloaddition of 7-oxabenzonorbornadiene **3** with azomethine ylide, it can be concluded that the 1,3-dipolar cycloaddition reaction of azomethine ylide **22** and 7-oxabenzonorbornadiene **3** is a very exergonal step, and also, it is less energy-demanding than the decarboxylation step for the generation of **22**. This conclusion is additionally corroborated by calculations where CO_2_ and H_2_O molecules were deleted from the first coordination sphere, resulting in a slightly smaller energy barrier for cycloaddition of 8.4 kcal mol^−1^.

**Figure 10:**
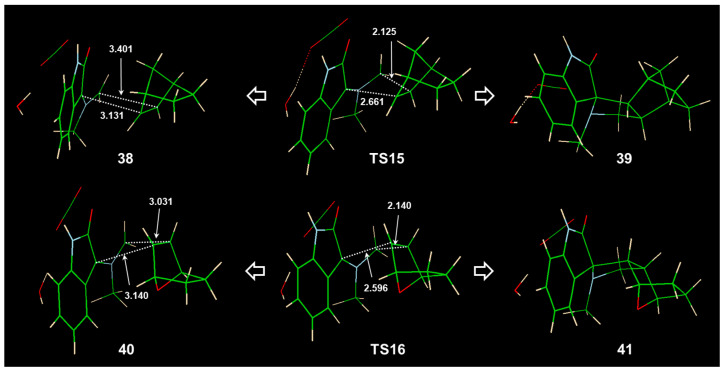
TS structures for 1,3-dipolar cycloaddition of azomethine ylide **22** with norbornene **36** and 7-oxanorbornene **37** (**TS15** and **TS16**, respectively), optimized with the M06-2X(SMD,EtOH)/6-31G(d,p) method. Selected geometry parameters are given in Å.

The thermodynamic stability of structures **32** and **TS13** with respect to **33** and **TS14**, respectively, can be rationalized by electrostatic interactions of the oxa bridge (electronegative O) with the positive N12 atom from ylide. This interaction is favorable; it influences **32** and **TS13** structures, where the oxa bridge and NCH_3_ groups are oriented on the same side in space. The opposite holds for **33** and **TS14**, since the oxa bridge and NCH_3_ groups are oriented on different sides in space, decreasing their interaction through space. To better understand these interactions, additional calculations involving norbornene (**36**) and 7-oxanorbornene (**37**) dienophiles were conducted (see Figure 10).

In contrast to the oxa bridge, the local charges on the CH_2_ bridge in norbornene were positive, and its interaction with N13 was unfavorable. Indeed, it was found that, in the reaction of norbornene **36** with the azomethine ylide **22** in transition structure **TS15** (Figure 10), the CH_2_ bridge oriented on the opposite side of NCH_3_ from ylide was in a preferred orientation. The reaction was still very exergonic, since product **39** was 51.7 and 39.4 kcal mol^−1^, respectively, more stable than the **TS15** and structure **38** (van der Waals complex between **22** and **36**). It should be noted that **TS15** as 2.4 kcal mol^−1^ less stable than **TS7** (TS for decarboxylation); therefore, in the case of norbornene, cycloaddition is a rate-determining step on the reaction path. The high energy of **TS15**, thus, follows norbornene’s experimental inertness [4]. To quantify the oxa bridge’s importance for the reaction, we compared the energy of **TS15** with TS for cycloaddition with 7-oxanorbornene **TS16** (Figure 10). Substitution of CH_2_ with O stabilized the TS by 3.8 kcal mol^−1^ and was beneficial for the reaction.

In this study, the solvent effect was considered by the implicit SMD solvation model. However, the solvent molecule (EtOH) was able to directly form hydrogen bonds with some transition structures and intermediates, particularly zwitterionic structures with large dipole moments, such as **16** or **21**. It could also facilitate intramolecular proton transfers to some extent. However, we should point out that, for the key reaction steps (decarboxylation and cycloaddition), extensive additional effects upon explicit EtOH inclusion in the reactive site are not expected because, in that case, the microsolvation is already taken into account via the present H_2_O molecule, which possesses better solvation properties than EtOH.

## 3. Materials and Methods

The M06-2X DFT functional developed by Truhlar et al. [15] was used to optimize the structures of reactants, transition states, intermediates, and products. The XYZ coordinates of all structures are given in Table S1 in Supplementary Materials. The selected method has very good performance in applications involving main-group thermochemistry, kinetics, electronic excitation energies, and noncovalent interactions [9]. It is designed to include dispersion effects at an electronic level and to work quite well for weakly bound systems at their equilibrium distances [16,17]. The M06-2X density functional is used in conjunction with the Pople’s double-ζ basis set 6-31G(d,p) [18,19]. The solvent effects during the optimizations were included using the SMD method [20], and ethanol was used as a solvent with default parameters as defined in the Gaussian16 [21] software package. Ethanol was selected because, in non-alcoholic solvents, the products of the reaction were generated in lower yields [4]. No symmetry constraints were imposed. Vibrational analysis was performed, and all structures were characterized either as minima without imaginary frequencies or as transition state structures with one imaginary frequency. The total energy of each stationary point on the surface of the potential energy was corrected by unscaled ZPV energy. The association of products with reactants via transition structures was confirmed by intrinsic reaction coordinate (IRC) calculations. Partial atomic charges were extracted from the Hirshfeld population analysis [22]. Condensed Fukui functions [23] on the selected reacting atoms in reactants were calculated as the difference between Hirshfeld atomic charges of neutral and radical anion (for atoms as electrophiles) or radical cation (for atoms as a nucleophile) species at the geometries of the neutral minima. In IRC calculations, vibrational analyses, and in single-point calculations for population analysis, the M06-2X functional was used as well. The Gaussian16 [21] software package was used to perform quantum-mechanical calculations, and the initial structures were generated using the Molden package [24].

## 4. Conclusions

The DFT theoretical investigation of the formation of azomethine ylide from isatine and sarcosine and its reactivity in a 1,3-dipolar cycloaddition reaction with 7-oxabenzonorbornadiene enabled detailed elucidation of the reaction mechanism. It is shown in this study that the reaction started with the simultaneous nucleophilic addition of sarcosine to carbonyl group of isatine (C3=O11) and proton transfer from O16 to O11. Further, several conformational changes (rotations and pyramidalization) and proton transfers were followed by dehydration and decarboxylation, which led to the formation of azomethine ylide intermediate. We showed that proposed cyclic intermediates in the literature (structures **5** or **12**) were not included in the mechanism. The CO_2_ cleavage was recognized as the rate-determining step, which could be expedited by substitution in position C6 of isatine with electron-withdrawing groups such as -NO_2_ and protonated guanidine. Finally, the last reaction step, where the 1,3-dipolar cycloaddition of azomethine ylide and 7-oxabenzonorbornadiene produced a stabile fused spiro pyrrolidine-oxindole product, was energetically advantageous due to beneficial interactions of the oxa bridge with ylide N12 atom. 

## Figures and Tables

**Table 1:** List of transition structures and their imaginary frequencies.

Transition	Structure	Freq/cm^−1^	Description
TS1	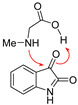	−179.8	Nucleophilic addition + simultaneous proton shift
TS2	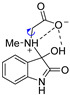	−69.9	Rotation around the C13-N12 bond
TS3	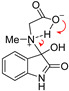	−931.0	Proton transfer
TS4	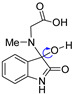	−403.0	Rotation of OH group—rotation around the C3-O11 bond
TS5	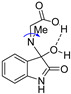	−85.2	N12 pyramidalization
TS6	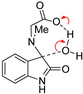	−344.8	H_2_O cleavage
TS7	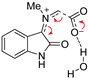	−575.2	Decarboxylation

**Table 2:** Relative Gibbs free energies in kcal mol^−1^ of transition structures (**TS7**–**TS7d**) for decarboxylation step in the formation of key intermediates **22**–**22d**. Relative energies with respect to reactants are given as well.

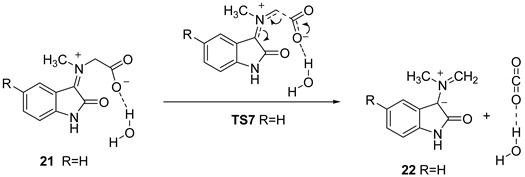						
	TS7			22		
	*G*_rel_ ^1^	*G*_rel_ ^2^	Δ*q*(C1)	*G*_rel_ ^1^	*G*_rel_ ^2^	Δ*q*(C1)
R=H	16.1	26.0	−0.12	1.7	11.6	−0.20
R=NH_2_	16.4	26.0	−0.12	2.9	12.4	−0.21
R=NO_2_	12.0	23.0	−0.12	−4.1	6.8	−0.21
R=GU	14.8	24.3	−0.11	0.8	10.3	−0.20
R=GUH^+^	13.1	21.2	−0.12	−2.2	5.9	−0.21

^1^ with respect to **21**; ^2^ with respect to reactants.

## Data Availability

Data is contained within the article and Supplementary Materials.

## Supplementary Materials

The following supporting information can be downloaded at: https://www.mdpi.com/article/10.3390/ijms25126524/s1.

## References

[1] Dubey S., Pal A., Roy S., Sasmal S., Tamrakar A., Jana R., Das T. (2023). Recent advances in the (3+2) cycloaddition of azomethine ylide. New J. Chem..

[2] Warrener R.N., Margetić D., Butler D.N., Sun G. (2001). Neighbouring Group Participation in *N*-Methoxymethyl 7-Azanorbornanes 1: The Synthesis of *N*,*N*′-Methano-bridged Diazasesquinorbornanes, N^3^-[3]Polynorbornanes and CN^3^-[4]Polynorbornanes. Synlett.

[3] Maggini M., Scorrano G., Prato M. (1993). Addition of azomethine ylides to C60: Synthesis, characterization, and functionalization of fullerene pyrrolidines. J. Am. Chem. Soc..

[4] Kumaran S., Saritha R., Gurumurthy P., Parthasarathy K. (2020). Synthesis of Fused Spiropyrrolidine Oxindoles Through 1,3-Dipolar Cycloaddition of Azomethine Ylides Prepared from Isatins and α-Amino Acids with Heterobicyclic Alkenes. Eur. J. Org. Chem..

[5] Gugkaeva Z.T., Panova M.V., Smol’Yakov A.F., Medvedev M.G., Tsaloev A.T., Godovikov I.A., Maleev V.I., Larionov V.A. (2022). Asymmetric Metal-Templated Route to Amino Acids with 3-Spiropyrrolidine Oxindole Core via a 1,3-Dipolar Addition of Azomethine Ylides to a Chiral Dehydroalanine Ni(II) Complex. Adv. Synth. Catal..

[6] Filatov A.S., Pronina Y.A., Selivanov S.I., Shmakov S.V., Uspenski A.A., Boitsov V.M., Stepakov A.V. (2022). 11*H*-Benzo[4,5]imidazo[1,2-*a*]indol-11-one as a New Precursor of Azomethine Ylides: 1,3-Dipolar Cycloaddition Reactions with Cyclopropenes and Maleimides. Int. J. Mol. Sci..

[7] Wu P. (2015). Theoretical study of the mechanism generating azomethine ylide from formaldehyde and glycine. J. Struct. Chem..

[8] Servinis L., Gengenbach T.R., Huson M.G., Henderson L.C., Fox B.L. (2015). A Novel Approach to the Functionalisation of Pristine Carbon Fibre Using Azomethine 1,3-Dipolar Cycloaddition. Aust. J. Chem..

[9] Parr R.G., Yang W. (1984). Density functional approach to the frontier-electron theory of chemical reactivity. J. Am. Chem. Soc..

[10] Yang W., Parr R.G. (1985). Hardness, softness, and the fukui function in the electronic theory of metals and catalysis. Proc. Natl. Acad. Sci. USA.

[11] Briš A., Murata Y., Hashikawa Y., Margetić D. (2023). Utilization of *sym*-tetrazines as guanidine delivery cycloaddition reagents. An experimental and computational study. J. Mol. Struct..

[12] Margetić D., Trošelj P., Ishikawa T., Kumamoto T. (2010). Design of New Scaffolds for Increased Superbasicity of Bisguanidine Proton Sponges. Bull. Chem. Soc. Jpn..

[13] Margetić D., Williams R.V., Warrener R.N. (2003). Pyramidalized Olefins: A DFT Study of the Homosesquinorbornene and Sesquibicyclo[2.2.2]octene Nuclei. J. Org. Chem..

[14] Margetić D., Warrener R.N., Eckert-Maksić M., Antol I., Glasovac Z. (2003). A DFT study of pyramidalized alkenes: 7-oxasesquinorbornenes and 7,7’-dioxasesquinorbornenes. Theor. Chem. Acc..

[15] Zhao Y., Truhlar D.G. (2008). The M06 suite of density functionals for main group thermochemistry, thermochemical kinetics, noncovalent interactions, excited states, and transition elements: Two new functionals and systematic testing of four M06-class functionals and 12 other functionals. Theor. Chem. Acc..

[16] Bursch M., Mewes J., Hansen A., Grimme S. (2022). Best-Practice DFT Protocols for Basic Molecular Computational Chemistry. Angew. Chem. Int. Ed..

[17] Goerigk L., Kruse H., Grimme S. (2011). Benchmarking Density Functional Methods against the S66 and S66x8 Datasets for Non-Covalent Interactions. Chemphyschem.

[18] Marenich A.V., Cramer C.J., Truhlar D.G. (2009). Universal Solvation Model Based on Solute Electron Density and on a Continuum Model of the Solvent Defined by the Bulk Dielectric Constant and Atomic Surface Tensions. J. Phys. Chem. B.

[19] Hehre W.J., Ditchfield R., Pople J.A. (1972). Self—Consistent Molecular Orbital Methods. XII. Further Extensions of Gaussian—Type Basis Sets for Use in Molecular Orbital Studies of Organic Molecules. J. Chem. Phys..

[20] Hariharan P.C., Pople J.A. (1973). The influence of polarization functions on molecular orbital hydrogenation energies. Theor. Chem. Acc..

[21] Frisch M.J., Trucks G.W., Schlegel H.B., Scuseria G.E., Robb M.A., Cheeseman J.R., Scalmani G., Barone V., Mennucci B., Petersson G.A. (2019). Gaussian 16, Revision C.01.

[22] Marenich A.V., Jerome S.V., Cramer C.J., Truhlar D.G. (2012). Charge Model 5: An Extension of Hirshfeld Population Analysis for the Accurate Description of Molecular Interactions in Gaseous and Condensed Phases. J. Chem. Theory Comput..

[23] Yang W., Mortier W.J. (1986). The use of global and local molecular parameters for the analysis of the gas-phase basicity of amines. J. Am. Chem. Soc..

[24] Schaftenaar G., Noordik J.H. (2000). Molden: A pre- and post-processing program for molecular and electronic structures. J. Comput. Aided Mol. Des..

