# Supplementary material for: Theoretical Study of the Mechanism of the Formation of Azomethine Ylide from Isatine and Sarcosine and Its Reactivity in 1,3-Dipolar Cycloaddition Reaction with 7-Oxabenzonorbornadiene
Source: Int J Mol Sci. 2024 Jun 13;25(12):6524. doi: 10.3390/ijms25126524 (PMC11203580; doi:10.3390/ijms25126524)
Supplement: Supplementary file 1 [file ijms-25-06524-s001.zip › ijms-3051809-supplementary.pdf]

Supplementary Material

for

Theoretical Study of the Mechanism of the Formation of Azomethine Ylide from Isatine and Sarcosine and its Reactivity in 1,3-Dipolar Cycloaddition Reaction with 7-Oxabenzonorbornadiene

Ivana Antol[1,*], Petar Štrbac[1], Yasujiro Murata[2], and Davor Margetić [1,*]

[1] Laboratory for Physical Organic Chemistry, Division of Organic Chemistry and Biochemistry, Ruđer Bošković Institute, Bijenička cesta 54, HR-10002 Zagreb, Croatia
[2] Structural Organic Chemistry Laboratory, Division of Synthetic Chemistry, Institute for Chemical Research, Kyoto University, Gokasho, Uji, Kyoto 611-0011, Japan
* Correspondence: iantol@irb.hr (I.A.); margetid@irb.hr (D.M.); Tel.: +385-1-468-0197 (D.M.); Fax: +385-1-456-1008 (D.M.)

Content:

Figure S1. Stationary points along reaction path describing the generation of azomethine ylide **25** optimized with M06-2X(SMD,EtOH)/6-31G(d,p) method.
Figure S2. Energy profile along reaction path describing the generation of azomethine ylide (**25**). Gibbs free energies at M06-2X(SMD,EtOH)/6-31G(d,p) level of theory are given relative to **24**.
Table S1. Energies (atomic units), XYZ coordinates (Å) and Hirshfeld (CM5) charges on atoms of all structures optimized at M06-2X(SMD,EtOH)/6-31G(d,p) level of theory.

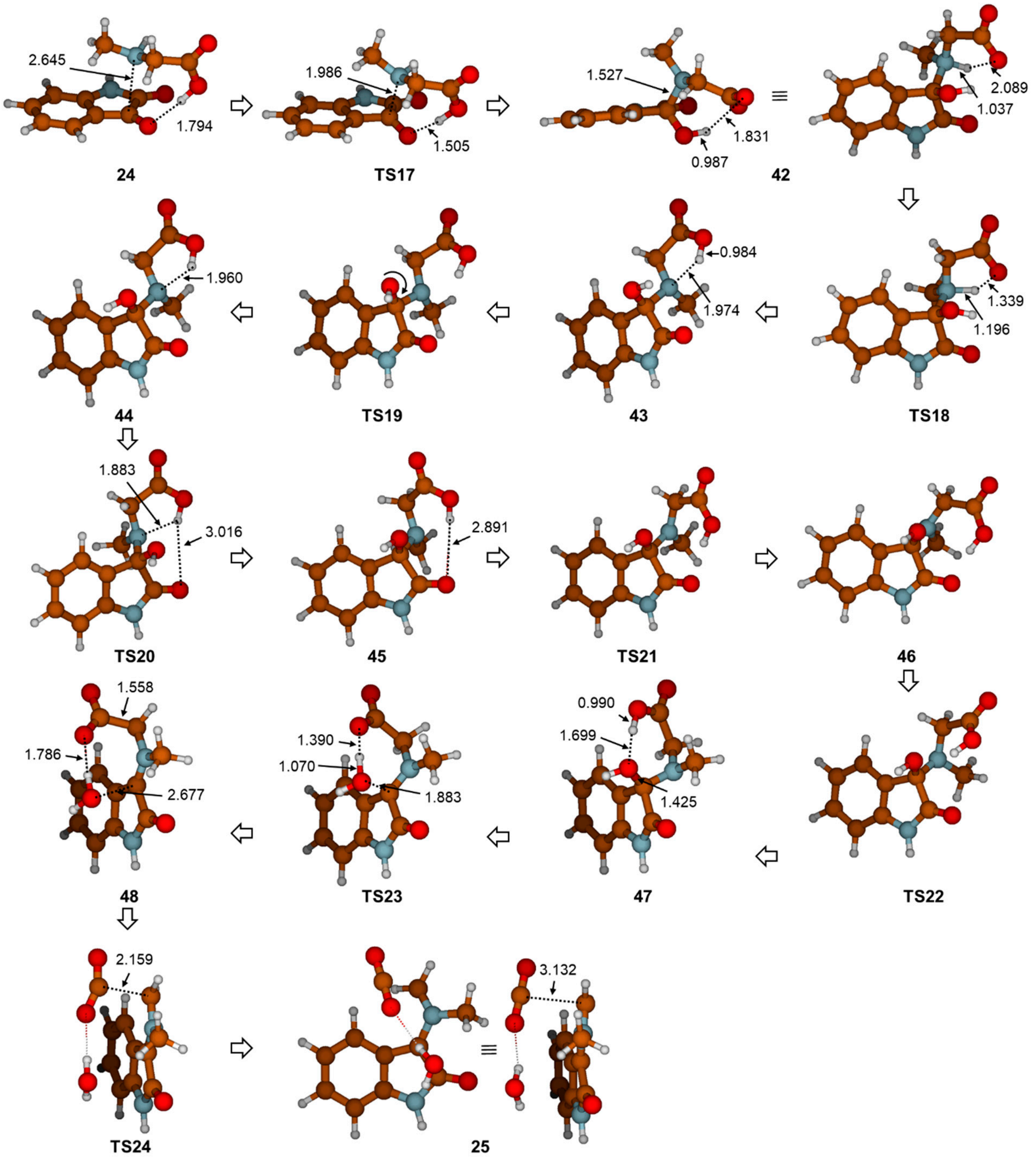

Figure S1. Stationary points along reaction path describing the generation of azomethine ylide **25** optimized with M06-2X/6-31G(d,p) SMD(EtOH) method. Selected geometry parameters are given in Å.

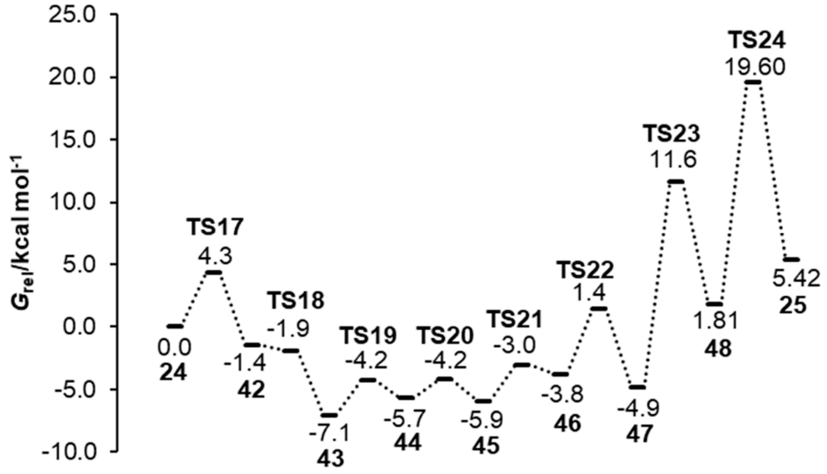

Figure S2. Energy profile along reaction path describing the generation of azomethine ylide (**25**). Gibbs free energies at M06-2X/6-31G(d,p) SMD(EtOH) level of theory are given relative to **24**. All structures are shown in Figure S1.

Table S1. Energies (atomic units), XYZ coordinates (Å) and Hirshfeld (CM5) charges on atoms of all structures optimized at M06-2X(SMD,EtOH) /6-31G(d,p) level of theory.

1

```
log file: r1.log
E(RM06-2X) =  -512.885085623     A.U.
Zero-point correction=                          0.310959
Thermal correction to Energy=                   0.331562
Thermal correction to Enthalpy=                 0.332506
Thermal correction to Gibbs Free Energy=        0.262067
Sum of electronic and zero-point Energies=          -991.203362
Sum of electronic and thermal Energies=             -991.182759
Sum of electronic and thermal Enthalpies=           -991.181815
Sum of electronic and thermal Free Energies=        -991.252255
```

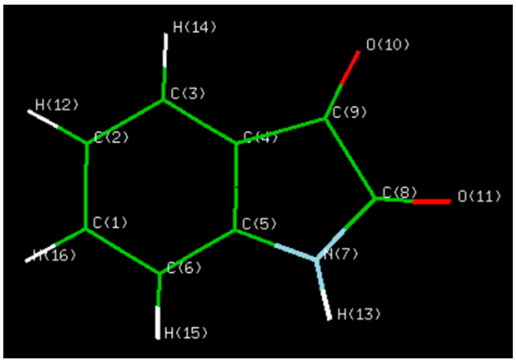

		x	y	z	q
1	C	-2.746226	-0.588107	0.000015	-0.069696
2	C	-2.635625	0.805577	-0.000010	-0.103645
3	C	-1.378232	1.405891	-0.000025	-0.064131
4	C	-0.259055	0.582862	-0.000007	-0.043692
5	C	-0.383430	-0.815612	0.000002	0.112216
6	C	-1.626013	-1.425228	0.000020	-0.096264
7	N	0.882847	-1.426952	-0.000056	-0.405902
8	C	1.884190	-0.504571	-0.000061	0.277430
9	C	1.174438	0.884500	0.000063	0.196236
10	O	1.773848	1.934425	0.000010	-0.307901
11	O	3.083312	-0.685781	0.000016	-0.362209
12	H	-3.531398	1.416492	-0.000010	0.116587
13	H	1.035811	-2.428698	0.000195	0.373559
14	H	-1.262200	2.485355	-0.000023	0.127726
15	H	-1.726482	-2.505010	0.000026	0.127153
16	H	-3.733209	-1.040487	0.000024	0.122542

2

```
log file: s1e.log
E(RM06-2X) =  -323.620432064     A.U.
Zero-point correction=                          0.310959
Thermal correction to Energy=                   0.331562
Thermal correction to Enthalpy=                 0.332506
Thermal correction to Gibbs Free Energy=        0.262067
Sum of electronic and zero-point Energies=          -991.203362
Sum of electronic and thermal Energies=             -991.182759
Sum of electronic and thermal Enthalpies=           -991.181815
Sum of electronic and thermal Free Energies=        -991.252255
```

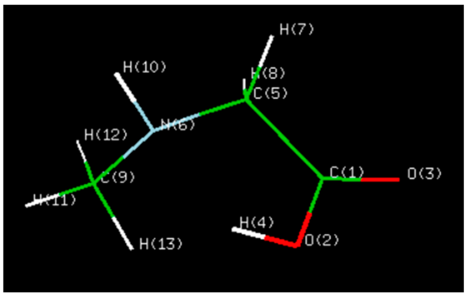

		x	y	z	q
1	C	1.077159	-0.028175	0.015897	0.259242
2	O	0.913212	1.266918	-0.235954	-0.398013
3	O	2.105470	-0.466062	0.484872	-0.404496
4	H	-0.002129	1.344534	-0.604037	0.340266
5	C	-0.114734	-0.891245	-0.371505	-0.044382
6	N	-1.286240	-0.053693	-0.570954	-0.505686
7	H	0.154215	-1.387062	-1.309696	0.137857
8	H	-0.236212	-1.667550	0.395143	0.117199
9	C	-2.041458	0.159788	0.662792	-0.127366
10	H	-1.886663	-0.457416	-1.282335	0.323219
11	H	-2.906361	0.793095	0.455480	0.106464
12	H	-2.387805	-0.777389	1.119292	0.092476
13	H	-1.406626	0.678581	1.388375	0.103223

2a

log file: s1c.log
E(RM06-2X) = -323.617520541 A.U.
Zero-point correction= 0.310959
Thermal correction to Energy= 0.331562
Thermal correction to Enthalpy= 0.332506
Thermal correction to Gibbs Free Energy= 0.262067
Sum of electronic and zero-point Energies= -991.203362
Sum of electronic and thermal Energies= -991.182759
Sum of electronic and thermal Enthalpies= -991.181815
Sum of electronic and thermal Free Energies= -991.252255

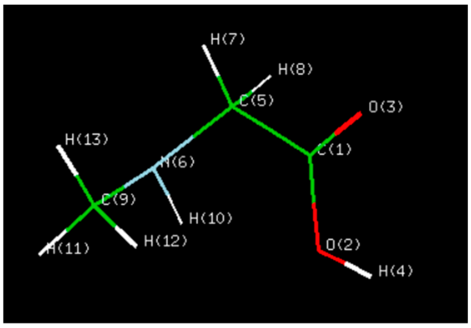

		x	y	z	q
1	C	-1.002156	-0.074049	0.003997	0.268675
2	O	-0.871990	1.195957	-0.392836	-0.372039
3	O	-1.987420	-0.488603	0.576116	-0.392076
4	H	-1.673507	1.684443	-0.135607	0.390955
5	C	0.181173	-0.940797	-0.395675	-0.048325
6	N	1.452353	-0.269590	-0.560196	-0.539640
7	H	0.264995	-1.728444	0.358806	0.120825
8	H	-0.109768	-1.424483	-1.336055	0.128642
9	C	1.930717	0.320317	0.686224	-0.138671
10	H	1.345992	0.464395	-1.256202	0.303147
11	H	2.862833	0.858278	0.498794	0.101458
12	H	1.219767	1.019214	1.152851	0.080402

13	H	2.140088	-0.477924	1.405266	0.096651

2b

log file: s1b.log
E(RM06-2X) = -323.616785673 A.U.
Zero-point correction= 0.310959
Thermal correction to Energy= 0.331562
Thermal correction to Enthalpy= 0.332506
Thermal correction to Gibbs Free Energy= 0.262067
Sum of electronic and zero-point Energies= -991.203362
Sum of electronic and thermal Energies= -991.182759
Sum of electronic and thermal Enthalpies= -991.181815
Sum of electronic and thermal Free Energies= -991.252255

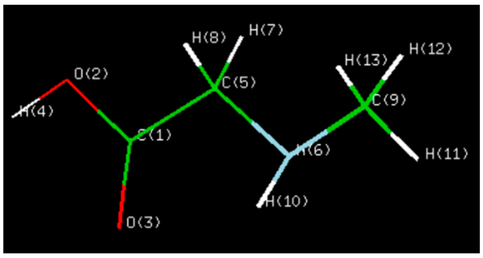

		x	y	z	q
1	C	1.111339	0.076424	-0.001398	0.277226
2	O	2.091167	-0.832393	-0.010085	-0.382174
3	O	1.314272	1.270544	0.012056	-0.377778
4	H	2.944960	-0.365628	0.007216	0.390396
5	C	-0.250185	-0.566255	0.027566	-0.047784
6	N	-1.289486	0.419247	-0.182132	-0.541289
7	H	-0.286817	-1.330176	-0.757670	0.122522
8	H	-0.323655	-1.106344	0.988985	0.111523
9	C	-2.611101	-0.157252	0.028713	-0.134043
10	H	-1.144913	1.173755	0.485245	0.300882
11	H	-3.369143	0.622935	-0.071265	0.101001
12	H	-2.805229	-0.916159	-0.736109	0.096233
13	H	-2.732634	-0.635826	1.013479	0.083286

2c

log file: s1d.log
E(RM06-2X) = -323.615757304 A.U.
Zero-point correction= 0.310959
Thermal correction to Energy= 0.331562
Thermal correction to Enthalpy= 0.332506
Thermal correction to Gibbs Free Energy= 0.262067
Sum of electronic and zero-point Energies= -991.203362
Sum of electronic and thermal Energies= -991.182759
Sum of electronic and thermal Enthalpies= -991.181815
Sum of electronic and thermal Free Energies= -991.252255

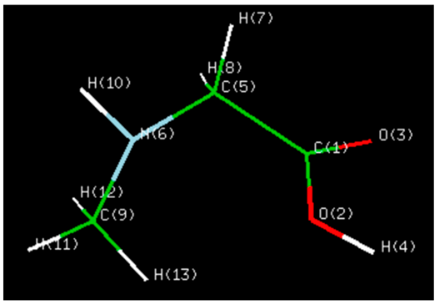

		x	y	z	q
1	C	-1.015298	-0.076347	0.001125	0.271409

2	O	-0.949464	1.184374	0.432362	-0.375985
3	O	-1.980805	-0.522131	-0.583185	-0.394850
4	H	-1.780343	1.628752	0.187989	0.389320
5	C	0.196884	-0.913545	0.349130	-0.046117
6	N	1.395508	-0.134898	0.579908	-0.536773
7	H	-0.070537	-1.452659	1.265498	0.133296
8	H	0.286504	-1.661584	-0.454041	0.106323
9	C	1.958761	0.372750	-0.666282	-0.136860
10	H	2.078709	-0.739342	1.027082	0.310819
11	H	2.902138	0.883625	-0.460477	0.101880
12	H	2.142595	-0.412996	-1.415710	0.084083
13	H	1.272445	1.103390	-1.106947	0.093455

2d

log file: s1.log
E(RM06-2X) = -323.614885779 A.U.
Zero-point correction= 0.310959
Thermal correction to Energy= 0.331562
Thermal correction to Enthalpy= 0.332506
Thermal correction to Gibbs Free Energy= 0.262067
Sum of electronic and zero-point Energies= -991.203362
Sum of electronic and thermal Energies= -991.182759
Sum of electronic and thermal Enthalpies= -991.181815
Sum of electronic and thermal Free Energies= -991.252255

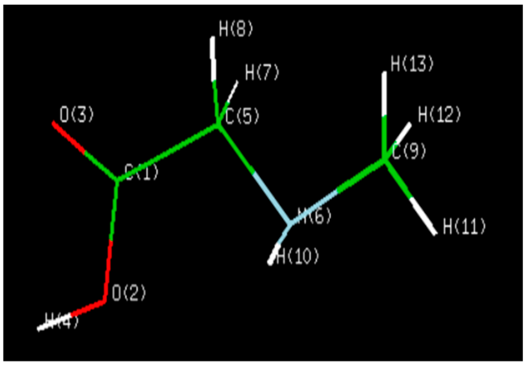

		x	y	z	q
1	C	1.168516	-0.125047	-0.000850	0.276001
2	O	1.290186	1.203411	0.025342	-0.370835
3	O	2.119668	-0.874669	-0.065536	-0.393612
4	H	2.236928	1.425700	-0.014784	0.391845
5	C	-0.257948	-0.602482	0.110482	-0.047007
6	N	-1.244022	0.419323	-0.186022	-0.542483
7	H	-0.366554	-1.436655	-0.590290	0.123366
8	H	-0.352276	-1.034491	1.123225	0.113135
9	C	-2.594607	-0.103364	-0.011915	-0.133871
10	H	-1.108902	1.190103	0.464326	0.301622
11	H	-3.318666	0.699617	-0.166969	0.101258
12	H	-2.784813	-0.879747	-0.759866	0.096739
13	H	-2.772151	-0.544370	0.981769	0.083839

3

log file: on1.log
E(RM06-2X) = -460.867158353 A.U.
Zero-point correction= 0.310959
Thermal correction to Energy= 0.331562
Thermal correction to Enthalpy= 0.332506
Thermal correction to Gibbs Free Energy= 0.262067
Sum of electronic and zero-point Energies= -991.203362

Sum of electronic and thermal Energies= -991.182759
Sum of electronic and thermal Enthalpies= -991.181815
Sum of electronic and thermal Free Energies= -991.252255

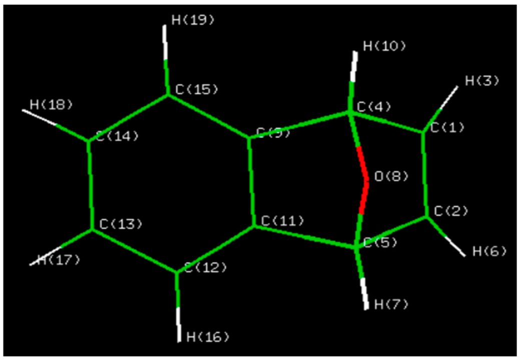

		x	y	z	q
1	C	-1.980954	0.665378	0.948938	-0.107349
2	C	-1.980947	-0.665737	0.948727	-0.107337
3	H	-2.325633	1.352702	1.710530	0.121114
4	C	-1.261093	1.073127	-0.346846	0.026096
5	C	-1.261143	-1.072911	-0.347312	0.026084
6	H	-2.325354	-1.353457	1.710115	0.121127
7	H	-1.486690	-2.048356	-0.774050	0.128244
8	O	-1.667077	0.000249	-1.219006	-0.292864
9	C	0.215663	0.701477	-0.159842	-0.028426
10	H	-1.486510	2.048718	-0.773331	0.128251
11	C	0.215616	-0.701494	-0.159864	-0.028421
12	C	1.380328	-1.418921	0.003331	-0.103871
13	C	2.576312	-0.694684	0.163266	-0.109399
14	C	2.576337	0.694625	0.163283	-0.109400
15	C	1.380372	1.418895	0.003348	-0.103870
16	H	1.386234	-2.505052	0.003549	0.111985
17	H	3.512670	-1.230817	0.284668	0.108029
18	H	3.512716	1.230709	0.284717	0.108027
19	H	1.386238	2.505027	0.003680	0.111988

23

log file: im1.log
E(RM06-2X) = -571.579624304 A.U.
Zero-point correction= 0.310959
Thermal correction to Energy= 0.331562
Thermal correction to Enthalpy= 0.332506
Thermal correction to Gibbs Free Energy= 0.262067
Sum of electronic and zero-point Energies= -991.203362
Sum of electronic and thermal Energies= -991.182759
Sum of electronic and thermal Enthalpies= -991.181815
Sum of electronic and thermal Free Energies= -991.252255

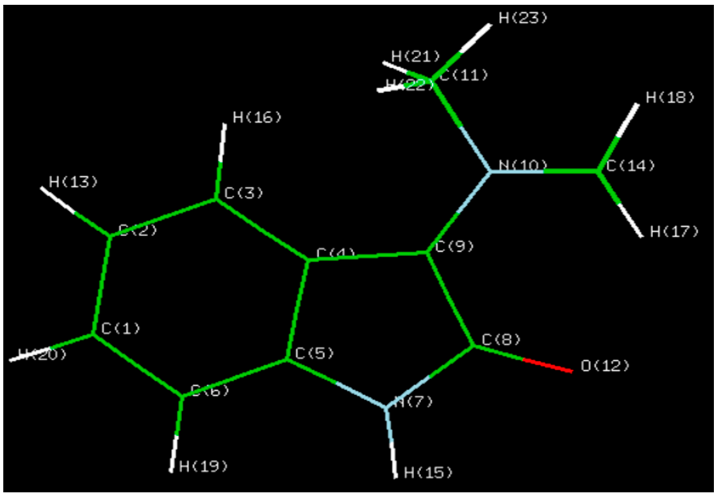

		x	y	z	q
1	C	3.336564	0.170095	-0.000010	-0.129880
2	C	2.689721	1.407584	-0.000014	-0.133025
3	C	1.298311	1.504435	-0.000006	-0.125823
4	C	0.517026	0.337257	0.000003	-0.043812
5	C	1.208120	-0.908677	0.000013	0.075098
6	C	2.591759	-1.008238	0.000011	-0.121903
7	N	0.278142	-1.930929	0.000076	-0.431315
8	C	-1.015893	-1.438717	-0.000000	0.205282
9	C	-0.887905	-0.007783	-0.000004	0.007814
10	N	-1.997077	0.827637	0.000006	-0.209869
11	C	-1.730715	2.276286	-0.000005	-0.088151
12	O	-2.031073	-2.164642	-0.000049	-0.459847
13	H	3.277550	2.320450	-0.000026	0.100921
14	C	-3.235259	0.432452	0.000020	0.009535
15	H	0.489129	-2.919527	-0.000223	0.357158
16	H	0.851388	2.490387	-0.000015	0.102659
17	H	-3.446942	-0.626613	0.000032	0.131135
18	H	-4.009836	1.186991	0.000015	0.144810
19	H	3.070177	-1.983001	0.000022	0.112077
20	H	4.420799	0.121902	-0.000019	0.102616
21	H	-1.161387	2.526444	0.895205	0.130133
22	H	-1.161386	2.526436	-0.895217	0.130133
23	H	-2.678751	2.808552	-0.000003	0.134266

7

log file: im1b.log
E(RM06-2X) = -571.577749141 A.U.
Zero-point correction= 0.310959
Thermal correction to Energy= 0.331562
Thermal correction to Enthalpy= 0.332506
Thermal correction to Gibbs Free Energy= 0.262067
Sum of electronic and zero-point Energies= -991.203362
Sum of electronic and thermal Energies= -991.182759
Sum of electronic and thermal Enthalpies= -991.181815
Sum of electronic and thermal Free Energies= -991.252255

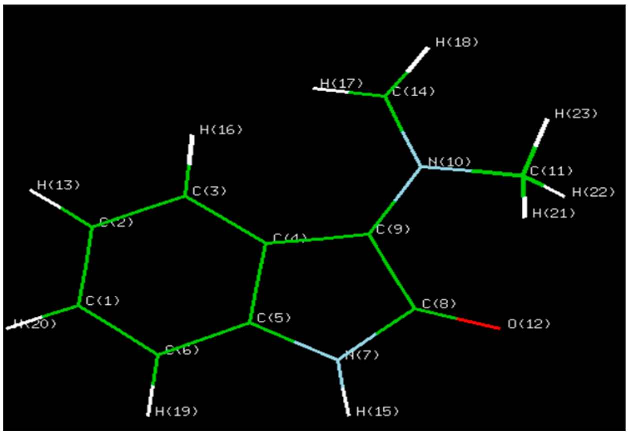

		x	y	z	q
1	C	3.364081	0.154232	-0.064713	-0.127784
2	C	2.737201	1.399849	-0.116098	-0.131668
3	C	1.345990	1.515679	-0.075694	-0.124463
4	C	0.560433	0.358663	0.014269	-0.045467
5	C	1.226001	-0.898650	0.040801	0.077260
6	C	2.606400	-1.016701	0.008415	-0.121282
7	N	0.270027	-1.896347	0.077234	-0.432243
8	C	-1.018594	-1.378952	0.056173	0.206682
9	C	-0.847732	0.051754	0.022331	0.007343
10	N	-1.902780	0.949824	0.032796	-0.207049
11	C	-3.236150	0.442947	-0.359996	-0.095951
12	O	-2.036567	-2.094903	0.079542	-0.470050
13	H	3.338353	2.301266	-0.184934	0.101547
14	C	-1.794369	2.203360	0.347711	0.018830
15	H	0.455360	-2.889859	0.096105	0.356124
16	H	0.903643	2.503397	-0.135300	0.103402
17	H	-0.858229	2.580971	0.730523	0.141340
18	H	-2.660380	2.843563	0.254660	0.149168
19	H	3.076364	-1.995143	0.033426	0.112178
20	H	4.447359	0.090476	-0.086683	0.103043
21	H	-3.143454	-0.091902	-1.303614	0.125140
22	H	-3.587261	-0.242337	0.408398	0.123674
23	H	-3.909523	1.291361	-0.458330	0.130235

15

```
log file: geo7.log
E(RM06-2X) =  -836.517504903     A.U.
Zero-point correction=                           0.310959
Thermal correction to Energy=                    0.331562
Thermal correction to Enthalpy=                  0.332506
Thermal correction to Gibbs Free Energy=         0.262067
Sum of electronic and zero-point Energies=          -991.203362
Sum of electronic and thermal Energies=             -991.182759
Sum of electronic and thermal Enthalpies=           -991.181815
Sum of electronic and thermal Free Energies=        -991.252255
```

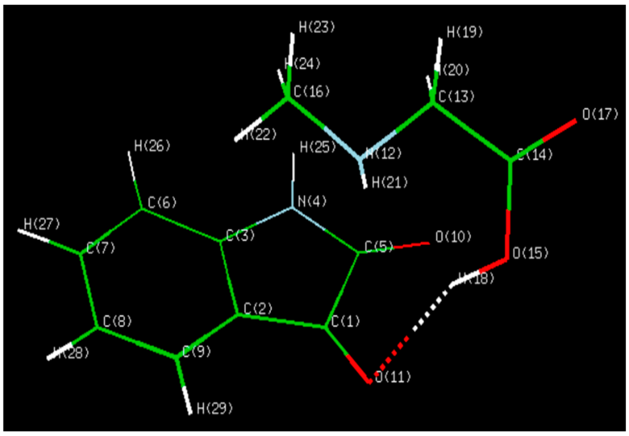

		x	y	z	q
1	C	0.316783	-1.299708	-0.027047	0.195855
2	C	1.655680	-0.739385	-0.199276	-0.039358
3	C	1.865884	0.152667	0.864590	0.117231
4	N	0.749155	0.180996	1.715534	-0.404948
5	C	-0.238730	-0.643380	1.269155	0.275806
6	C	3.036568	0.885137	0.966183	-0.094584
7	C	3.994415	0.696152	-0.034174	-0.069378
8	C	3.795876	-0.187936	-1.099964	-0.103140
9	C	2.612901	-0.917808	-1.190661	-0.062923
10	O	-1.325821	-0.859166	1.766531	-0.352332
11	O	-0.304465	-2.112281	-0.682342	-0.308074
12	N	-1.056273	0.928163	-0.868922	-0.495137
13	C	-2.313205	1.288086	-0.235676	-0.047616
14	C	-3.365356	0.197368	-0.277710	0.263311
15	O	-3.004680	-1.032826	-0.637293	-0.397681
16	C	-0.122600	2.053044	-0.864722	-0.129414
17	O	-4.524123	0.430458	-0.008577	-0.399681
18	H	-2.037113	-1.146235	-0.696138	0.332553
19	H	-2.783664	2.182407	-0.669427	0.116954
20	H	-2.129561	1.505757	0.824727	0.124936
21	H	-1.235665	0.665968	-1.838148	0.319427
22	H	0.790034	1.772343	-1.396246	0.099222
23	H	-0.542466	2.953004	-1.336244	0.093661
24	H	0.139107	2.303189	0.168508	0.097020
25	H	0.675471	0.755436	2.547004	0.374129
26	H	3.199816	1.579311	1.783236	0.127334
27	H	4.921348	1.258826	0.019799	0.122495
28	H	4.565678	-0.301414	-1.855137	0.116544
29	H	2.430254	-1.607604	-2.008957	0.127762

TS1

log file: geo7_ts1.log
E(RM06-2X) = -836.510918260 A.U.
Zero-point correction= 0.310959
Thermal correction to Energy= 0.331562
Thermal correction to Enthalpy= 0.332506
Thermal correction to Gibbs Free Energy= 0.262067
Sum of electronic and zero-point Energies= -991.203362
Sum of electronic and thermal Energies= -991.182759
Sum of electronic and thermal Enthalpies= -991.181815
Sum of electronic and thermal Free Energies= -991.252255

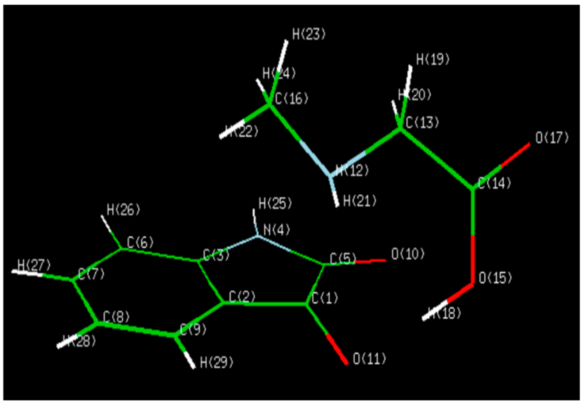

		x	y	z	q
1	C	-0.009287	-0.108020	-0.843434	0.187273
2	C	-1.452625	-0.268099	-0.538867	-0.035917
3	C	-1.882042	0.900365	0.100541	0.104824
4	N	-0.816725	1.815143	0.200361	-0.404453
5	C	0.307580	1.348103	-0.410217	0.272112
6	C	-3.188142	1.049008	0.535380	-0.097741
7	C	-4.061515	-0.018487	0.303108	-0.082942
8	C	-3.646359	-1.184719	-0.342959	-0.105404
9	C	-2.324054	-1.317789	-0.773589	-0.073756
10	O	1.365018	1.927665	-0.574978	-0.360912
11	O	0.601994	-0.678093	-1.784803	-0.399445
12	N	0.818952	-0.888715	0.775948	-0.396125
13	C	2.135403	-0.349904	1.159619	-0.041194
14	C	3.239415	-0.271584	0.110141	0.253368
15	O	3.011681	-0.539492	-1.153354	-0.409771
16	C	-0.045485	-0.981770	1.964131	-0.115040
17	O	4.354811	0.022947	0.500508	-0.416452
18	H	2.021935	-0.591120	-1.441451	0.312913
19	H	2.546875	-0.957287	1.973460	0.134889
20	H	1.996321	0.661683	1.552578	0.132969
21	H	0.938876	-1.821841	0.372834	0.346331
22	H	-0.969727	-1.497432	1.705769	0.111550
23	H	0.463964	-1.530079	2.762342	0.117223
24	H	-0.271486	0.028223	2.316498	0.112010
25	H	-0.902719	2.757415	0.563877	0.372602
26	H	-3.519461	1.955113	1.031060	0.125042
27	H	-5.092248	0.068449	0.632581	0.118514
28	H	-4.355285	-1.988271	-0.510485	0.114080
29	H	-1.978008	-2.216677	-1.275341	0.123445

16

log file: geo9.log
E(RM06-2X) = -836.520266468 A.U.
Zero-point correction= 0.310959
Thermal correction to Energy= 0.331562
Thermal correction to Enthalpy= 0.332506
Thermal correction to Gibbs Free Energy= 0.262067
Sum of electronic and zero-point Energies= -991.203362
Sum of electronic and thermal Energies= -991.182759
Sum of electronic and thermal Enthalpies= -991.181815
Sum of electronic and thermal Free Energies= -991.252255

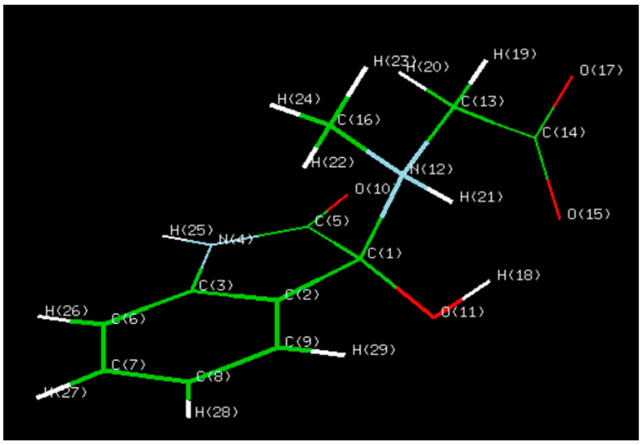

		x	y	z	q
1	C	0.079692	-0.121275	-0.423804	0.194206
2	C	-1.411835	-0.292654	-0.297404	-0.031668
3	C	-1.963031	0.912019	0.141808	0.101067
4	N	-0.945342	1.865463	0.324256	-0.401441
5	C	0.273946	1.385177	-0.034185	0.279832
6	C	-3.328274	1.067382	0.316414	-0.097640
7	C	-4.137572	-0.034772	0.025824	-0.086055
8	C	-3.599944	-1.237570	-0.432979	-0.105016
9	C	-2.219474	-1.372540	-0.606813	-0.077758
10	O	1.321684	1.999374	-0.091852	-0.353272
11	O	0.534241	-0.426143	-1.667333	-0.453143
12	N	0.859170	-0.998167	0.600827	-0.327106
13	C	2.250521	-0.532252	0.963523	-0.042644
14	C	3.269645	-0.217108	-0.145462	0.210821
15	O	2.961745	-0.355285	-1.369240	-0.440899
16	C	0.118281	-1.253567	1.875841	-0.096196
17	O	4.370609	0.128185	0.296454	-0.478353
18	H	1.583523	-0.368899	-1.638274	0.301308
19	H	2.672603	-1.339872	1.564524	0.142373
20	H	2.141138	0.346566	1.599065	0.132980
21	H	0.954352	-1.893926	0.103228	0.379353
22	H	-0.788684	-1.813505	1.665513	0.125418
23	H	0.770169	-1.829228	2.531113	0.136490
24	H	-0.117655	-0.293277	2.337445	0.129895
25	H	-1.106016	2.842894	0.541595	0.374419
26	H	-3.749467	2.007398	0.655790	0.125515
27	H	-5.211693	0.055135	0.154013	0.118268
28	H	-4.256763	-2.069967	-0.661016	0.114440
29	H	-1.786272	-2.300478	-0.969375	0.124792

TS2

log file: geo9_ts3.log
E(RM06-2X) = -836.511528434 A.U.
Zero-point correction= 0.310959
Thermal correction to Energy= 0.331562
Thermal correction to Enthalpy= 0.332506
Thermal correction to Gibbs Free Energy= 0.262067
Sum of electronic and zero-point Energies= -991.203362
Sum of electronic and thermal Energies= -991.182759
Sum of electronic and thermal Enthalpies= -991.181815
Sum of electronic and thermal Free Energies= -991.252255

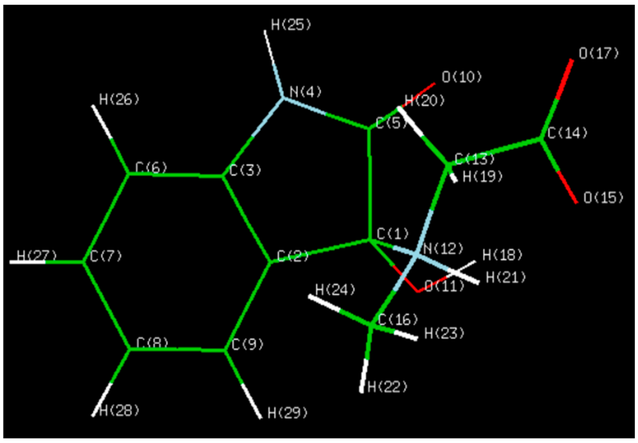

		x	y	z	q
1	C	-0.050372	-0.202736	0.644876	0.202819
2	C	1.414392	-0.238525	0.293762	-0.029621
3	C	1.765093	1.004651	-0.241361	0.103395
4	N	0.660030	1.873703	-0.212516	-0.400553
5	C	-0.399834	1.308396	0.416687	0.284439
6	C	3.053609	1.287456	-0.660229	-0.096625
7	C	4.011167	0.282101	-0.503803	-0.082539
8	C	3.688266	-0.947130	0.068891	-0.104141
9	C	2.379516	-1.215391	0.479004	-0.075339
10	O	-1.419592	1.846682	0.792845	-0.344545
11	O	-0.306527	-0.574660	1.948082	-0.410798
12	N	-0.930115	-1.100814	-0.266016	-0.340500
13	C	-1.859240	-0.379774	-1.188722	-0.048426
14	C	-3.132540	-0.037251	-0.374634	0.190336
15	O	-3.208087	-0.611834	0.747032	-0.475071
16	C	-0.222897	-2.241391	-0.906167	-0.095863
17	O	-3.950777	0.715851	-0.922062	-0.501995
18	H	-1.211297	-0.280918	2.168869	0.358944
19	H	-2.127141	-1.065882	-1.994683	0.140585
20	H	-1.383667	0.496845	-1.628220	0.128580
21	H	-1.620540	-1.478818	0.419393	0.341098
22	H	0.304659	-2.807186	-0.140388	0.129936
23	H	-0.983603	-2.873298	-1.363789	0.135711
24	H	0.466508	-1.867696	-1.661878	0.124576
25	H	0.713422	2.869397	-0.398673	0.377021
26	H	3.308583	2.257395	-1.072921	0.126943
27	H	5.031539	0.473503	-0.820487	0.119870
28	H	4.457378	-1.699891	0.202460	0.115665
29	H	2.131655	-2.164433	0.943038	0.126128

17

log file: geo9_ts2_m1.log

E(RM06-2X) = -836.520905763 A.U.

Zero-point correction= 0.310959

Thermal correction to Energy= 0.331562

Thermal correction to Enthalpy= 0.332506

Thermal correction to Gibbs Free Energy= 0.262067

Sum of electronic and zero-point Energies= -991.203362

Sum of electronic and thermal Energies= -991.182759

Sum of electronic and thermal Enthalpies= -991.181815

Sum of electronic and thermal Free Energies= -991.252255

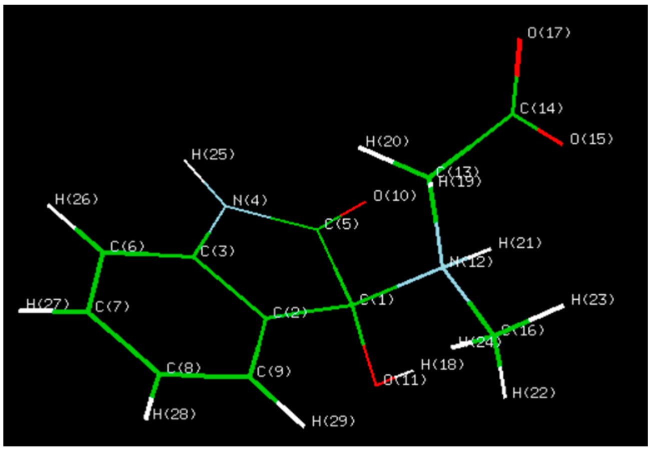

		x	y	z	q
1	C	-0.072140	-0.017320	1.010867	0.201593
2	C	-1.358214	0.269186	0.286614	-0.031344
3	C	-1.770684	-0.915666	-0.333939	0.102708
4	N	-0.846982	-1.947050	-0.075526	-0.395218
5	C	0.172946	-1.524216	0.706202	0.281737
6	C	-2.941597	-0.988408	-1.067829	-0.094734
7	C	-3.712593	0.174232	-1.154442	-0.082623
8	C	-3.328351	1.354326	-0.519558	-0.102949
9	C	-2.141711	1.409150	0.217765	-0.077541
10	O	1.123524	-2.144932	1.140000	-0.357725
11	O	-0.164023	0.204924	2.369836	-0.399974
12	N	1.126693	0.724784	0.465056	-0.344887
13	C	1.414099	0.499796	-0.983794	-0.049515
14	C	2.916936	0.142110	-1.100845	0.196247
15	O	3.501436	-0.047437	-0.001178	-0.476370
16	C	1.152803	2.170560	0.817989	-0.097443
17	O	3.371908	0.068274	-2.252586	-0.503066
18	H	0.535344	-0.299763	2.821726	0.379283
19	H	1.172492	1.398774	-1.550968	0.136211
20	H	0.815626	-0.327789	-1.370842	0.127782
21	H	1.990546	0.295219	0.890953	0.330948
22	H	0.990120	2.277530	1.888244	0.127483
23	H	2.140605	2.544043	0.546235	0.134194
24	H	0.384377	2.691324	0.247492	0.127012
25	H	-0.946542	-2.904881	-0.393941	0.379414
26	H	-3.248314	-1.911698	-1.546890	0.127620
27	H	-4.636664	0.150005	-1.723202	0.119938
28	H	-3.956644	2.235284	-0.591847	0.115882
29	H	-1.850670	2.318697	0.733571	0.125335

TS3

log file: geo9_ts2.log
E(RM06-2X) = -836.518498853 A.U.
Zero-point correction= 0.310959
Thermal correction to Energy= 0.331562
Thermal correction to Enthalpy= 0.332506
Thermal correction to Gibbs Free Energy= 0.262067
Sum of electronic and zero-point Energies= -991.203362
Sum of electronic and thermal Energies= -991.182759
Sum of electronic and thermal Enthalpies= -991.181815
Sum of electronic and thermal Free Energies= -991.252255

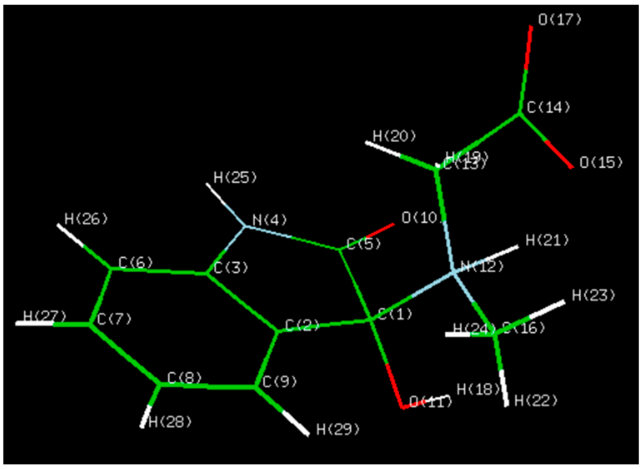

		x	y	z	q
1	C	-0.029255	0.156511	0.924540	0.197214
2	C	-1.367179	0.295373	0.243698	-0.031780
3	C	-1.768936	-0.975833	-0.181772	0.100219
4	N	-0.800701	-1.933739	0.174575	-0.398696
5	C	0.239661	-1.374960	0.837991	0.278828
6	C	-2.970204	-1.185239	-0.836666	-0.097745
7	C	-3.784318	-0.068927	-1.048773	-0.086323
8	C	-3.410335	1.199630	-0.608830	-0.105825
9	C	-2.192360	1.391139	0.051749	-0.081121
10	O	1.216253	-1.916819	1.318774	-0.363975
11	O	-0.082008	0.562747	2.250082	-0.409853
12	N	1.101387	0.849139	0.240788	-0.341447
13	C	1.427364	0.370153	-1.125988	-0.048531
14	C	2.916381	-0.035745	-1.027320	0.218637
15	O	3.350465	0.075226	0.175962	-0.433846
16	C	1.033198	2.325246	0.319376	-0.108069
17	O	3.529472	-0.408980	-2.022359	-0.459844
18	H	0.694871	0.204587	2.713961	0.374466
19	H	1.281480	1.156321	-1.867929	0.138285
20	H	0.826609	-0.495846	-1.414642	0.129989
21	H	2.187320	0.497095	0.647022	0.307229
22	H	0.819040	2.617318	1.346198	0.120633
23	H	2.010124	2.713109	0.025051	0.126671
24	H	0.268136	2.707685	-0.358798	0.116824
25	H	-0.889306	-2.931132	0.014147	0.376761
26	H	-3.267667	-2.176164	-1.162162	0.125881
27	H	-4.732982	-0.200063	-1.559673	0.118391
28	H	-4.070543	2.044106	-0.774058	0.114344
29	H	-1.909452	2.373702	0.415643	0.122693

18

log file: geo9_ts2_m2.log
E(RM06-2X) = -836.529713854 A.U.
Zero-point correction= 0.310959
Thermal correction to Energy= 0.331562
Thermal correction to Enthalpy= 0.332506
Thermal correction to Gibbs Free Energy= 0.262067
Sum of electronic and zero-point Energies= -991.203362
Sum of electronic and thermal Energies= -991.182759
Sum of electronic and thermal Enthalpies= -991.181815
Sum of electronic and thermal Free Energies= -991.252255

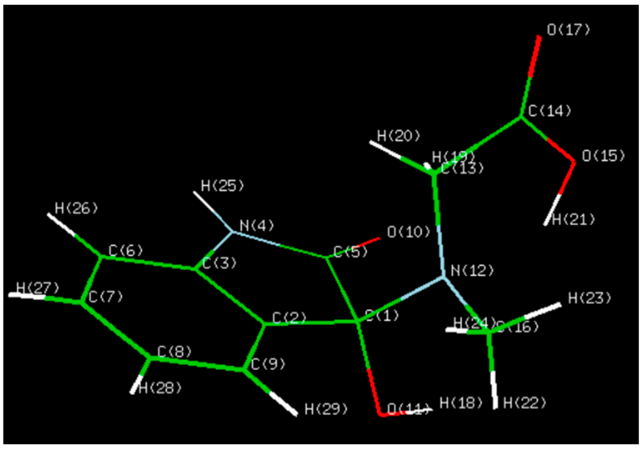

		x	y	z	q
1	C	-0.061543	-0.076533	0.951021	0.188969
2	C	-1.386517	0.256636	0.297736	-0.033181
3	C	-1.839007	-0.880937	-0.380141	0.095538
4	N	-0.925765	-1.938870	-0.208458	-0.404025
5	C	0.141963	-1.565250	0.539577	0.273178
6	C	-3.028705	-0.892356	-1.087968	-0.102599
7	C	-3.778718	0.287820	-1.096196	-0.092668
8	C	-3.351980	1.424756	-0.412690	-0.110319
9	C	-2.146241	1.414445	0.298154	-0.087146
10	O	1.101536	-2.239920	0.868076	-0.376752
11	O	-0.186313	0.002048	2.342267	-0.426307
12	N	1.114916	0.666764	0.514481	-0.350814
13	C	1.449013	0.529464	-0.898398	-0.042949
14	C	2.938081	0.257231	-1.067460	0.262512
15	O	3.556379	-0.145368	0.036738	-0.393652
16	C	1.151086	2.066112	0.942200	-0.124275
17	O	3.516493	0.362727	-2.126191	-0.398105
18	H	0.630341	-0.359536	2.726277	0.364268
19	H	1.187862	1.415252	-1.488321	0.127234
20	H	0.932778	-0.319781	-1.360668	0.132863
21	H	2.873777	-0.138014	0.748230	0.340300
22	H	0.905091	2.132938	2.001836	0.109094
23	H	2.167529	2.443186	0.796592	0.110852
24	H	0.461717	2.695665	0.363569	0.097246
25	H	-1.048391	-2.873089	-0.581892	0.372378
26	H	-3.365209	-1.782511	-1.608754	0.122830
27	H	-4.716261	0.311622	-1.642791	0.115587
28	H	-3.960613	2.322646	-0.426930	0.111658
29	H	-1.822026	2.292136	0.848557	0.118318

TS4

log file: geo9_ts4.log
E(RM06-2X) = -836.521860371 A.U.
Zero-point correction= 0.310959
Thermal correction to Energy= 0.331562
Thermal correction to Enthalpy= 0.332506
Thermal correction to Gibbs Free Energy= 0.262067
Sum of electronic and zero-point Energies= -991.203362
Sum of electronic and thermal Energies= -991.182759
Sum of electronic and thermal Enthalpies= -991.181815
Sum of electronic and thermal Free Energies= -991.252255

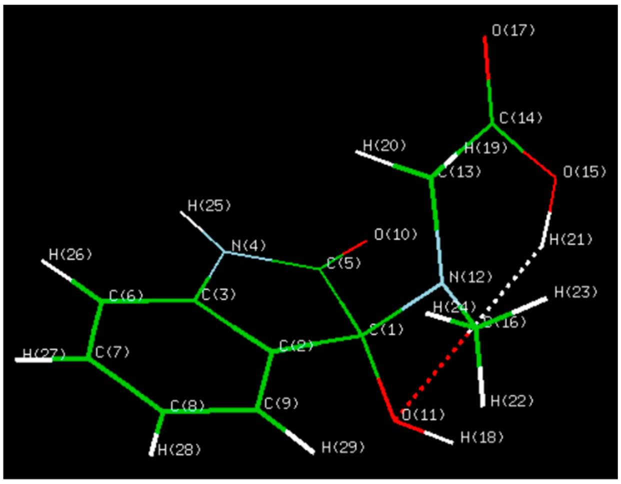

		x	y	z	q
1	C	-0.004529	0.374156	0.797870	0.188879
2	C	-1.409306	0.296524	0.216319	-0.036057
3	C	-1.711130	-1.047543	-0.027485	0.094602
4	N	-0.631657	-1.859929	0.367388	-0.404055
5	C	0.388775	-1.132777	0.883887	0.275764
6	C	-2.925508	-1.452460	-0.554503	-0.103386
7	C	-3.867188	-0.456066	-0.828629	-0.093875
8	C	-3.597295	0.885611	-0.567792	-0.111307
9	C	-2.362053	1.271009	-0.033805	-0.088587
10	O	1.442552	-1.544860	1.332554	-0.371729
11	O	0.013326	0.857838	2.117359	-0.446926
12	N	0.994349	1.067945	-0.015793	-0.345828
13	C	1.534848	0.309425	-1.144002	-0.042386
14	C	2.931583	-0.220929	-0.844941	0.254925
15	O	3.502917	0.275526	0.244757	-0.395858
16	C	0.613600	2.431553	-0.399021	-0.117882
17	O	3.508743	-0.994347	-1.577423	-0.400456
18	H	0.328552	1.772013	2.119227	0.362150
19	H	1.640942	0.966209	-2.013132	0.141829
20	H	0.907236	-0.526626	-1.471049	0.125245
21	H	2.810501	0.808215	0.698290	0.344763
22	H	0.211723	2.974920	0.460061	0.118774
23	H	1.510250	2.957505	-0.734222	0.114849
24	H	-0.131091	2.447664	-1.204605	0.097925
25	H	-0.622976	-2.872107	0.319745	0.372718
26	H	-3.138298	-2.500733	-0.735512	0.122536
27	H	-4.830009	-0.739706	-1.241971	0.115204
28	H	-4.351975	1.637427	-0.773010	0.111119
29	H	-2.164767	2.314835	0.189634	0.117056

19

```
log file: geo9_ts4_m2.log
E(RM06-2X) =  -836.526681002     A.U.
Zero-point correction=                          0.310959
Thermal correction to Energy=                   0.331562
Thermal correction to Enthalpy=                 0.332506
Thermal correction to Gibbs Free Energy=        0.262067
Sum of electronic and zero-point Energies=          -991.203362
Sum of electronic and thermal Energies=             -991.182759
Sum of electronic and thermal Enthalpies=           -991.181815
Sum of electronic and thermal Free Energies=        -991.252255
```

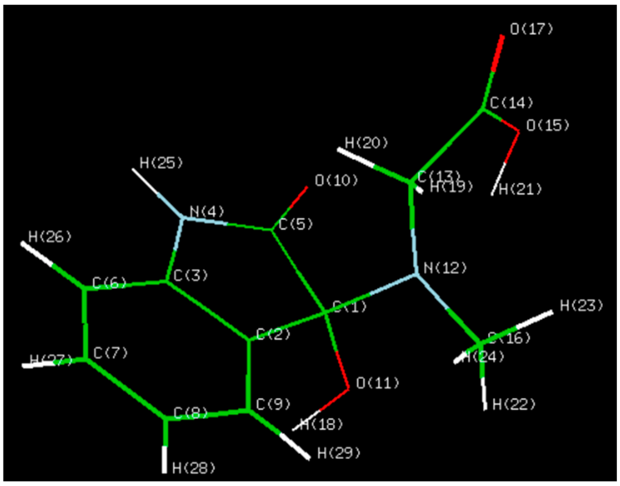

		x	y	z	q
1	C	-0.052878	0.049491	0.945968	0.191461
2	C	-1.384604	0.282451	0.255554	-0.034357
3	C	-1.818896	-0.932994	-0.285857	0.095759
4	N	-0.887627	-1.948434	0.000922	-0.406489
5	C	0.176960	-1.476240	0.700085	0.270709
6	C	-3.012288	-1.045389	-0.979016	-0.102701
7	C	-3.784911	0.112276	-1.114253	-0.091900
8	C	-3.377828	1.326723	-0.565025	-0.110579
9	C	-2.169170	1.418206	0.134636	-0.085678
10	O	1.150636	-2.098009	1.082734	-0.378588
11	O	-0.071862	0.306014	2.325586	-0.425776
12	N	1.107334	0.754638	0.425161	-0.346786
13	C	1.462924	0.441332	-0.953786	-0.042882
14	C	2.947973	0.115974	-1.055303	0.261534
15	O	3.541711	-0.127032	0.106409	-0.393827
16	C	1.114746	2.198327	0.668025	-0.124590
17	O	3.542346	0.052844	-2.109011	-0.399983
18	H	-0.960158	0.122961	2.663278	0.369295
19	H	1.239645	1.260525	-1.646085	0.128334
20	H	0.931708	-0.439268	-1.331706	0.132328
21	H	2.845436	-0.006772	0.795976	0.339227
22	H	0.877386	2.397209	1.712398	0.107250
23	H	2.121956	2.572539	0.462649	0.111181
24	H	0.407864	2.732324	0.018617	0.096489
25	H	-0.991655	-2.919063	-0.271526	0.371355
26	H	-3.334968	-1.994584	-1.393386	0.122835
27	H	-4.725486	0.056698	-1.653334	0.115874
28	H	-4.004410	2.205399	-0.675121	0.111763
29	H	-1.860095	2.357112	0.583753	0.118746

TS5

log file: geo9_ts9.log

E(RM06-2X) = -836.520375163 A.U.

Zero-point correction=	0.310959
Thermal correction to Energy=	0.331562
Thermal correction to Enthalpy=	0.332506
Thermal correction to Gibbs Free Energy=	0.262067
Sum of electronic and zero-point Energies=	-991.203362
Sum of electronic and thermal Energies=	-991.182759
Sum of electronic and thermal Enthalpies=	-991.181815
Sum of electronic and thermal Free Energies=	-991.252255

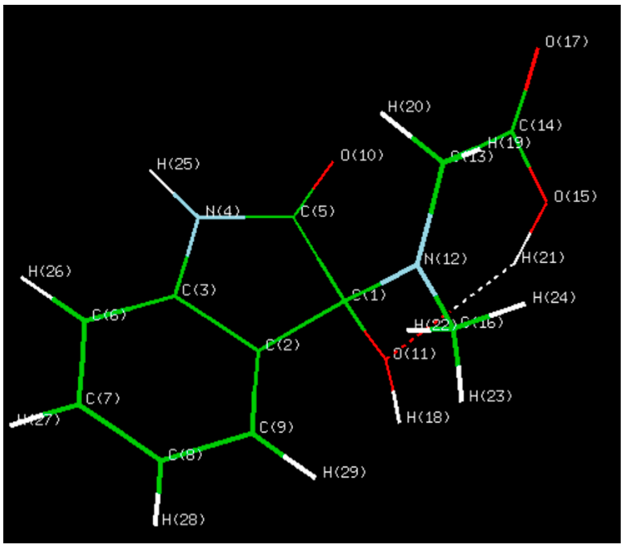

		x	y	z	q
1	C	0.036455	0.167289	0.527103	0.193738
2	C	-1.450429	0.264449	0.220607	-0.029597
3	C	-1.901301	-1.002043	-0.168068	0.095075
4	N	-0.843846	-1.924013	-0.101079	-0.402728
5	C	0.300495	-1.354751	0.349916	0.282413
6	C	-3.214762	-1.243919	-0.530959	-0.103179
7	C	-4.100705	-0.163036	-0.487585	-0.094720
8	C	-3.677038	1.101017	-0.085580	-0.111176
9	C	-2.342142	1.323239	0.274438	-0.088059
10	O	1.356398	-1.912654	0.588769	-0.366971
11	O	0.341841	0.422492	1.892925	-0.436477
12	N	0.883722	0.931545	-0.348526	-0.357841
13	C	1.902453	0.324493	-1.171064	-0.039625
14	C	3.188580	0.011080	-0.412013	0.252047
15	O	3.255697	0.439015	0.845870	-0.398028
16	C	0.536469	2.322471	-0.588429	-0.116900
17	O	4.123087	-0.562410	-0.928746	-0.405089
18	H	-0.076008	1.258422	2.149265	0.364475
19	H	2.175883	1.021405	-1.968637	0.139460
20	H	1.589130	-0.598565	-1.670162	0.122913
21	H	2.388473	0.815676	1.101046	0.342034
22	H	-0.237580	2.448366	-1.357581	0.091155
23	H	0.187892	2.790103	0.337739	0.112008
24	H	1.432252	2.863207	-0.902364	0.114083
25	H	-0.935361	-2.920596	-0.262134	0.372881
26	H	-3.541040	-2.234943	-0.828061	0.122705
27	H	-5.138371	-0.319170	-0.765239	0.115252
28	H	-4.385458	1.921764	-0.048163	0.111369
29	H	-2.023584	2.308332	0.600773	0.118760

20

log file: geo11_ts1_m1.log

E(RM06-2X) = -836.525496652 A.U.

Zero-point correction=	0.310959
Thermal correction to Energy=	0.331562
Thermal correction to Enthalpy=	0.332506
Thermal correction to Gibbs Free Energy=	0.262067
Sum of electronic and zero-point Energies=	-991.203362
Sum of electronic and thermal Energies=	-991.182759
Sum of electronic and thermal Enthalpies=	-991.181815

Sum of electronic and thermal Free Energies= -991.252255

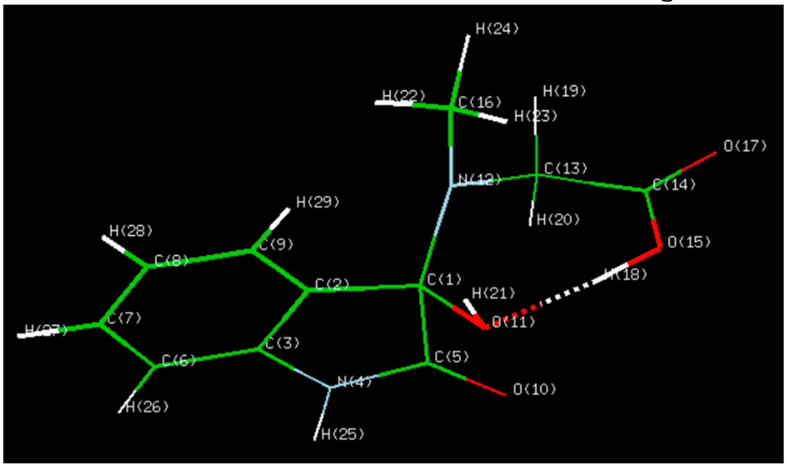

		x	y	z	q
1	C	-0.033304	-0.005219	0.317460	0.193133
2	C	1.448423	-0.306943	0.168510	-0.028395
3	C	2.093801	0.874460	-0.215682	0.096765
4	N	1.170610	1.931591	-0.256398	-0.403516
5	C	-0.059314	1.541784	0.162511	0.283913
6	C	3.454784	0.929722	-0.460979	-0.101794
7	C	4.185583	-0.249611	-0.289813	-0.092500
8	C	3.569238	-1.425687	0.129237	-0.109801
9	C	2.189952	-1.460275	0.366979	-0.085677
10	O	-1.019415	2.251906	0.397136	-0.366899
11	O	-0.520102	-0.245728	1.642145	-0.402329
12	N	-0.824866	-0.615696	-0.723630	-0.364443
13	C	-2.093218	-0.020325	-1.119879	-0.050842
14	C	-3.302606	-0.138928	-0.191203	0.253932
15	O	-3.114761	-0.301028	1.111124	-0.406765
16	C	-0.904430	-2.072717	-0.637985	-0.126798
17	O	-4.428750	-0.056390	-0.639860	-0.408942
18	H	-2.157545	-0.285308	1.370681	0.327475
19	H	-2.400936	-0.492670	-2.056188	0.127160
20	H	-1.977465	1.044733	-1.331770	0.126361
21	H	-0.222684	-1.118081	1.946114	0.377470
22	H	0.093812	-2.511153	-0.659869	0.104823
23	H	-1.430280	-2.420579	0.263284	0.097232
24	H	-1.446214	-2.441459	-1.511381	0.113618
25	H	1.419018	2.905753	-0.386486	0.372999
26	H	3.933231	1.856316	-0.759677	0.123512
27	H	5.255358	-0.239092	-0.473168	0.116167
28	H	4.160495	-2.322693	0.278707	0.112345
29	H	1.723765	-2.374684	0.720650	0.121849

TS6

log file: geo11_ts1.log
E(RM06-2X) = -836.497619215 A.U.
Zero-point correction= 0.310959
Thermal correction to Energy= 0.331562
Thermal correction to Enthalpy= 0.332506
Thermal correction to Gibbs Free Energy= 0.262067
Sum of electronic and zero-point Energies= -991.203362
Sum of electronic and thermal Energies= -991.182759
Sum of electronic and thermal Enthalpies= -991.181815
Sum of electronic and thermal Free Energies= -991.252255

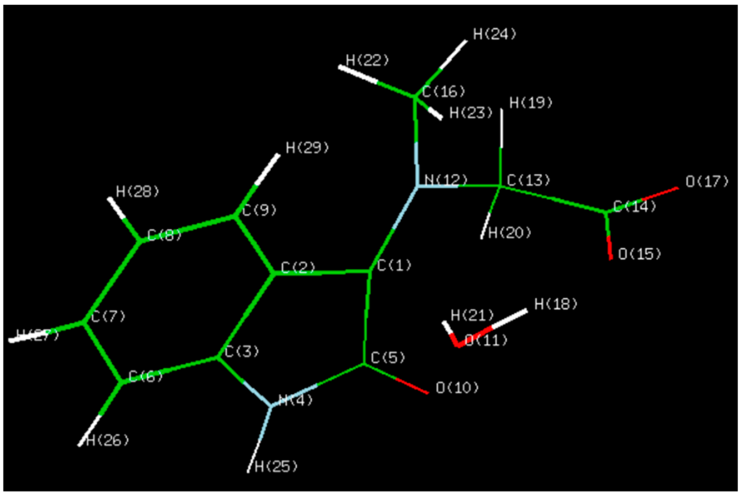

		x	y	z	q
1	C	-0.036148	0.051478	-0.145884	0.228356
2	C	1.395623	-0.333331	-0.059984	-0.023214
3	C	2.132868	0.859210	-0.142225	0.108195
4	N	1.272954	1.957242	-0.255163	-0.401160
5	C	-0.034285	1.596143	-0.226313	0.293334
6	C	3.515763	0.877281	-0.105548	-0.094756
7	C	4.169162	-0.348298	0.037669	-0.077966
8	C	3.456330	-1.540732	0.148919	-0.103786
9	C	2.060499	-1.543940	0.105532	-0.072739
10	O	-0.998769	2.332736	-0.224265	-0.346254
11	O	-0.470501	0.143657	1.706728	-0.526134
12	N	-0.976317	-0.659223	-0.748584	-0.281206
13	C	-2.304011	-0.119758	-1.053114	-0.047614
14	C	-3.297017	-0.192961	0.121359	0.212140
15	O	-2.835946	-0.346234	1.301873	-0.441859
16	C	-0.885352	-2.122162	-0.725417	-0.100519
17	O	-4.492015	-0.084533	-0.173681	-0.482075
18	H	-1.516161	-0.077936	1.578404	0.301995
19	H	-2.710615	-0.700381	-1.881778	0.129240
20	H	-2.226556	0.917795	-1.373126	0.125999
21	H	-0.061873	-0.630622	2.124891	0.361111
22	H	-0.032072	-2.458277	-1.317112	0.119168
23	H	-0.795805	-2.483379	0.303483	0.121149
24	H	-1.795374	-2.529692	-1.162567	0.129613
25	H	1.570438	2.926228	-0.248021	0.378031
26	H	4.063814	1.810053	-0.179529	0.128215
27	H	5.253871	-0.366958	0.070792	0.121554
28	H	3.986761	-2.477894	0.275891	0.116569
29	H	1.524364	-2.477650	0.219700	0.124602

21

log file: geo11_ts1_m2.log
E(RM06-2X) = -836.509750178 A.U.
Zero-point correction= 0.310959
Thermal correction to Energy= 0.331562
Thermal correction to Enthalpy= 0.332506
Thermal correction to Gibbs Free Energy= 0.262067
Sum of electronic and zero-point Energies= -991.203362
Sum of electronic and thermal Energies= -991.182759
Sum of electronic and thermal Enthalpies= -991.181815
Sum of electronic and thermal Free Energies= -991.252255

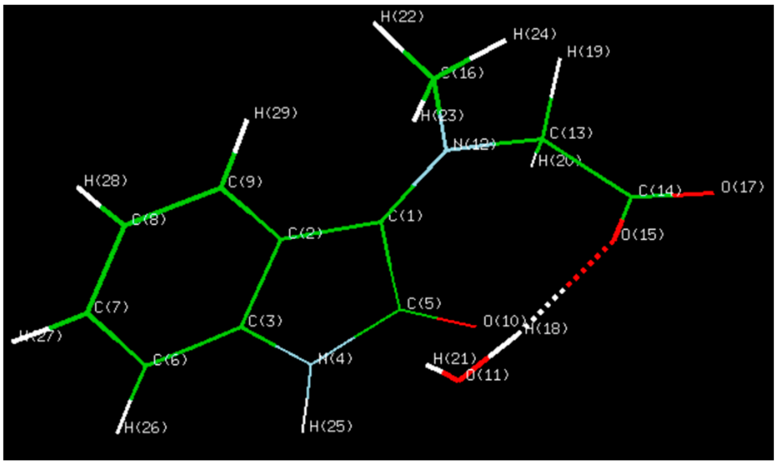

		x	y	z	q
1	C	-0.005062	-0.103606	-0.493048	0.220257
2	C	1.392913	-0.382362	-0.222997	-0.023273
3	C	2.039338	0.873107	-0.196279	0.122115
4	N	1.127248	1.900957	-0.423556	-0.403678
5	C	-0.139423	1.432430	-0.566735	0.300712
6	C	3.400899	0.995097	0.012997	-0.090016
7	C	4.129148	-0.181289	0.192473	-0.061298
8	C	3.516410	-1.436090	0.153383	-0.100014
9	C	2.146079	-1.550537	-0.057185	-0.060984
10	O	-1.155267	2.078553	-0.698126	-0.324112
11	O	-0.352558	0.774555	2.048549	-0.674128
12	N	-0.998850	-0.907414	-0.657939	-0.204950
13	C	-2.346220	-0.462938	-1.032252	-0.043834
14	C	-3.222728	-0.168749	0.214872	0.199958
15	O	-2.717449	-0.404782	1.342945	-0.459983
16	C	-0.866125	-2.336218	-0.349551	-0.085637
17	O	-4.361474	0.246641	-0.049798	-0.498988
18	H	-1.227602	0.370391	1.860709	0.289344
19	H	-2.800433	-1.278510	-1.597739	0.138094
20	H	-2.289329	0.420771	-1.661003	0.128294
21	H	0.148131	0.065488	2.471350	0.337495
22	H	-0.303143	-2.832102	-1.144170	0.136539
23	H	-0.361571	-2.450556	0.610585	0.133604
24	H	-1.865442	-2.760885	-0.286144	0.137771
25	H	1.340737	2.889390	-0.357822	0.380594
26	H	3.877174	1.968822	0.032539	0.131580
27	H	5.199468	-0.115887	0.360796	0.126687
28	H	4.112029	-2.331638	0.288308	0.119852
29	H	1.693818	-2.532900	-0.089581	0.128015

TS7

log file: geo11_ts2.log
E(RM06-2X) = -836.479439985 A.U.

Zero-point correction=	0.310959
Thermal correction to Energy=	0.331562
Thermal correction to Enthalpy=	0.332506
Thermal correction to Gibbs Free Energy=	0.262067
Sum of electronic and zero-point Energies=	-991.203362
Sum of electronic and thermal Energies=	-991.182759
Sum of electronic and thermal Enthalpies=	-991.181815
Sum of electronic and thermal Free Energies=	-991.252255

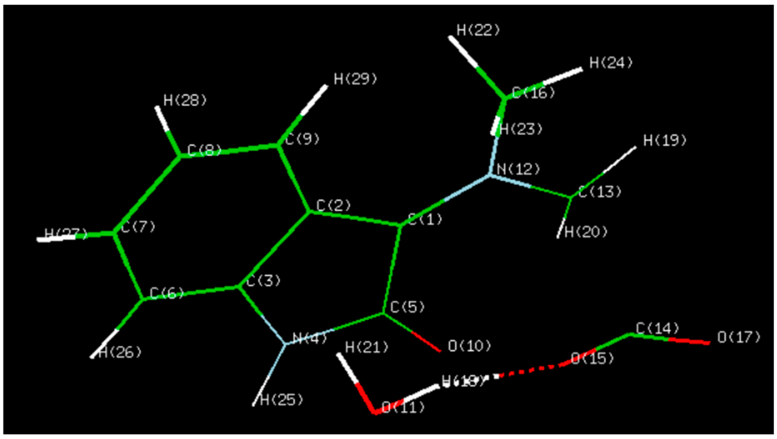

		x	y	z	q
1	C	0.162075	0.274870	-0.676248	0.098207
2	C	-1.238692	0.464450	-0.336133	-0.028181
3	C	-1.832025	-0.817019	-0.458565	0.098594
4	N	-0.870549	-1.739463	-0.846243	-0.415985
5	C	0.362792	-1.158557	-0.985717	0.253311
6	C	-3.173004	-1.049765	-0.201825	-0.104286
7	C	-3.955126	0.033551	0.197098	-0.097175
8	C	-3.398571	1.306956	0.327894	-0.115278
9	C	-2.048109	1.535092	0.065035	-0.092686
10	O	1.398248	-1.750252	-1.287802	-0.384340
11	O	-0.562683	-1.021096	2.398740	-0.702846
12	N	1.165429	1.154324	-0.699147	-0.211180
13	C	2.446944	0.842642	-0.979522	-0.062222
14	C	3.105845	-0.182759	0.745960	0.335649
15	O	2.129171	-0.423725	1.383096	-0.278665
16	C	0.894101	2.514480	-0.204483	-0.085723
17	O	4.282677	-0.230467	0.577348	-0.316030
18	H	0.265525	-0.692360	2.022431	0.300735
19	H	3.128720	1.680524	-1.028121	0.131751
20	H	2.622131	-0.033152	-1.586857	0.125433
21	H	-1.203849	-0.332733	2.181274	0.316894
22	H	0.165796	2.988699	-0.863263	0.134707
23	H	0.503059	2.447618	0.812258	0.131785
24	H	1.821320	3.081274	-0.211961	0.136766
25	H	-1.027096	-2.732259	-0.965494	0.369359
26	H	-3.589355	-2.046493	-0.303267	0.121916
27	H	-5.008453	-0.119344	0.409204	0.114145
28	H	-4.022446	2.137835	0.639886	0.110164
29	H	-1.656203	2.537043	0.179623	0.115185

22

log file: geo11_ts2_m2.log
E(RM06-2X) = -836.501207270 A.U.
Zero-point correction= 0.310959
Thermal correction to Energy= 0.331562
Thermal correction to Enthalpy= 0.332506
Thermal correction to Gibbs Free Energy= 0.262067
Sum of electronic and zero-point Energies= -991.203362
Sum of electronic and thermal Energies= -991.182759
Sum of electronic and thermal Enthalpies= -991.181815
Sum of electronic and thermal Free Energies= -991.252255

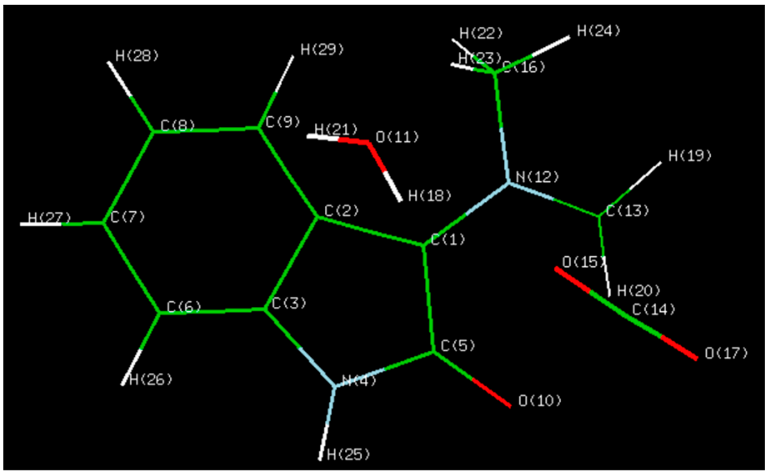

		x	y	z	q
1	C	0.104571	0.153895	-0.769084	0.015508
2	C	-1.302628	0.211059	-0.455181	-0.034640
3	C	-1.686753	-1.135827	-0.202060	0.080100
4	N	-0.575137	-1.945837	-0.346766	-0.427053
5	C	0.544145	-1.209846	-0.694119	0.207969
6	C	-2.982645	-1.497380	0.137289	-0.117370
7	C	-3.939144	-0.488618	0.243143	-0.125687
8	C	-3.590624	0.843247	0.003333	-0.125883
9	C	-2.289383	1.203623	-0.347878	-0.114444
10	O	1.684174	-1.699790	-0.842528	-0.440710
11	O	-0.378775	1.007988	2.419685	-0.688148
12	N	0.998961	1.195467	-0.972647	-0.210158
13	C	2.183566	1.052298	-1.484808	0.020818
14	C	3.182287	-0.409650	0.959032	0.425113
15	O	2.245105	-0.036484	1.541190	-0.213626
16	C	0.585409	2.531670	-0.504284	-0.092469
17	O	4.162064	-0.749097	0.433453	-0.233768
18	H	0.117083	0.381662	1.874391	0.302329
19	H	2.838108	1.913533	-1.513688	0.150258
20	H	2.478468	0.086192	-1.869137	0.137915
21	H	-1.263838	0.988139	2.030710	0.314889
22	H	-0.158007	2.930249	-1.194816	0.131567
23	H	0.167682	2.424648	0.497689	0.116101
24	H	1.457197	3.182723	-0.490730	0.134107
25	H	-0.554786	-2.948320	-0.216772	0.359321
26	H	-3.232236	-2.537906	0.321163	0.113892
27	H	-4.958999	-0.742348	0.513806	0.104227
28	H	-4.345694	1.618772	0.087632	0.103216
29	H	-2.065096	2.247491	-0.531056	0.106606

24

log file: geo5.log
E(RM06-2X) = -836.516051237 A.U.
Zero-point correction= 0.310959
Thermal correction to Energy= 0.331562
Thermal correction to Enthalpy= 0.332506
Thermal correction to Gibbs Free Energy= 0.262067
Sum of electronic and zero-point Energies= -991.203362
Sum of electronic and thermal Energies= -991.182759
Sum of electronic and thermal Enthalpies= -991.181815
Sum of electronic and thermal Free Energies= -991.252255

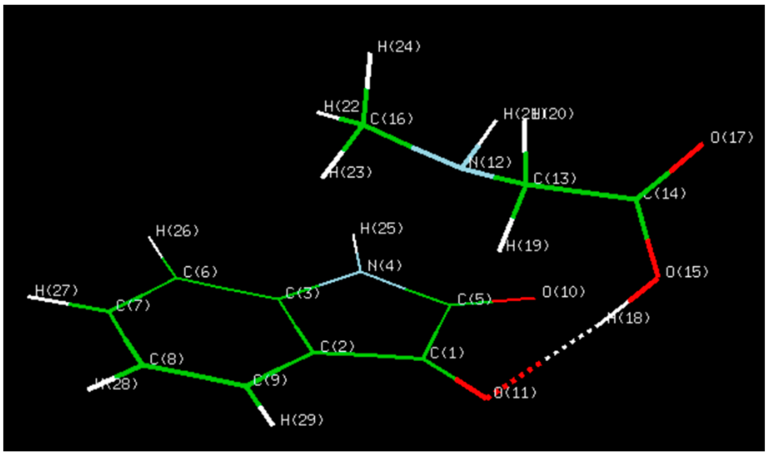

		x	y	z	q
1	C	0.168515	0.667745	0.944455	0.199140
2	C	1.491485	0.095942	0.721280	-0.037799
3	C	2.002915	0.694722	-0.441825	0.118100
4	N	1.091900	1.627055	-0.960323	-0.407495
5	C	-0.035798	1.699396	-0.195629	0.275084
6	C	3.241601	0.333767	-0.946218	-0.094698
7	C	3.951004	-0.649258	-0.250752	-0.069472
8	C	3.448812	-1.256246	0.905516	-0.103372
9	C	2.202429	-0.884422	1.403272	-0.061685
10	O	-0.995250	2.430619	-0.325075	-0.361042
11	O	-0.644017	0.477676	1.833288	-0.298955
12	N	-1.086136	-0.715108	-0.929028	-0.484415
13	C	-2.104332	-1.403711	-0.137417	-0.047570
14	C	-3.329150	-0.531310	0.077604	0.267434
15	O	-3.160888	0.623622	0.715728	-0.402991
16	C	-0.105170	-1.658656	-1.459342	-0.129183
17	O	-4.430072	-0.845417	-0.322082	-0.393992
18	H	-2.254926	0.723648	1.083615	0.326475
19	H	-1.675269	-1.660424	0.841489	0.118699
20	H	-2.456183	-2.327735	-0.612806	0.112523
21	H	-1.532546	-0.235229	-1.709831	0.318115
22	H	0.631495	-1.117005	-2.058641	0.099589
23	H	0.416380	-2.146126	-0.629451	0.096180
24	H	-0.562355	-2.439089	-2.083974	0.094033
25	H	1.256893	2.195337	-1.782394	0.373466
26	H	3.640858	0.791656	-1.844559	0.127128
27	H	4.924235	-0.951156	-0.625562	0.122379
28	H	4.033487	-2.016711	1.410960	0.116443
29	H	1.785535	-1.340614	2.296065	0.127857

TS17

log file: geo4_ts1.log
E(RM06-2X) = -836.511418675 A.U.
Zero-point correction= 0.310959
Thermal correction to Energy= 0.331562
Thermal correction to Enthalpy= 0.332506
Thermal correction to Gibbs Free Energy= 0.262067
Sum of electronic and zero-point Energies= -991.203362
Sum of electronic and thermal Energies= -991.182759
Sum of electronic and thermal Enthalpies= -991.181815
Sum of electronic and thermal Free Energies= -991.252255

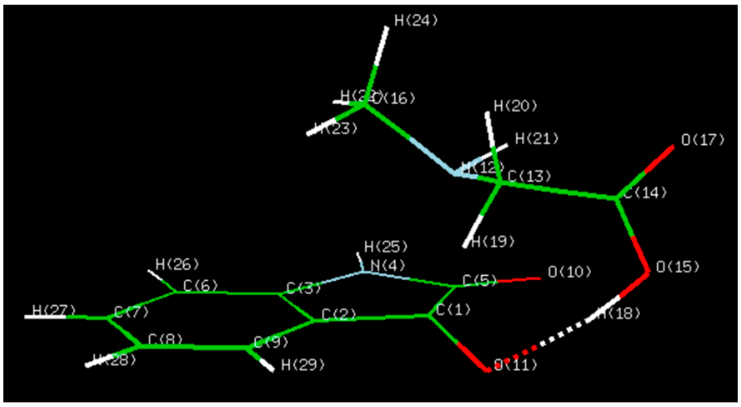

		x	y	z	q
1	C	0.038997	0.396188	-0.687321	0.183463
2	C	-1.310073	-0.205468	-0.535054	-0.036004
3	C	-2.099567	0.705611	0.176058	0.103731
4	N	-1.349919	1.857197	0.484567	-0.405275
5	C	-0.091477	1.796451	-0.032774	0.270624
6	C	-3.421472	0.432391	0.484085	-0.098565
7	C	-3.935982	-0.794134	0.050747	-0.084356
8	C	-3.161022	-1.706191	-0.667551	-0.106493
9	C	-1.829026	-1.412209	-0.971641	-0.074853
10	O	0.783174	2.640884	0.016477	-0.376500
11	O	0.811255	0.188689	-1.658218	-0.391384
12	N	0.998694	-0.317580	0.898263	-0.396590
13	C	1.993452	-1.274061	0.378625	-0.038520
14	C	3.284358	-0.585243	-0.049846	0.261978
15	O	3.168166	0.454885	-0.853247	-0.408900
16	C	0.142191	-0.908684	1.937073	-0.113465
17	O	4.366449	-0.972546	0.342968	-0.400276
18	H	2.222809	0.505953	-1.243588	0.314792
19	H	1.560572	-1.790650	-0.486709	0.132206
20	H	2.248283	-2.008394	1.144825	0.132288
21	H	1.481076	0.491874	1.296170	0.339454
22	H	-0.582517	-0.164214	2.272151	0.112329
23	H	-0.384319	-1.769694	1.521117	0.111789
24	H	0.747393	-1.229436	2.789794	0.117760
25	H	-1.720221	2.669782	0.964153	0.371897
26	H	-4.031737	1.140909	1.033727	0.124568
27	H	-4.969643	-1.036631	0.277159	0.118002
28	H	-3.597342	-2.644366	-0.992918	0.113604
29	H	-1.210411	-2.105657	-1.533945	0.122684

42

```
log file: geo4.log
E(RM06-2X) =  -836.523886866     A.U.
Zero-point correction=                             0.310959
Thermal correction to Energy=                      0.331562
Thermal correction to Enthalpy=                    0.332506
Thermal correction to Gibbs Free Energy=           0.262067
Sum of electronic and zero-point Energies=             -991.203362
Sum of electronic and thermal Energies=                -991.182759
Sum of electronic and thermal Enthalpies=              -991.181815
Sum of electronic and thermal Free Energies=           -991.252255
```

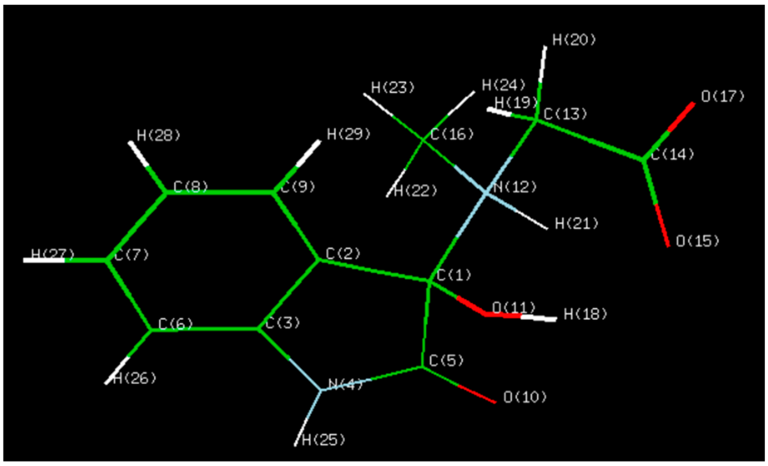

		x	y	z	q
1	C	0.127694	0.349418	-0.298983	0.198011
2	C	-1.186138	-0.377992	-0.217659	-0.030382
3	C	-2.198675	0.580486	-0.099823	0.102697
4	N	-1.644013	1.874261	-0.063988	-0.396979
5	C	-0.293733	1.842681	-0.139552	0.278934
6	C	-3.536028	0.228201	-0.043040	-0.095890
7	C	-3.840652	-1.133998	-0.112719	-0.082864
8	C	-2.843474	-2.099620	-0.243757	-0.103371
9	C	-1.498400	-1.724383	-0.306168	-0.075681
10	O	0.500110	2.762861	-0.099996	-0.362623
11	O	0.770699	0.130413	-1.495475	-0.420155
12	N	1.090706	0.084183	0.855725	-0.340068
13	C	1.996820	-1.085251	0.621542	-0.042720
14	C	3.234906	-0.644546	-0.188636	0.202684
15	O	3.249680	0.564439	-0.565183	-0.465888
16	C	0.414186	0.044651	2.182513	-0.093659
17	O	4.090055	-1.520116	-0.375820	-0.490424
18	H	1.649426	0.577261	-1.454880	0.331323
19	H	1.452574	-1.874416	0.102124	0.137127
20	H	2.315897	-1.452877	1.598102	0.140327
21	H	1.728784	0.900837	0.827051	0.346996
22	H	-0.245426	0.908488	2.280959	0.128726
23	H	-0.154478	-0.880852	2.260156	0.127411
24	H	1.187928	0.084732	2.948029	0.139696
25	H	-2.180970	2.731273	0.011654	0.377875
26	H	-4.312072	0.980326	0.047706	0.126936
27	H	-4.880380	-1.442433	-0.069114	0.119722
28	H	-3.112116	-3.148586	-0.303984	0.115725
29	H	-0.719395	-2.471517	-0.420467	0.126496

TS18

log file: geo4_ts2.log
E(RM06-2X) = -836.520045810 A.U.

Zero-point correction=	0.310959
Thermal correction to Energy=	0.331562
Thermal correction to Enthalpy=	0.332506
Thermal correction to Gibbs Free Energy=	0.262067
Sum of electronic and zero-point Energies=	-991.203362
Sum of electronic and thermal Energies=	-991.182759
Sum of electronic and thermal Enthalpies=	-991.181815
Sum of electronic and thermal Free Energies=	-991.252255

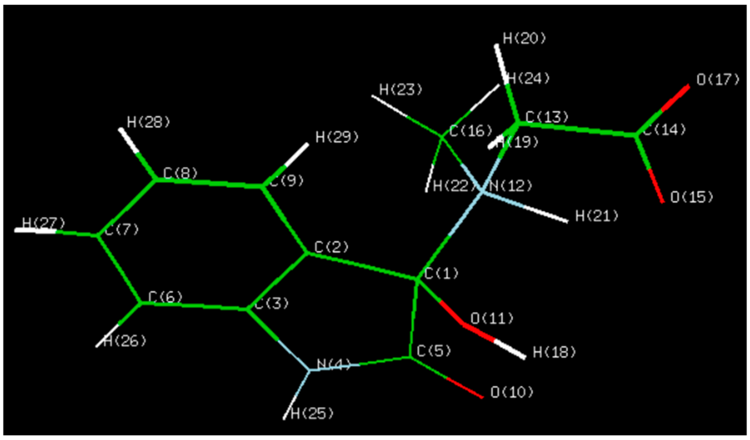

		x	y	z	q
1	C	0.060447	0.383617	-0.438434	0.196783
2	C	-1.232226	-0.372079	-0.271393	-0.030678
3	C	-2.237943	0.554133	0.024357	0.100107
4	N	-1.701061	1.855158	0.064644	-0.399023
5	C	-0.368751	1.856355	-0.176687	0.279391
6	C	-3.550748	0.168006	0.233026	-0.098229
7	C	-3.837656	-1.196729	0.135615	-0.086633
8	C	-2.846607	-2.131225	-0.160503	-0.105726
9	C	-1.525807	-1.721365	-0.369639	-0.080489
10	O	0.397881	2.799609	-0.223325	-0.368049
11	O	0.568601	0.261060	-1.725574	-0.408950
12	N	1.123993	0.042065	0.543866	-0.343245
13	C	1.872697	-1.201395	0.231514	-0.047960
14	C	3.315585	-0.706820	-0.014887	0.220681
15	O	3.390289	0.574370	0.058381	-0.432957
16	C	0.710301	0.139622	1.959417	-0.106470
17	O	4.219451	-1.502648	-0.250445	-0.460967
18	H	1.237356	0.954649	-1.862652	0.371242
19	H	1.493493	-1.678120	-0.672536	0.134371
20	H	1.834856	-1.904253	1.064857	0.136609
21	H	2.109658	0.717551	0.348459	0.306118
22	H	0.287569	1.127304	2.153729	0.121150
23	H	-0.019351	-0.636907	2.198102	0.115094
24	H	1.602747	0.014270	2.573916	0.130114
25	H	-2.239548	2.697359	0.233552	0.376109
26	H	-4.321982	0.895704	0.460637	0.125474
27	H	-4.857968	-1.531621	0.293805	0.118178
28	H	-3.100959	-3.183158	-0.232041	0.114310
29	H	-0.751938	-2.445194	-0.605996	0.123611

43

```
log file: geo6.log
E(RM06-2X)  =   -836.530895889     A.U.
Zero-point correction=                              0.310959
Thermal correction to Energy=                       0.331562
Thermal correction to Enthalpy=                     0.332506
Thermal correction to Gibbs Free Energy=            0.262067
Sum of electronic and zero-point Energies=             -991.203362
Sum of electronic and thermal Energies=                -991.182759
Sum of electronic and thermal Enthalpies=              -991.181815
Sum of electronic and thermal Free Energies=           -991.252255
```

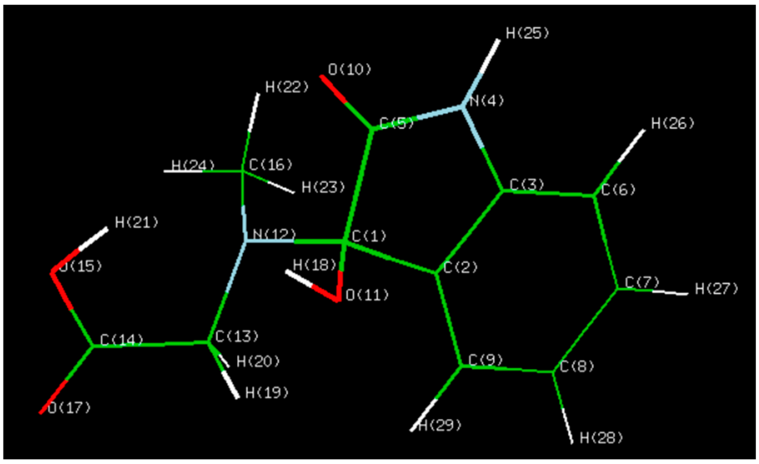

		x	y	z	q
1	C	0.028161	0.540951	0.539260	0.188265
2	C	1.223776	-0.362997	0.319551	-0.032710
3	C	2.272365	0.410288	-0.189391	0.095732
4	N	1.871423	1.755309	-0.289381	-0.405290
5	C	0.593130	1.933459	0.129083	0.274133
6	C	3.502228	-0.138116	-0.512112	-0.102970
7	C	3.663787	-1.511569	-0.305138	-0.092836
8	C	2.632287	-2.295540	0.208091	-0.110455
9	C	1.396287	-1.721038	0.526508	-0.087020
10	O	-0.027795	2.977552	0.211236	-0.384350
11	O	-0.319252	0.562499	1.893667	-0.425464
12	N	-1.153016	0.270121	-0.276666	-0.352809
13	C	-1.819814	-0.980510	0.063749	-0.044684
14	C	-3.325000	-0.831407	-0.093341	0.264486
15	O	-3.766153	0.423540	-0.113097	-0.391999
16	C	-0.919222	0.376182	-1.719218	-0.125703
17	O	-4.083491	-1.772740	-0.160296	-0.396561
18	H	-0.983653	1.262715	2.008286	0.364367
19	H	-1.644476	-1.215632	1.118489	0.135872
20	H	-1.493473	-1.835076	-0.542076	0.121201
21	H	-2.973077	1.002653	-0.058425	0.350280
22	H	-0.565437	1.378006	-1.974124	0.108416
23	H	-0.197245	-0.369457	-2.080978	0.094802
24	H	-1.868760	0.219642	-2.237851	0.114935
25	H	2.474690	2.516752	-0.578047	0.371534
26	H	4.306665	0.474915	-0.904410	0.122541
27	H	4.616747	-1.971832	-0.546878	0.115510
28	H	2.788841	-3.357369	0.365665	0.111681
29	H	0.595956	-2.328341	0.938346	0.119078

TS19

log file: geo6_ts1.log
E(RM06-2X) = -836.525072273 A.U.

Zero-point correction=	0.310959
Thermal correction to Energy=	0.331562
Thermal correction to Enthalpy=	0.332506
Thermal correction to Gibbs Free Energy=	0.262067
Sum of electronic and zero-point Energies=	-991.203362
Sum of electronic and thermal Energies=	-991.182759
Sum of electronic and thermal Enthalpies=	-991.181815
Sum of electronic and thermal Free Energies=	-991.252255

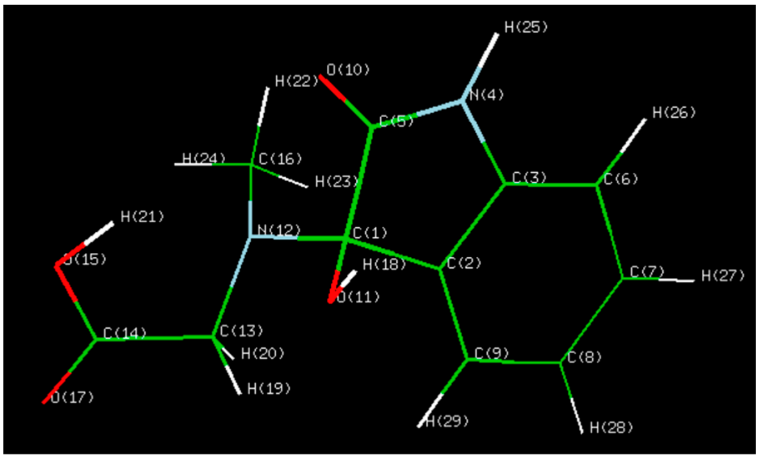

		x	y	z	q
1	C	0.014453	0.539758	0.556870	0.190512
2	C	1.222717	-0.355455	0.340918	-0.035796
3	C	2.252223	0.415988	-0.206127	0.093785
4	N	1.831928	1.754574	-0.325247	-0.405190
5	C	0.567926	1.933358	0.132708	0.277144
6	C	3.482009	-0.123972	-0.542594	-0.103309
7	C	3.664075	-1.490687	-0.308866	-0.094258
8	C	2.652307	-2.273937	0.243570	-0.110764
9	C	1.416263	-1.706269	0.574469	-0.088436
10	O	-0.052675	2.976266	0.223055	-0.381344
11	O	-0.444203	0.553935	1.882728	-0.427729
12	N	-1.137541	0.244056	-0.292542	-0.350021
13	C	-1.819799	-0.996010	0.062161	-0.044622
14	C	-3.322189	-0.821504	-0.104950	0.263765
15	O	-3.733508	0.442264	-0.134751	-0.392719
16	C	-0.860510	0.307168	-1.728970	-0.126037
17	O	-4.098551	-1.748626	-0.170083	-0.398102
18	H	-0.102124	1.331326	2.346098	0.371970
19	H	-1.658159	-1.226086	1.119733	0.136695
20	H	-1.501817	-1.860960	-0.532607	0.121343
21	H	-2.920619	0.996676	-0.077668	0.347005
22	H	-0.506684	1.303772	-2.004524	0.107971
23	H	-0.124288	-0.443195	-2.049858	0.093923
24	H	-1.794408	0.128382	-2.268165	0.115230
25	H	2.419053	2.517160	-0.642703	0.372049
26	H	4.270469	0.489594	-0.965447	0.122275
27	H	4.617013	-1.945647	-0.560468	0.115037
28	H	2.824727	-3.330172	0.421346	0.111310
29	H	0.630779	-2.312597	1.016051	0.118276

44

log file: geo8.log
E(RM06-2X) = -836.528034336 A.U.
Zero-point correction= 0.310959
Thermal correction to Energy= 0.331562
Thermal correction to Enthalpy= 0.332506
Thermal correction to Gibbs Free Energy= 0.262067
Sum of electronic and zero-point Energies= -991.203362
Sum of electronic and thermal Energies= -991.182759
Sum of electronic and thermal Enthalpies= -991.181815
Sum of electronic and thermal Free Energies= -991.252255

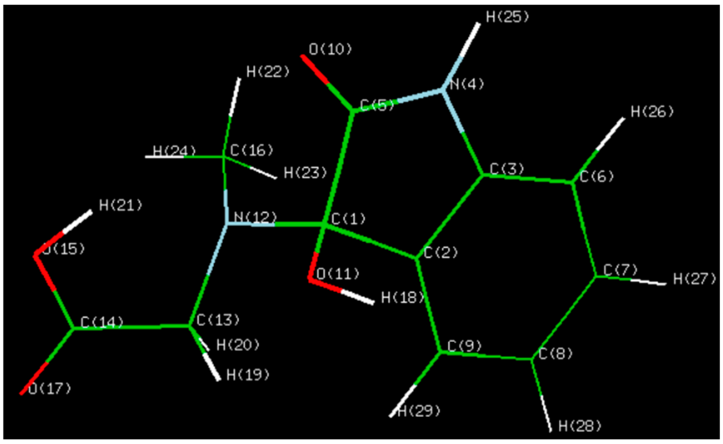

		x	y	z	q
1	C	0.018504	0.539331	0.548826	0.190921
2	C	1.217396	-0.362684	0.316649	-0.033774
3	C	2.262861	0.414001	-0.194225	0.095969
4	N	1.861470	1.758405	-0.284434	-0.407939
5	C	0.584092	1.937912	0.143816	0.271508
6	C	3.491327	-0.132788	-0.525833	-0.103196
7	C	3.655339	-1.506829	-0.324795	-0.092191
8	C	2.628153	-2.294034	0.192713	-0.110795
9	C	1.393900	-1.721514	0.520382	-0.085723
10	O	-0.029795	2.985068	0.221330	-0.387715
11	O	-0.438353	0.573255	1.874433	-0.425020
12	N	-1.152662	0.274140	-0.275093	-0.348576
13	C	-1.816898	-0.985946	0.036698	-0.045200
14	C	-3.323092	-0.823950	-0.100672	0.264008
15	O	-3.753770	0.433649	-0.072277	-0.392016
16	C	-0.916847	0.410435	-1.713907	-0.125352
17	O	-4.087960	-1.758244	-0.193579	-0.397977
18	H	0.314262	0.430557	2.466551	0.370150
19	H	-1.633235	-1.252509	1.081909	0.135380
20	H	-1.498807	-1.821960	-0.598342	0.120792
21	H	-2.952089	1.000874	-0.000953	0.348744
22	H	-0.582421	1.422822	-1.951535	0.108359
23	H	-0.181139	-0.315556	-2.088276	0.094365
24	H	-1.862941	0.244939	-2.235906	0.115338
25	H	2.461533	2.519305	-0.580667	0.370427
26	H	4.293063	0.482191	-0.920712	0.122483
27	H	4.607225	-1.965259	-0.574205	0.115742
28	H	2.787091	-3.356142	0.345885	0.111756
29	H	0.596405	-2.330515	0.935765	0.119533

TS20

log file: geo8_ts5c.log
E(RM06-2X) = -836.527368482 A.U.
Zero-point correction= 0.310959
Thermal correction to Energy= 0.331562
Thermal correction to Enthalpy= 0.332506
Thermal correction to Gibbs Free Energy= 0.262067
Sum of electronic and zero-point Energies= -991.203362
Sum of electronic and thermal Energies= -991.182759
Sum of electronic and thermal Enthalpies= -991.181815
Sum of electronic and thermal Free Energies= -991.252255

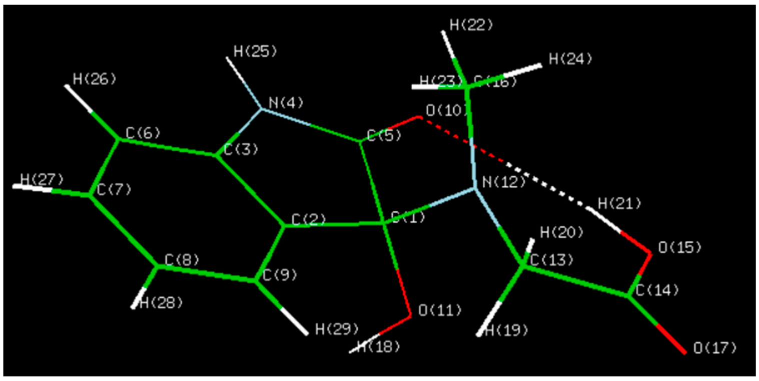

		x	y	z	q
1	C	0.067851	0.389054	-0.395677	0.192367
2	C	-1.232828	-0.382684	-0.253367	-0.033740
3	C	-2.260077	0.530929	0.007133	0.095841
4	N	-1.747259	1.840605	0.030825	-0.407527
5	C	-0.406190	1.862649	-0.182024	0.271274
6	C	-3.571599	0.129464	0.199019	-0.103187
7	C	-3.839585	-1.240465	0.121589	-0.091979
8	C	-2.829427	-2.165028	-0.138309	-0.110772
9	C	-1.510798	-1.737817	-0.329580	-0.085367
10	O	0.324320	2.834562	-0.225607	-0.385478
11	O	0.685125	0.274086	-1.651225	-0.419532
12	N	1.110503	0.113564	0.577562	-0.345441
13	C	1.876174	-1.104088	0.325220	-0.043501
14	C	3.318449	-0.752196	-0.020009	0.259290
15	O	3.558384	0.546751	-0.135682	-0.396197
16	C	0.701367	0.271588	1.971588	-0.122178
17	O	4.180947	-1.587596	-0.189519	-0.406109
18	H	0.001577	0.191918	-2.332107	0.372159
19	H	1.485600	-1.672897	-0.522285	0.132522
20	H	1.885842	-1.766819	1.195564	0.132521
21	H	2.694231	0.990520	0.059704	0.336017
22	H	0.274831	1.265952	2.128939	0.108228
23	H	-0.031446	-0.485198	2.283258	0.094005
24	H	1.587639	0.187080	2.603710	0.115737
25	H	-2.304770	2.674202	0.176113	0.370635
26	H	-4.356877	0.850679	0.398996	0.122540
27	H	-4.858259	-1.586588	0.266296	0.115826
28	H	-3.067011	-3.221935	-0.195837	0.111737
29	H	-0.724296	-2.456955	-0.538285	0.120290

45

```
log file: geo8_ts5b_rmin.log
E(RM06-2X) =  -836.528364168     A.U.
Zero-point correction=                          0.310959
Thermal correction to Energy=                   0.331562
Thermal correction to Enthalpy=                 0.332506
Thermal correction to Gibbs Free Energy=        0.262067
Sum of electronic and zero-point Energies=          -991.203362
Sum of electronic and thermal Energies=             -991.182759
Sum of electronic and thermal Enthalpies=           -991.181815
Sum of electronic and thermal Free Energies=        -991.252255
```

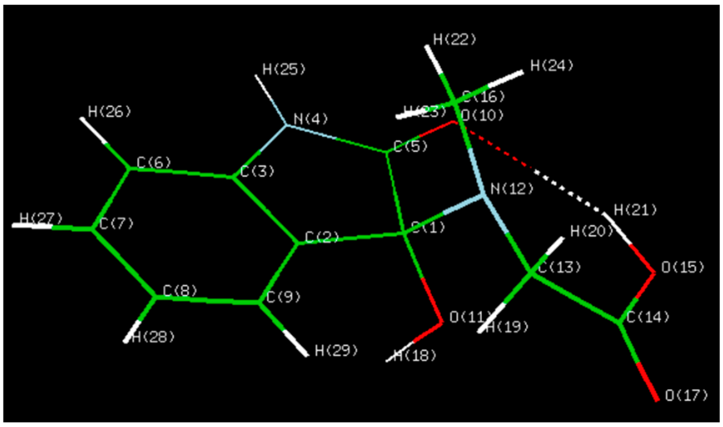

		x	y	z	q
1	C	0.150303	0.341618	-0.268597	0.191860
2	C	-1.182679	-0.385033	-0.194537	-0.032863
3	C	-2.200167	0.572772	-0.112958	0.096143
4	N	-1.650673	1.867206	-0.136779	-0.407786
5	C	-0.295907	1.838989	-0.223940	0.271010
6	C	-3.537847	0.222974	-0.034419	-0.103154
7	C	-3.845128	-1.140675	-0.042748	-0.091679
8	C	-2.846761	-2.108992	-0.131817	-0.110744
9	C	-1.501150	-1.733692	-0.212179	-0.084542
10	O	0.465547	2.786359	-0.275869	-0.384293
11	O	0.872656	0.099797	-1.447999	-0.411896
12	N	1.093556	0.135809	0.822750	-0.349142
13	C	1.913111	-1.060518	0.673181	-0.043157
14	C	3.170607	-0.762585	-0.127145	0.255439
15	O	3.473179	0.525369	-0.250733	-0.398720
16	C	0.490092	0.282950	2.146076	-0.123359
17	O	3.886858	-1.629736	-0.579276	-0.404502
18	H	0.249462	-0.029639	-2.177750	0.373782
19	H	1.408265	-1.911644	0.205821	0.123019
20	H	2.248457	-1.380583	1.664942	0.139953
21	H	2.722132	1.028052	0.138592	0.339353
22	H	-0.041022	1.237387	2.207848	0.107568
23	H	-0.213482	-0.526081	2.384179	0.092541
24	H	1.284662	0.294350	2.894334	0.114149
25	H	-2.193362	2.722934	-0.119603	0.370481
26	H	-4.313488	0.979026	0.027094	0.122532
27	H	-4.884945	-1.446680	0.016766	0.115934
28	H	-3.113762	-3.160330	-0.143868	0.111776
29	H	-0.725858	-2.489057	-0.294642	0.120280

TS21

log file: geo8_ts5b.log
E(RM06-2X) = -836.522480179 A.U.
Zero-point correction= 0.310959
Thermal correction to Energy= 0.331562
Thermal correction to Enthalpy= 0.332506
Thermal correction to Gibbs Free Energy= 0.262067
Sum of electronic and zero-point Energies= -991.203362
Sum of electronic and thermal Energies= -991.182759
Sum of electronic and thermal Enthalpies= -991.181815
Sum of electronic and thermal Free Energies= -991.252255

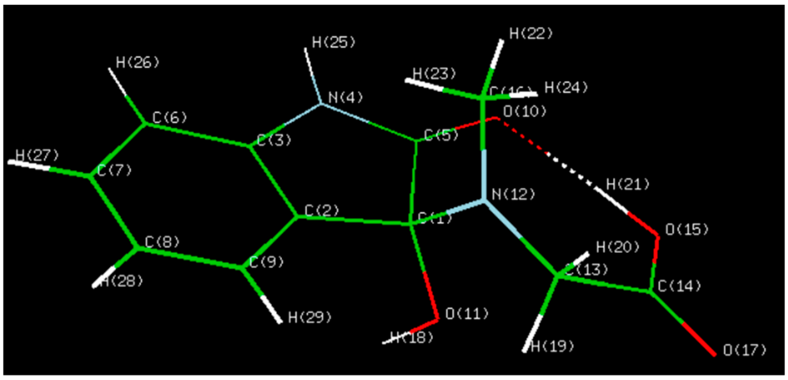

		x	y	z	q
1	C	0.075734	0.038190	-0.272146	0.198792
2	C	-1.376409	-0.400693	-0.181836	-0.033510
3	C	-2.182840	0.735395	-0.078658	0.095021
4	N	-1.379283	1.897339	-0.056991	-0.400681
5	C	-0.066323	1.589977	-0.113215	0.276145
6	C	-3.562582	0.652241	-0.002843	-0.102000
7	C	-4.126301	-0.627745	-0.034669	-0.091997
8	C	-3.334906	-1.771285	-0.135931	-0.109164
9	C	-1.941874	-1.662235	-0.210649	-0.084904
10	O	0.891786	2.349644	-0.057030	-0.357268
11	O	0.672745	-0.235100	-1.514185	-0.409957
12	N	0.970130	-0.454629	0.733066	-0.362689
13	C	2.099995	-1.268341	0.385324	-0.039618
14	C	3.316383	-0.521173	-0.157083	0.253415
15	O	3.264107	0.802820	-0.232711	-0.402979
16	C	0.670946	-0.106774	2.106702	-0.121150
17	O	4.317332	-1.117759	-0.500044	-0.412258
18	H	0.026428	-0.069714	-2.216464	0.374607
19	H	1.856963	-2.031569	-0.359814	0.121815
20	H	2.442575	-1.799360	1.278077	0.136059
21	H	2.385892	1.164576	0.021963	0.324572
22	H	0.774699	0.970893	2.296042	0.092590
23	H	-0.348710	-0.406241	2.383455	0.093063
24	H	1.364887	-0.628895	2.766500	0.118365
25	H	-1.736692	2.841040	0.040520	0.373138
26	H	-4.178393	1.541580	0.079505	0.123034
27	H	-5.205631	-0.727932	0.023477	0.115761
28	H	-3.802737	-2.749878	-0.156225	0.111903
29	H	-1.313907	-2.545646	-0.287776	0.119884

46

log file: geo8_ts5b_fmin.log

E(RM06-2X) = -836.524977954 A.U.

Zero-point correction= 0.310959

Thermal correction to Energy= 0.331562

Thermal correction to Enthalpy= 0.332506

Thermal correction to Gibbs Free Energy= 0.262067

Sum of electronic and zero-point Energies= -991.203362

Sum of electronic and thermal Energies= -991.182759

Sum of electronic and thermal Enthalpies= -991.181815

Sum of electronic and thermal Free Energies= -991.252255

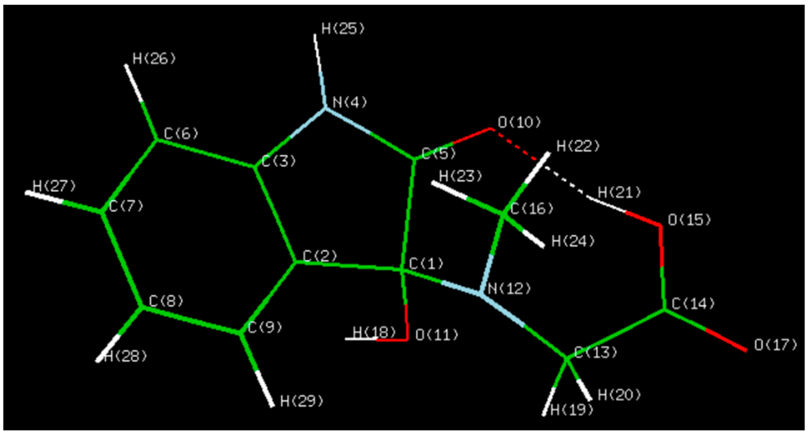

		x	y	z	q
1	C	-0.014935	-0.060262	-0.474373	0.197744
2	C	-1.477452	-0.383299	-0.220064	-0.031203
3	C	-2.158540	0.793105	0.094155	0.096026
4	N	-1.251424	1.876127	0.113251	-0.396392
5	C	0.003429	1.474828	-0.160674	0.281617
6	C	-3.518252	0.810707	0.350063	-0.100052
7	C	-4.193553	-0.412502	0.276971	-0.090947
8	C	-3.527008	-1.596491	-0.036594	-0.107464
9	C	-2.150531	-1.588633	-0.288053	-0.084205
10	O	1.011922	2.177550	-0.177312	-0.344388
11	O	0.372186	-0.245950	-1.809217	-0.412200
12	N	0.893886	-0.816590	0.356036	-0.362038
13	C	2.172043	-1.271152	-0.142383	-0.047713
14	C	3.408991	-0.389299	0.039292	0.253002
15	O	3.289844	0.927943	0.027854	-0.407984
16	C	0.864924	-0.476313	1.769026	-0.126707
17	O	4.507245	-0.899429	0.155550	-0.414252
18	H	-0.354067	0.031032	-2.386847	0.374478
19	H	2.098595	-1.487744	-1.210133	0.129939
20	H	2.424858	-2.210843	0.359091	0.119053
21	H	2.365893	1.274895	-0.117813	0.319210
22	H	1.396667	0.459584	2.001210	0.090693
23	H	-0.171635	-0.378800	2.106773	0.104449
24	H	1.329123	-1.285451	2.339051	0.111676
25	H	-1.498017	2.832684	0.343769	0.375373
26	H	-4.035504	1.731196	0.598874	0.124016
27	H	-5.260987	-0.435580	0.472726	0.116334
28	H	-4.079630	-2.528880	-0.082914	0.112576
29	H	-1.616810	-2.503886	-0.527980	0.119353

TS22

log file: geo8_ts9.log
E(RM06-2X) = -836.518887862 A.U.
Zero-point correction= 0.310959
Thermal correction to Energy= 0.331562
Thermal correction to Enthalpy= 0.332506
Thermal correction to Gibbs Free Energy= 0.262067
Sum of electronic and zero-point Energies= -991.203362
Sum of electronic and thermal Energies= -991.182759
Sum of electronic and thermal Enthalpies= -991.181815
Sum of electronic and thermal Free Energies= -991.252255

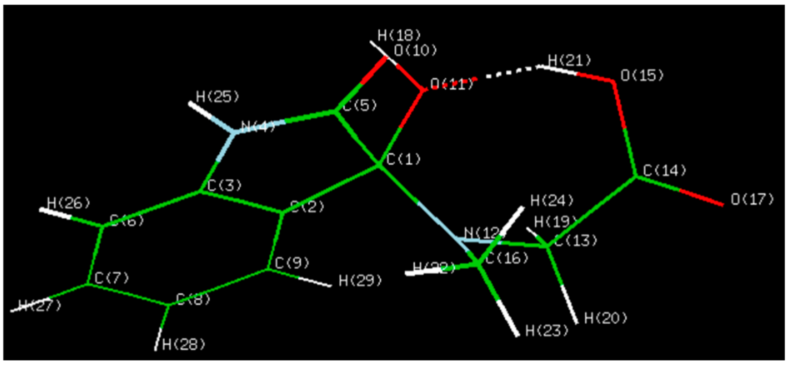

		x	y	z	q
1	C	-0.027027	0.154199	0.272084	0.199492
2	C	1.379645	-0.408033	0.173661	-0.035703
3	C	2.264850	0.646005	-0.062448	0.093393
4	N	1.558943	1.865458	-0.096864	-0.405582
5	C	0.232289	1.688860	0.126084	0.276709
6	C	3.624718	0.441050	-0.224552	-0.103931
7	C	4.086024	-0.876683	-0.141196	-0.094594
8	C	3.214317	-1.939751	0.091925	-0.111066
9	C	1.843203	-1.708789	0.248500	-0.088808
10	O	-0.622522	2.545866	0.254201	-0.375337
11	O	-0.623728	-0.060809	1.539004	-0.390498
12	N	-0.934955	-0.342902	-0.739735	-0.364337
13	C	-1.943296	-1.306221	-0.356055	-0.043894
14	C	-3.275233	-0.699536	0.095429	0.257520
15	O	-3.224728	0.368408	0.891706	-0.403650
16	C	-1.339610	0.534356	-1.830055	-0.127247
17	O	-4.345694	-1.152980	-0.248889	-0.397875
18	H	-0.013061	0.206585	2.243740	0.382766
19	H	-1.576491	-1.946361	0.450886	0.132587
20	H	-2.180685	-1.945212	-1.211255	0.121571
21	H	-2.306091	0.526212	1.197161	0.335901
22	H	-0.476188	1.080357	-2.218749	0.108267
23	H	-1.722321	-0.088880	-2.643261	0.108751
24	H	-2.117434	1.258656	-1.549718	0.087619
25	H	1.991634	2.778940	-0.170686	0.371187
26	H	4.301973	1.268659	-0.406992	0.122004
27	H	5.147163	-1.071164	-0.261984	0.114828
28	H	3.601878	-2.951324	0.151222	0.111097
29	H	1.155801	-2.530979	0.427386	0.118850

47

log file: geo8_ts5_m1.log
E(RM06-2X) = -836.528165593 A.U.
Zero-point correction= 0.310959
Thermal correction to Energy= 0.331562
Thermal correction to Enthalpy= 0.332506
Thermal correction to Gibbs Free Energy= 0.262067
Sum of electronic and zero-point Energies= -991.203362
Sum of electronic and thermal Energies= -991.182759
Sum of electronic and thermal Enthalpies= -991.181815
Sum of electronic and thermal Free Energies= -991.252255

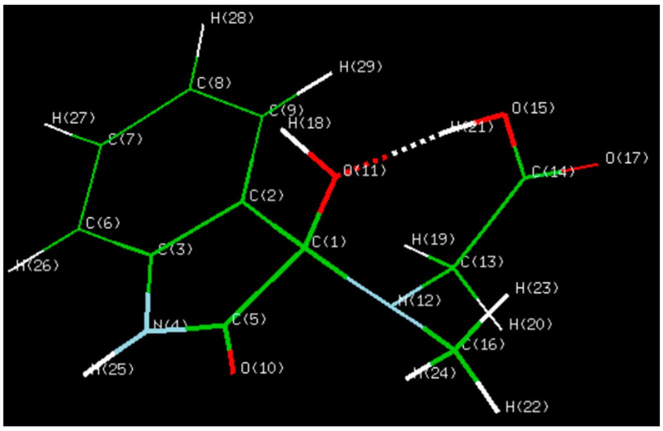

		x	y	z	q
1	C	-0.038877	0.780542	0.314772	0.195650
2	C	0.957957	-0.351431	0.194309	-0.029116
3	C	2.171358	0.168803	-0.263931	0.099078
4	N	2.069750	1.560458	-0.399287	-0.409613
5	C	0.873292	2.026328	0.056264	0.278602
6	C	3.278832	-0.635681	-0.477059	-0.102059
7	C	3.145645	-1.997706	-0.192136	-0.090036
8	C	1.953921	-2.525139	0.302740	-0.109197
9	C	0.845655	-1.696198	0.505127	-0.081619
10	O	0.569463	3.193623	0.215667	-0.371839
11	O	-0.660782	0.867268	1.594156	-0.398576
12	N	-1.056608	0.771717	-0.706179	-0.361690
13	C	-1.641900	-0.487180	-1.123025	-0.046423
14	C	-2.642120	-1.176428	-0.192371	0.256616
15	O	-2.594150	-0.930431	1.113056	-0.400592
16	C	-2.066458	1.828031	-0.588397	-0.134711
17	O	-3.443465	-1.975723	-0.630650	-0.402953
18	H	-0.008987	0.713754	2.296826	0.380330
19	H	-0.863997	-1.223981	-1.342090	0.125191
20	H	-2.183081	-0.317073	-2.057987	0.128542
21	H	-1.899601	-0.273020	1.367676	0.330976
22	H	-2.540124	1.963358	-1.564298	0.106290
23	H	-2.848337	1.582923	0.143803	0.092714
24	H	-1.598906	2.766134	-0.294384	0.102364
25	H	2.846276	2.173314	-0.619159	0.370065
26	H	4.214270	-0.219914	-0.835718	0.123232
27	H	3.996761	-2.653134	-0.348268	0.116649
28	H	1.885166	-3.582420	0.534616	0.112968
29	H	-0.083788	-2.096702	0.901638	0.119179

TS23

log file: geo8_ts5.log
E(RM06-2X) = -836.497696197 A.U.
Zero-point correction= 0.310959
Thermal correction to Energy= 0.331562
Thermal correction to Enthalpy= 0.332506
Thermal correction to Gibbs Free Energy= 0.262067
Sum of electronic and zero-point Energies= -991.203362
Sum of electronic and thermal Energies= -991.182759
Sum of electronic and thermal Enthalpies= -991.181815
Sum of electronic and thermal Free Energies= -991.252255

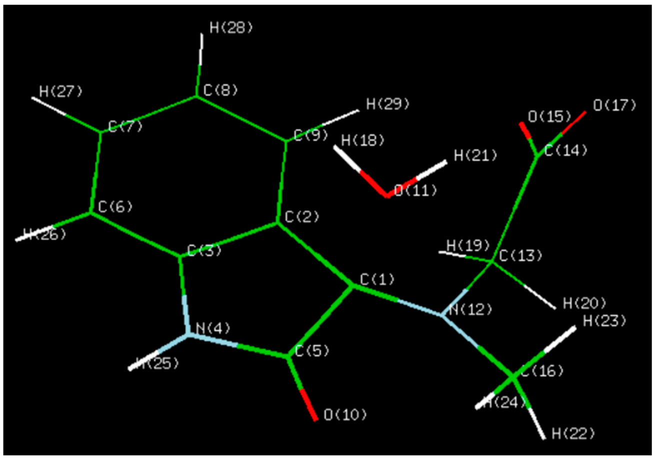

		x	y	z	q
1	C	-0.088531	0.759793	-0.105566	0.229293
2	C	0.898515	-0.351260	-0.142074	-0.024178
3	C	2.171376	0.239581	-0.111697	0.109779
4	N	2.058057	1.632832	-0.073285	-0.401351
5	C	0.767738	2.053760	-0.044771	0.293737
6	C	3.335479	-0.508507	-0.104408	-0.094239
7	C	3.202841	-1.898016	-0.103710	-0.075909
8	C	1.948528	-2.506391	-0.095485	-0.102125
9	C	0.783218	-1.738389	-0.103877	-0.071404
10	O	0.386797	3.204159	0.009422	-0.346891
11	O	-0.550848	0.818271	1.718656	-0.518549
12	N	-1.252384	0.750812	-0.742288	-0.288785
13	C	-1.903320	-0.505067	-1.097840	-0.043777
14	C	-2.500791	-1.282643	0.092111	0.212111
15	O	-2.299674	-0.847260	1.273997	-0.435944
16	C	-2.181634	1.883890	-0.658705	-0.111415
17	O	-3.135605	-2.301487	-0.201420	-0.479227
18	H	0.140717	0.403754	2.260093	0.365985
19	H	-1.207682	-1.151625	-1.636567	0.126017
20	H	-2.717836	-0.268401	-1.785734	0.135115
21	H	-1.331612	0.097454	1.592232	0.304926
22	H	-2.575996	2.092116	-1.655399	0.118757
23	H	-3.006738	1.599720	0.002645	0.116154
24	H	-1.685420	2.763882	-0.264231	0.114324
25	H	2.840314	2.275134	-0.018611	0.377769
26	H	4.307601	-0.028297	-0.090480	0.128629
27	H	4.096843	-2.513394	-0.097833	0.122453
28	H	1.872095	-3.587681	-0.074351	0.117913
29	H	-0.177869	-2.238139	-0.071867	0.120828

48

log file: geo8_ts5_m2.log
E(RM06-2X) = -836.511805988 A.U.
Zero-point correction= 0.310959
Thermal correction to Energy= 0.331562
Thermal correction to Enthalpy= 0.332506
Thermal correction to Gibbs Free Energy= 0.262067
Sum of electronic and zero-point Energies= -991.203362
Sum of electronic and thermal Energies= -991.182759
Sum of electronic and thermal Enthalpies= -991.181815
Sum of electronic and thermal Free Energies= -991.252255

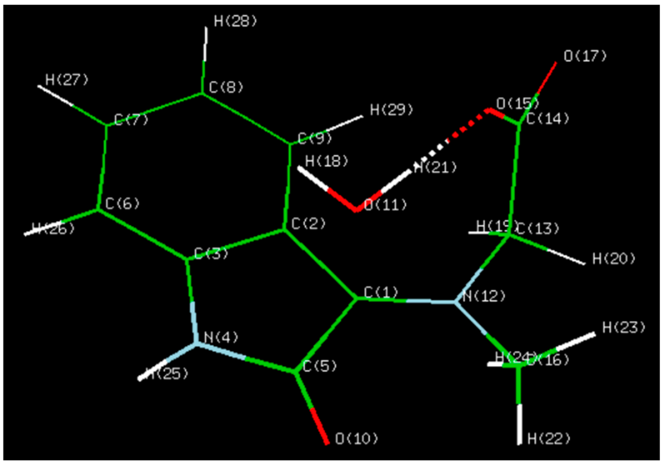

		x	y	z	q
1	C	-0.152024	0.715755	-0.494418	0.217916
2	C	0.860465	-0.318221	-0.398533	-0.024986
3	C	2.067502	0.354938	-0.103076	0.124195
4	N	1.861913	1.731467	-0.042891	-0.402312
5	C	0.561065	2.059612	-0.261691	0.297620
6	C	3.257197	-0.319868	0.103445	-0.089999
7	C	3.225547	-1.711584	0.021262	-0.059454
8	C	2.040992	-2.403236	-0.245402	-0.098313
9	C	0.848319	-1.718978	-0.450575	-0.061673
10	O	0.080123	3.168459	-0.330942	-0.330985
11	O	-0.435374	1.174312	2.127976	-0.669426
12	N	-1.414056	0.634457	-0.746316	-0.208520
13	C	-2.028061	-0.657331	-1.036589	-0.037787
14	C	-2.239826	-1.512200	0.248146	0.199666
15	O	-1.980935	-0.979388	1.352931	-0.449083
16	C	-2.329759	1.775640	-0.576265	-0.092730
17	O	-2.652862	-2.661874	0.019459	-0.495966
18	H	0.315524	0.761624	2.573558	0.341682
19	H	-1.406794	-1.197537	-1.752484	0.133163
20	H	-2.996758	-0.472145	-1.501565	0.142363
21	H	-1.027044	0.424144	1.908264	0.291391
22	H	-2.299825	2.409861	-1.464237	0.132030
23	H	-3.329102	1.365693	-0.444919	0.134877
24	H	-2.038962	2.350751	0.300179	0.124800
25	H	2.583922	2.420453	0.132577	0.380605
26	H	4.171723	0.217769	0.327219	0.131700
27	H	4.143885	-2.268507	0.178392	0.127330
28	H	2.046901	-3.486294	-0.287817	0.120952
29	H	-0.054588	-2.286524	-0.637925	0.120969

TS24

log file: geo8_ts6.log

E(RM06-2X) = -836.478960890 A.U.

Zero-point correction=	0.310959
Thermal correction to Energy=	0.331562
Thermal correction to Enthalpy=	0.332506
Thermal correction to Gibbs Free Energy=	0.262067
Sum of electronic and zero-point Energies=	-991.203362
Sum of electronic and thermal Energies=	-991.182759
Sum of electronic and thermal Enthalpies=	-991.181815
Sum of electronic and thermal Free Energies=	-991.252255

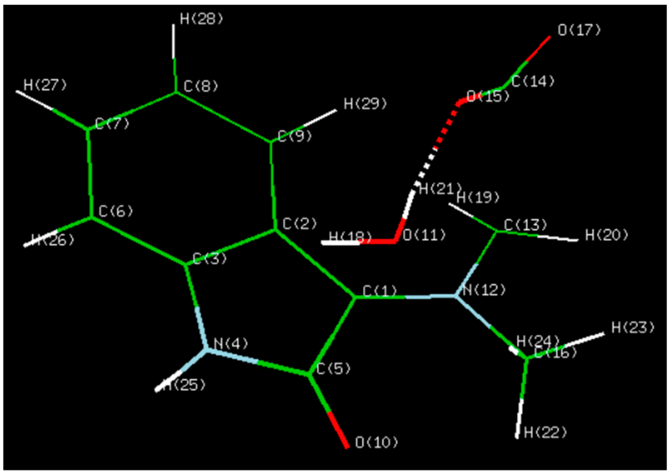

		x	y	z	q
1	C	-0.157782	0.649067	-0.624903	0.096759
2	C	0.928177	-0.296666	-0.459650	-0.034852
3	C	2.051794	0.476857	-0.072201	0.097386
4	N	1.694336	1.819757	-0.031553	-0.414983
5	C	0.373684	2.010578	-0.353407	0.257149
6	C	3.278692	-0.084459	0.229187	-0.106195
7	C	3.392010	-1.475306	0.154390	-0.097329
8	C	2.299870	-2.263361	-0.201878	-0.115694
9	C	1.064349	-1.689207	-0.510639	-0.097144
10	O	-0.182354	3.101287	-0.418995	-0.395173
11	O	-0.973064	1.630057	2.329240	-0.682441
12	N	-1.423626	0.415912	-0.970694	-0.210920
13	C	-1.890511	-0.800807	-1.306061	-0.067048
14	C	-2.092450	-1.827798	0.582683	0.344152
15	O	-1.607708	-1.101289	1.389280	-0.273841
16	C	-2.421483	1.495315	-0.800824	-0.097992
17	O	-2.622700	-2.835277	0.248116	-0.304288
18	H	-0.012241	1.700082	2.394201	0.336758
19	H	-1.185540	-1.498383	-1.736875	0.126987
20	H	-2.918212	-0.836010	-1.641092	0.132115
21	H	-1.119768	0.724697	2.020685	0.304589
22	H	-2.260980	2.252183	-1.567442	0.127357
23	H	-3.408779	1.052034	-0.903623	0.131064
24	H	-2.293296	1.938512	0.185710	0.116396
25	H	2.318455	2.585525	0.188310	0.368721
26	H	4.117571	0.539202	0.519853	0.121493
27	H	4.344010	-1.944010	0.382292	0.114278
28	H	2.405373	-3.342110	-0.246284	0.110722
29	H	0.246939	-2.344898	-0.781319	0.111971

25

log file: geo8_ts6_m2.log
E(RM06-2X) = -836.499686769 A.U.
Zero-point correction= 0.310959
Thermal correction to Energy= 0.331562
Thermal correction to Enthalpy= 0.332506
Thermal correction to Gibbs Free Energy= 0.262067
Sum of electronic and zero-point Energies= -991.203362
Sum of electronic and thermal Energies= -991.182759
Sum of electronic and thermal Enthalpies= -991.181815
Sum of electronic and thermal Free Energies= -991.252255

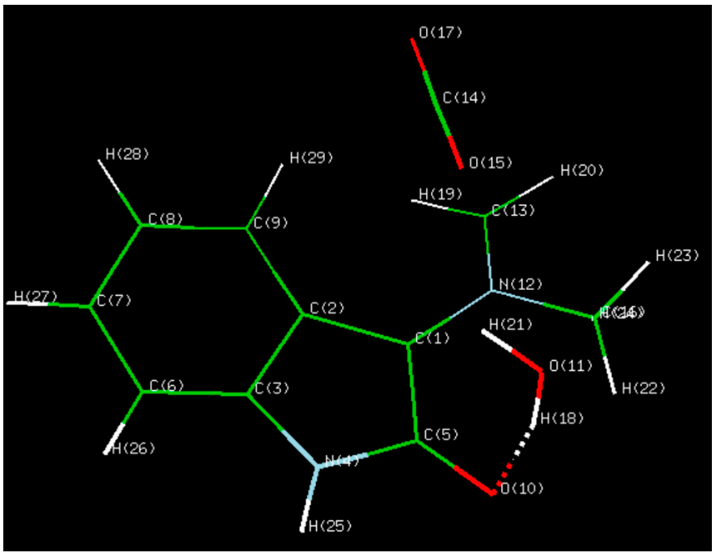

		x	y	z	q
1	C	-0.306846	0.404528	-0.771423	0.012930
2	C	1.056434	0.029250	-0.484158	-0.043373
3	C	1.678264	1.206629	0.015535	0.079747
4	N	0.742444	2.223819	0.018681	-0.426464
5	C	-0.491918	1.785084	-0.425819	0.215862
6	C	2.998906	1.238683	0.435064	-0.117174
7	C	3.733168	0.053264	0.376854	-0.123793
8	C	3.141319	-1.124178	-0.083041	-0.128004
9	C	1.814211	-1.150474	-0.515868	-0.121670
10	O	-1.500595	2.529016	-0.462976	-0.442167
11	O	-2.555418	1.512700	1.976412	-0.700814
12	N	-1.314535	-0.409835	-1.266893	-0.207715
13	C	-1.110193	-1.554264	-1.842437	0.024689
14	C	-0.965761	-2.328775	1.183102	0.439091
15	O	-1.386382	-1.339625	1.627294	-0.203256
16	C	-2.713591	0.040582	-1.091970	-0.101446
17	O	-0.554227	-3.324769	0.745926	-0.220258
18	H	-2.302970	2.029269	1.190159	0.294686
19	H	-0.102874	-1.871500	-2.067907	0.144132
20	H	-1.963628	-2.152726	-2.131080	0.152727
21	H	-1.770296	0.978830	2.153514	0.320966
22	H	-2.875822	0.913666	-1.721342	0.124608
23	H	-3.370983	-0.774516	-1.386034	0.130196
24	H	-2.864218	0.313518	-0.048447	0.111650
25	H	0.896775	3.168642	0.344158	0.359950
26	H	3.437165	2.160068	0.805909	0.114293
27	H	4.770019	0.049534	0.697322	0.104914
28	H	3.721668	-2.041092	-0.111846	0.103477
29	H	1.398820	-2.092110	-0.855204	0.102215

26

log file: geo2.log

E(RM06-2X) = -836.519413970 A.U.

Zero-point correction= 0.310959

Thermal correction to Energy= 0.331562

Thermal correction to Enthalpy= 0.332506

Thermal correction to Gibbs Free Energy= 0.262067

Sum of electronic and zero-point Energies= -991.203362

Sum of electronic and thermal Energies= -991.182759

Sum of electronic and thermal Enthalpies= -991.181815

Sum of electronic and thermal Free Energies= -991.252255

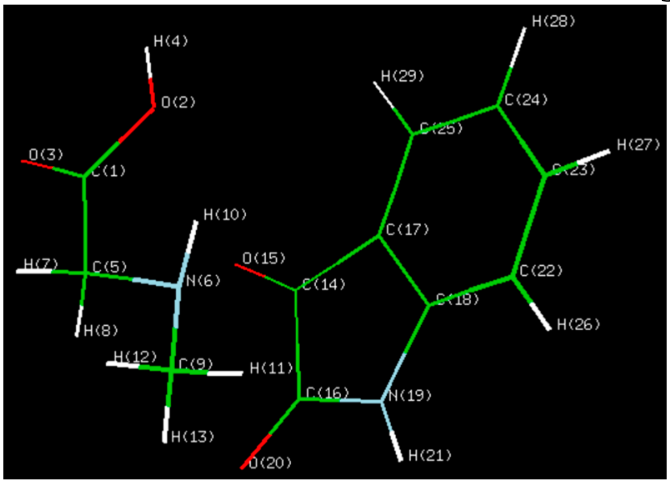

		x	y	z	q
1	C	2.591452	-1.161709	0.235927	0.269066
2	O	1.602892	-1.950521	0.665552	-0.364701
3	O	3.524679	-1.566467	-0.421952	-0.392497
4	H	1.764148	-2.853277	0.339480	0.392170
5	C	2.466103	0.265113	0.713675	-0.045601
6	N	1.140279	0.602353	1.193267	-0.493134
7	H	3.252243	0.399484	1.476451	0.119819
8	H	2.726469	0.912809	-0.131921	0.118729
9	C	1.110885	1.902882	1.850322	-0.130551
10	H	0.816793	-0.115591	1.836921	0.309564
11	H	0.110063	2.082343	2.250350	0.102977
12	H	1.837349	1.984762	2.672382	0.091133
13	H	1.337461	2.685167	1.118048	0.094665
14	C	0.003478	0.096505	-1.187050	0.189237
15	O	0.905007	-0.435312	-1.798600	-0.314069
16	C	-0.099701	1.628370	-0.978700	0.273183
17	C	-1.209180	-0.458002	-0.570317	-0.043658
18	C	-1.954036	0.629493	-0.086871	0.109427
19	N	-1.294036	1.838665	-0.354241	-0.410081
20	O	0.720435	2.452678	-1.333862	-0.367437
21	H	-1.620711	2.748653	-0.052562	0.370256
22	C	-3.167095	0.438927	0.553833	-0.100164
23	C	-3.615453	-0.878201	0.694918	-0.078149
24	C	-2.883442	-1.967553	0.214735	-0.108186
25	C	-1.663197	-1.761246	-0.428453	-0.073028
26	H	-3.744843	1.276592	0.929393	0.124347
27	H	-4.563800	-1.055314	1.193091	0.119368
28	H	-3.267800	-2.973344	0.344085	0.113885
29	H	-1.074057	-2.589904	-0.810112	0.123423

TS8

log file: geo2_ts1.log

E(RM06-2X) = -836.473521938 A.U.

Zero-point correction= 0.310959

Thermal correction to Energy= 0.331562

Thermal correction to Enthalpy= 0.332506

Thermal correction to Gibbs Free Energy= 0.262067

Sum of electronic and zero-point Energies= -991.203362

Sum of electronic and thermal Energies= -991.182759

Sum of electronic and thermal Enthalpies= -991.181815

Sum of electronic and thermal Free Energies= -991.252255

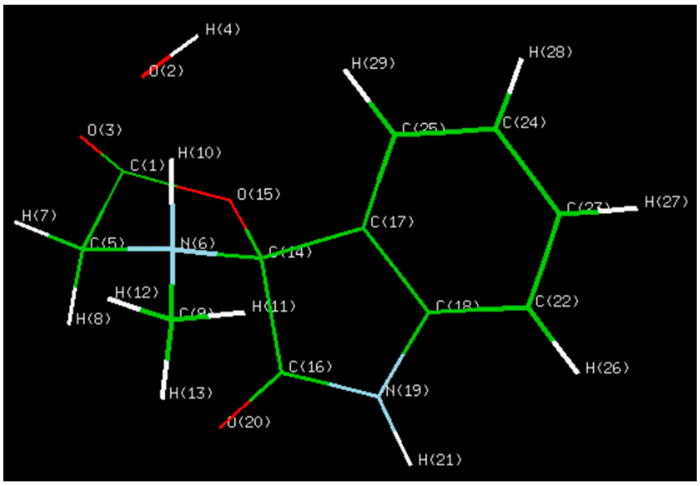

		x	y	z	q
1	C	2.447170	-0.590664	-0.591666	0.242917
2	O	1.938100	-2.053394	0.304332	-0.563769
3	O	3.359215	-0.809037	-1.374518	-0.481734
4	H	1.403857	-2.561952	-0.322574	0.327191
5	C	2.490864	0.241544	0.689723	-0.045928
6	N	1.087505	0.008281	1.106086	-0.341768
7	H	3.172296	-0.170935	1.432412	0.142212
8	H	2.688921	1.294682	0.484876	0.128164
9	C	0.605056	0.680002	2.318596	-0.101044
10	H	1.213848	-1.173003	1.037331	0.313895
11	H	-0.444773	0.424609	2.473119	0.121525
12	H	1.194561	0.322537	3.162792	0.131834
13	H	0.722658	1.762385	2.220476	0.119295
14	C	0.310413	0.242290	-0.154030	0.194560
15	O	1.154191	-0.250996	-1.159965	-0.259616
16	C	0.035320	1.763876	-0.373359	0.278770
17	C	-1.075737	-0.341664	-0.116285	-0.032892
18	C	-1.992838	0.701994	-0.270390	0.101213
19	N	-1.311181	1.924384	-0.405718	-0.401343
20	O	0.890044	2.620514	-0.493272	-0.362350
21	H	-1.758854	2.820000	-0.563605	0.374906
22	C	-3.359180	0.480095	-0.275957	-0.098408
23	C	-3.791411	-0.840120	-0.118708	-0.085536
24	C	-2.888295	-1.890490	0.039872	-0.105976
25	C	-1.511760	-1.645544	0.043030	-0.079237
26	H	-4.061737	1.297388	-0.397251	0.125405
27	H	-4.856913	-1.047366	-0.119679	0.118440
28	H	-3.255039	-2.903943	0.160809	0.114331
29	H	-0.803099	-2.457648	0.171150	0.124939

27

log file: geo2_ts1_m2.log
E(RM06-2X) = -836.526234081 A.U.
Zero-point correction= 0.310959
Thermal correction to Energy= 0.331562
Thermal correction to Enthalpy= 0.332506
Thermal correction to Gibbs Free Energy= 0.262067
Sum of electronic and zero-point Energies= -991.203362
Sum of electronic and thermal Energies= -991.182759
Sum of electronic and thermal Enthalpies= -991.181815
Sum of electronic and thermal Free Energies= -991.252255

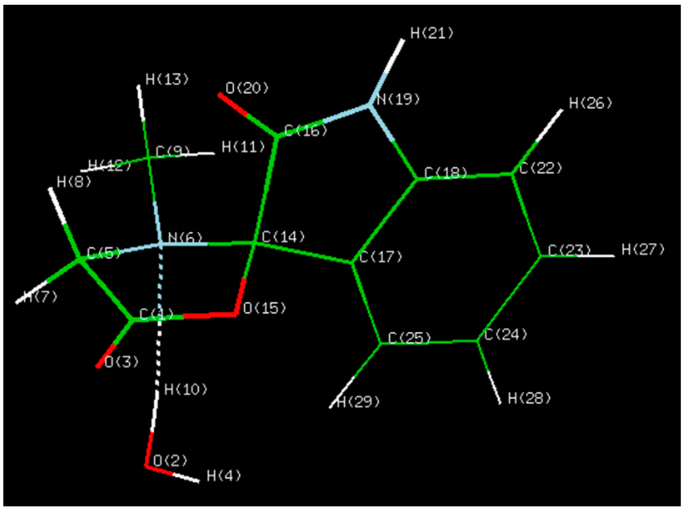

		x	y	z	q
1	C	-2.443087	0.151798	-0.764727	0.278089
2	O	-1.695841	2.871035	0.267180	-0.691312
3	O	-3.340349	0.331264	-1.539957	-0.352527
4	H	-1.350369	2.770780	-0.629264	0.332236
5	C	-2.488746	-0.097416	0.723525	-0.039860
6	N	-1.095339	0.127080	1.102709	-0.343017
7	H	-3.162705	0.613349	1.206806	0.136402
8	H	-2.840345	-1.122125	0.906925	0.126797
9	C	-0.681090	-0.503293	2.350840	-0.118760
10	H	-1.301026	2.126617	0.754947	0.295554
11	H	0.377870	-0.296840	2.526053	0.109769
12	H	-1.257910	-0.069051	3.169523	0.117133
13	H	-0.844638	-1.590594	2.343053	0.097978
14	C	-0.318343	-0.251529	-0.071288	0.197514
15	O	-1.141403	0.153750	-1.164673	-0.193819
16	C	-0.092270	-1.790949	-0.209581	0.273868
17	C	1.079921	0.290210	-0.123953	-0.032018
18	C	1.964160	-0.788756	-0.219611	0.100815
19	N	1.247080	-1.999776	-0.251032	-0.401978
20	O	-0.985948	-2.616071	-0.261489	-0.369256
21	H	1.668479	-2.916929	-0.344794	0.373783
22	C	3.335543	-0.604924	-0.262819	-0.099526
23	C	3.804706	0.711314	-0.208218	-0.086278
24	C	2.933493	1.795285	-0.106536	-0.106921
25	C	1.551350	1.589095	-0.057368	-0.080429
26	H	4.014946	-1.447269	-0.335083	0.124728
27	H	4.875140	0.888420	-0.242416	0.117882
28	H	3.329991	2.803802	-0.062028	0.113688
29	H	0.862881	2.423865	0.034467	0.119469

TS9

log file: geo2_ts5.log
E(RM06-2X) = -836.522751974 A.U.
Zero-point correction= 0.310959
Thermal correction to Energy= 0.331562
Thermal correction to Enthalpy= 0.332506
Thermal correction to Gibbs Free Energy= 0.262067
Sum of electronic and zero-point Energies= -991.203362
Sum of electronic and thermal Energies= -991.182759
Sum of electronic and thermal Enthalpies= -991.181815
Sum of electronic and thermal Free Energies= -991.252255

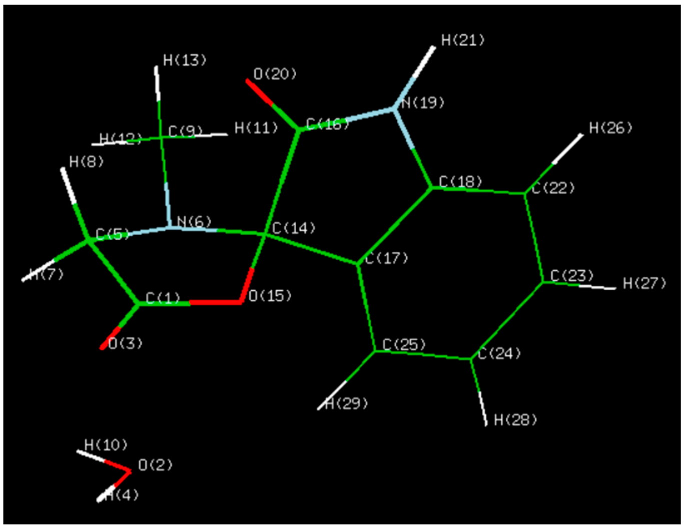

		x	y	z	q
1	C	-2.406929	0.114047	-0.684666	0.276212
2	O	-2.087660	2.773886	0.093083	-0.653658
3	O	-3.321272	0.339577	-1.431299	-0.363406
4	H	-2.869776	2.884536	-0.462742	0.346760
5	C	-2.431043	-0.269401	0.772896	-0.041890
6	N	-1.040533	-0.087110	1.160087	-0.362601
7	H	-3.105921	0.388191	1.325777	0.132925
8	H	-2.787939	-1.307208	0.858568	0.121217
9	C	-0.608478	-0.864495	2.312251	-0.123556
10	H	-2.439831	2.755320	0.991638	0.348788
11	H	0.453120	-0.678557	2.498701	0.106773
12	H	-1.170670	-0.536605	3.189281	0.112434
13	H	-0.767471	-1.945627	2.180301	0.092285
14	C	-0.265848	-0.299863	-0.052524	0.195659
15	O	-1.118351	0.146264	-1.104019	-0.197368
16	C	0.067881	-1.798553	-0.338448	0.271415
17	C	1.089804	0.342112	-0.070325	-0.033687
18	C	2.049227	-0.653910	-0.270408	0.098808
19	N	1.419575	-1.906506	-0.407807	-0.403971
20	O	-0.764161	-2.679804	-0.458210	-0.375480
21	H	1.903953	-2.778294	-0.588063	0.371943
22	C	3.402033	-0.361196	-0.310458	-0.101790
23	C	3.768963	0.978848	-0.147031	-0.089087
24	C	2.819224	1.980836	0.051622	-0.108863
25	C	1.457770	1.663765	0.097005	-0.084075
26	H	4.143646	-1.137835	-0.463015	0.123283
27	H	4.822155	1.240506	-0.176129	0.116463
28	H	3.139480	3.010012	0.173405	0.112379
29	H	0.701895	2.428343	0.250406	0.112038

28

log file: geo2_ts4_m1.log
E(RM06-2X) = -836.523277210 A.U.
Zero-point correction= 0.310959
Thermal correction to Energy= 0.331562
Thermal correction to Enthalpy= 0.332506
Thermal correction to Gibbs Free Energy= 0.262067
Sum of electronic and zero-point Energies= -991.203362
Sum of electronic and thermal Energies= -991.182759
Sum of electronic and thermal Enthalpies= -991.181815
Sum of electronic and thermal Free Energies= -991.252255

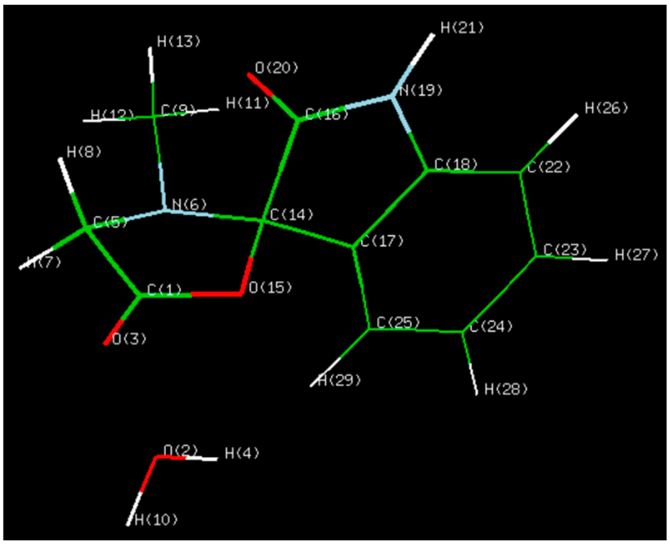

		x	y	z	q
1	C	-2.446067	0.222408	-0.614238	0.276114
2	O	-1.719309	2.841474	0.081746	-0.654541
3	O	-3.359181	0.501189	-1.341892	-0.362382
4	H	-1.276762	2.650014	-0.755406	0.334667
5	C	-2.461992	-0.215083	0.827919	-0.042696
6	N	-1.059101	-0.109729	1.199718	-0.363800
7	H	-3.096973	0.452100	1.416054	0.130460
8	H	-2.865601	-1.237716	0.881164	0.122135
9	C	-0.642409	-0.961182	2.303808	-0.123267
10	H	-2.210192	3.654882	-0.093024	0.353975
11	H	0.428592	-0.827609	2.481148	0.106926
12	H	-1.177669	-0.655982	3.205482	0.112840
13	H	-0.847399	-2.026950	2.120224	0.092239
14	C	-0.308692	-0.286639	-0.031863	0.196545
15	O	-1.157444	0.248538	-1.045861	-0.190933
16	C	-0.039912	-1.778848	-0.405432	0.271411
17	C	1.070900	0.302473	-0.037247	-0.033277
18	C	1.985553	-0.716543	-0.316355	0.098968
19	N	1.304594	-1.934044	-0.511407	-0.403740
20	O	-0.909199	-2.618702	-0.554149	-0.374917
21	H	1.751765	-2.812536	-0.746964	0.372138
22	C	3.347929	-0.475100	-0.367709	-0.101429
23	C	3.771972	0.836608	-0.129889	-0.088755
24	C	2.867744	1.860278	0.152662	-0.108805
25	C	1.495497	1.595032	0.207971	-0.083825
26	H	4.054321	-1.269573	-0.582641	0.123502
27	H	4.834138	1.058149	-0.165392	0.116629
28	H	3.231987	2.865820	0.333849	0.112336
29	H	0.773265	2.375393	0.430822	0.111471

TS10

log file: geo2_ts4.log
E(RM06-2X) = -836.513415753 A.U.
Zero-point correction= 0.310959
Thermal correction to Energy= 0.331562
Thermal correction to Enthalpy= 0.332506
Thermal correction to Gibbs Free Energy= 0.262067
Sum of electronic and zero-point Energies= -991.203362
Sum of electronic and thermal Energies= -991.182759
Sum of electronic and thermal Enthalpies= -991.181815

Sum of electronic and thermal Free Energies=   -991.252255

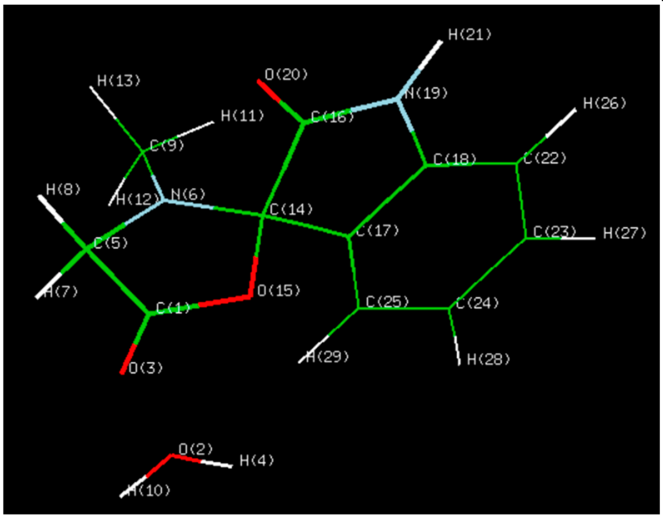

		x	y	z	q
1	C	-2.441948	0.073850	-0.654200	0.279390
2	O	-1.686533	2.745743	-0.308878	-0.648012
3	O	-3.337250	0.287311	-1.428359	-0.367527
4	H	-1.272679	2.528009	-1.154460	0.341661
5	C	-2.495625	-0.068954	0.846360	-0.034227
6	N	-1.110473	-0.210311	1.181759	-0.362812
7	H	-2.944187	0.837258	1.272543	0.125681
8	H	-3.129573	-0.929563	1.107281	0.119116
9	C	-0.603973	-0.024769	2.515887	-0.113410
10	H	-2.595228	2.970744	-0.548162	0.350712
11	H	0.479189	-0.164680	2.516662	0.110195
12	H	-0.820716	0.985117	2.890833	0.096822
13	H	-1.036602	-0.752128	3.213458	0.098566
14	C	-0.312818	-0.436781	0.024108	0.206915
15	O	-1.165798	-0.088357	-1.076060	-0.193716
16	C	0.062424	-1.940615	-0.201258	0.268043
17	C	1.029515	0.240636	-0.045286	-0.032872
18	C	2.005989	-0.733703	-0.270730	0.098958
19	N	1.411142	-2.006609	-0.353324	-0.406761
20	O	-0.740270	-2.854476	-0.223248	-0.386829
21	H	1.919746	-2.869813	-0.504956	0.370126
22	C	3.347976	-0.406685	-0.373127	-0.102532
23	C	3.688754	0.943171	-0.239334	-0.089114
24	C	2.723865	1.922685	-0.005895	-0.109760
25	C	1.373665	1.572618	0.098932	-0.082657
26	H	4.101676	-1.167212	-0.546647	0.122844
27	H	4.733072	1.229945	-0.315401	0.116377
28	H	3.023330	2.959982	0.098969	0.111992
29	H	0.609152	2.320294	0.290459	0.112771

29

log file: geo2_ts2_m1.log
E(RM06-2X) = -836.520893924   A.U.
Zero-point correction=   0.310959
Thermal correction to Energy=   0.331562
Thermal correction to Enthalpy=   0.332506
Thermal correction to Gibbs Free Energy=   0.262067
Sum of electronic and zero-point Energies=   -991.203362
Sum of electronic and thermal Energies=   -991.182759
Sum of electronic and thermal Enthalpies=   -991.181815

Sum of electronic and thermal Free Energies= -991.252255

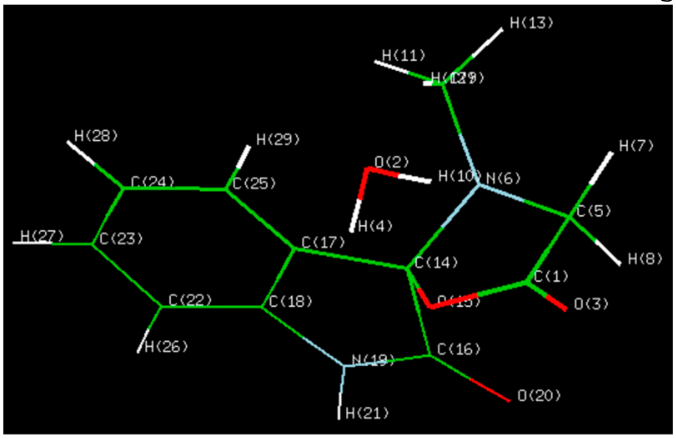

		x	y	z	q
1	C	-2.449681	-0.073181	-0.475559	0.277040
2	O	-1.618520	2.958833	-0.783358	-0.673984
3	O	-3.395788	0.207875	-1.163319	-0.354899
4	H	-1.181932	2.242231	-1.263374	0.320051
5	C	-2.402115	-0.599961	0.945206	-0.043422
6	N	-1.013941	-0.372350	1.366722	-0.364365
7	H	-3.114766	-0.070694	1.578094	0.141684
8	H	-2.657375	-1.662834	0.927209	0.129250
9	C	-0.892571	0.919613	2.059729	-0.130662
10	H	-2.558135	2.791075	-0.933667	0.339589
11	H	0.138692	1.073844	2.378122	0.103396
12	H	-1.215947	1.767450	1.438977	0.087399
13	H	-1.519797	0.876169	2.951936	0.111490
14	C	-0.286809	-0.436695	0.106951	0.195670
15	O	-1.176393	0.058160	-0.920392	-0.191883
16	C	0.016310	-1.932071	-0.198507	0.278500
17	C	1.072783	0.195509	0.034721	-0.028166
18	C	2.014645	-0.825545	-0.130977	0.101545
19	N	1.367121	-2.069556	-0.214576	-0.403807
20	O	-0.822761	-2.795872	-0.369676	-0.368929
21	H	1.832829	-2.949589	-0.403339	0.373218
22	C	3.370284	-0.561046	-0.230159	-0.100254
23	C	3.765332	0.778613	-0.180282	-0.086633
24	C	2.835336	1.808943	-0.048407	-0.107489
25	C	1.471156	1.521991	0.058558	-0.078072
26	H	4.091195	-1.361618	-0.355428	0.124557
27	H	4.821219	1.018200	-0.258278	0.117837
28	H	3.170790	2.840094	-0.030336	0.113738
29	H	0.740638	2.320016	0.151382	0.117591

TS11

log file: geo2_ts2.log
E(RM06-2X) = -836.499055180 A.U.
Zero-point correction= 0.310959
Thermal correction to Energy= 0.331562
Thermal correction to Enthalpy= 0.332506
Thermal correction to Gibbs Free Energy= 0.262067
Sum of electronic and zero-point Energies= -991.203362
Sum of electronic and thermal Energies= -991.182759
Sum of electronic and thermal Enthalpies= -991.181815
Sum of electronic and thermal Free Energies= -991.252255

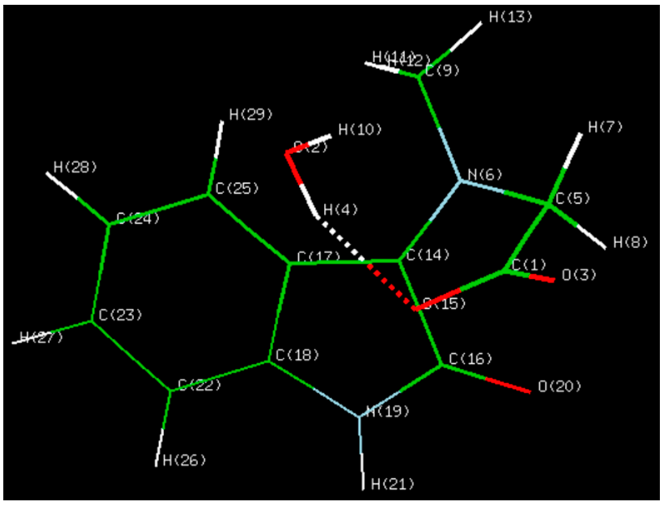

		x	y	z	q
1	C	-2.427188	-0.008485	-0.732237	0.230432
2	O	-0.682874	2.807393	-1.173154	-0.682912
3	O	-3.458834	0.095578	-1.391215	-0.446140
4	H	-0.690192	1.847065	-1.316952	0.299373
5	C	-2.522000	-0.159401	0.809659	-0.037144
6	N	-1.190753	0.148567	1.317139	-0.285892
7	H	-3.247464	0.537426	1.228083	0.138512
8	H	-2.826755	-1.182238	1.032870	0.129594
9	C	-1.027658	1.471797	1.924069	-0.104141
10	H	-1.579178	3.075380	-1.413855	0.343659
11	H	-0.069058	1.518048	2.440355	0.120811
12	H	-1.091008	2.254214	1.160261	0.106807
13	H	-1.824420	1.601367	2.656157	0.125361
14	C	-0.246565	-0.360265	0.536034	0.219565
15	O	-1.222771	-0.022901	-1.183221	-0.327221
16	C	-0.242437	-1.864885	0.187101	0.289168
17	C	1.129444	0.095140	0.343211	-0.027852
18	C	1.851471	-1.010351	-0.145192	0.116348
19	N	1.028226	-2.136514	-0.212876	-0.402452
20	O	-1.165067	-2.647998	0.274791	-0.348248
21	H	1.322918	-3.053840	-0.526960	0.377660
22	C	3.185578	-0.919061	-0.500412	-0.093751
23	C	3.795930	0.329583	-0.371596	-0.068007
24	C	3.091371	1.446918	0.080863	-0.102180
25	C	1.747838	1.342870	0.434251	-0.064402
26	H	3.727368	-1.782877	-0.869106	0.129170
27	H	4.842499	0.432492	-0.640810	0.124081
28	H	3.590613	2.406539	0.152478	0.117984
29	H	1.204040	2.222326	0.755530	0.121801

30

```
log file: geo2_ts2_m2.log
E(RM06-2X) =  -836.508406854     A.U.
Zero-point correction=                          0.310959
Thermal correction to Energy=                   0.331562
Thermal correction to Enthalpy=                 0.332506
Thermal correction to Gibbs Free Energy=        0.262067
Sum of electronic and zero-point Energies=          -991.203362
Sum of electronic and thermal Energies=             -991.182759
Sum of electronic and thermal Enthalpies=           -991.181815
Sum of electronic and thermal Free Energies=        -991.252255
```

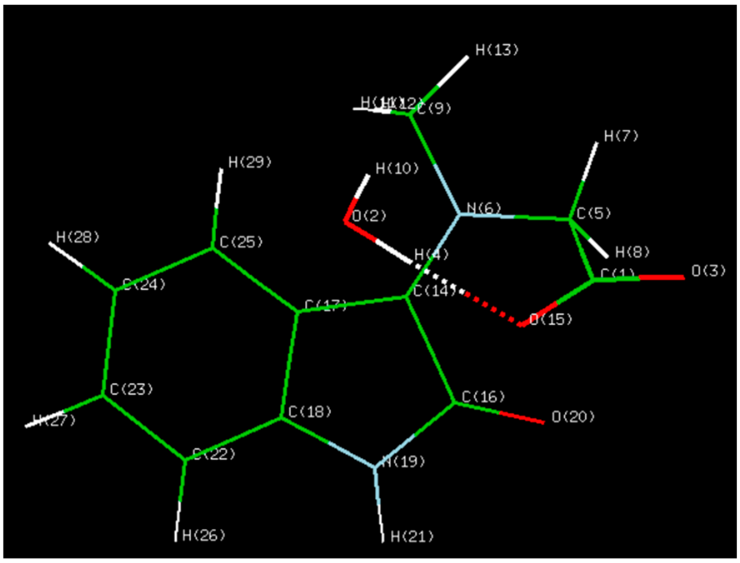

		x	y	z	q
1	C	-2.877385	-0.071674	-0.517914	0.203899
2	O	-0.308647	2.086475	-1.725685	-0.690983
3	O	-4.094013	-0.289305	-0.627751	-0.493202
4	H	-0.892341	1.305654	-1.636001	0.287525
5	C	-2.438064	0.231036	0.951737	-0.041672
6	N	-1.036322	0.636741	1.044369	-0.205997
7	H	-3.046534	1.039168	1.351773	0.136757
8	H	-2.607020	-0.674203	1.534876	0.132952
9	C	-0.753617	2.023052	1.436692	-0.090520
10	H	-0.902853	2.783386	-2.030513	0.340147
11	H	0.112554	2.037965	2.098918	0.132245
12	H	-0.564521	2.605024	0.530386	0.122936
13	H	-1.619874	2.411375	1.967053	0.136531
14	C	-0.093320	-0.134635	0.611680	0.214246
15	O	-1.993797	-0.086447	-1.409736	-0.439109
16	C	-0.308153	-1.636408	0.325321	0.299664
17	C	1.308810	0.136465	0.371409	-0.025251
18	C	1.874473	-1.085129	-0.056499	0.123480
19	N	0.911932	-2.093047	-0.062651	-0.399816
20	O	-1.317456	-2.288760	0.476826	-0.322919
21	H	1.083706	-3.061776	-0.306065	0.381096
22	C	3.203751	-1.195020	-0.421328	-0.089960
23	C	3.979345	-0.037021	-0.363293	-0.058517
24	C	3.438739	1.190230	0.030850	-0.099145
25	C	2.100864	1.292194	0.393276	-0.060626
26	H	3.617807	-2.143293	-0.744667	0.131874
27	H	5.027026	-0.092522	-0.641731	0.127371
28	H	4.065724	2.074253	0.048708	0.120310
29	H	1.695701	2.254870	0.674432	0.126704

TS12

log file: geo2_ts3.log
E(RM06-2X) = -836.480375615 A.U.
Zero-point correction= 0.310959
Thermal correction to Energy= 0.331562
Thermal correction to Enthalpy= 0.332506
Thermal correction to Gibbs Free Energy= 0.262067
Sum of electronic and zero-point Energies= -991.203362
Sum of electronic and thermal Energies= -991.182759
Sum of electronic and thermal Enthalpies= -991.181815

Sum of electronic and thermal Free Energies= -991.252255

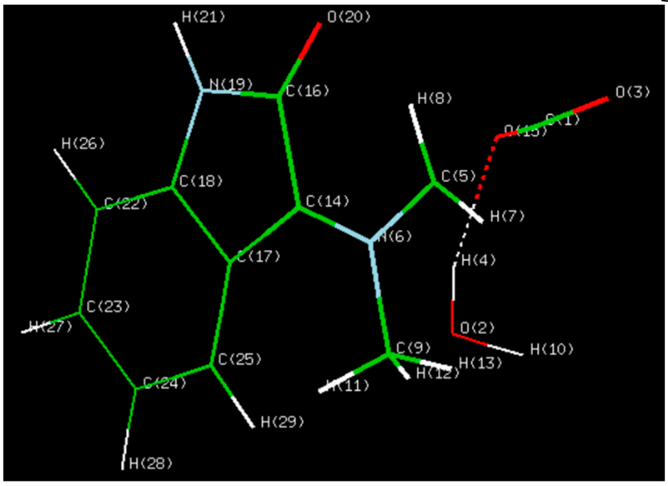

		x	y	z	q
1	C	-3.062031	-0.011083	0.733049	0.340533
2	O	-0.144546	-2.220612	1.781561	-0.676764
3	O	-4.241285	0.014084	0.583910	-0.312590
4	H	-0.628237	-1.389200	1.670761	0.305751
5	C	-2.355728	-0.026906	-1.274810	-0.063777
6	N	-1.084644	-0.459688	-1.159839	-0.211717
7	H	-3.035780	-0.705919	-1.770360	0.130364
8	H	-2.513858	1.040012	-1.335059	0.124678
9	C	-0.826635	-1.882985	-1.444369	-0.093193
10	H	-0.830351	-2.845782	2.049229	0.347114
11	H	-0.066529	-1.949391	-2.224018	0.131892
12	H	-0.486004	-2.360749	-0.523831	0.117177
13	H	-1.749716	-2.340441	-1.790666	0.134011
14	C	-0.080712	0.265382	-0.660174	0.095191
15	O	-2.087580	-0.008311	1.417509	-0.274802
16	C	-0.253454	1.677294	-0.252905	0.250777
17	C	1.302288	-0.105838	-0.402561	-0.032505
18	C	1.910593	1.055052	0.134858	0.094609
19	N	0.973672	2.078242	0.208816	-0.416505
20	O	-1.261730	2.381977	-0.288283	-0.389169
21	H	1.147091	3.006965	0.571264	0.368081
22	C	3.243549	1.098787	0.510571	-0.107878
23	C	4.002316	-0.059185	0.346130	-0.100438
24	C	3.429626	-1.218974	-0.178728	-0.117931
25	C	2.087530	-1.255847	-0.553249	-0.098597
26	H	3.672644	2.007617	0.919426	0.120545
27	H	5.049216	-0.055505	0.632195	0.113019
28	H	4.034331	-2.111977	-0.296646	0.109213
29	H	1.681058	-2.176805	-0.949587	0.112879

31

log file: geo2_ts3_m2.log
E(RM06-2X) = -836.500293335 A.U.
Zero-point correction= 0.310959
Thermal correction to Energy= 0.331562
Thermal correction to Enthalpy= 0.332506
Thermal correction to Gibbs Free Energy= 0.262067
Sum of electronic and zero-point Energies= -991.203362
Sum of electronic and thermal Energies= -991.182759
Sum of electronic and thermal Enthalpies= -991.181815
Sum of electronic and thermal Free Energies= -991.252255

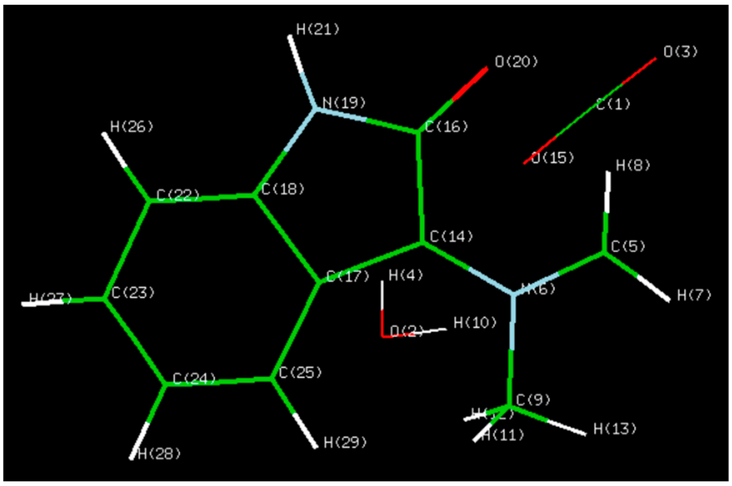

		x	y	z	q
1	C	-3.227098	0.513276	0.923009	0.425322
2	O	0.093782	-1.407931	2.318810	-0.680991
3	O	-4.184513	0.877990	0.373362	-0.234404
4	H	-0.002508	-0.657731	1.715027	0.301435
5	C	-2.122360	-1.091419	-1.455558	0.011861
6	N	-0.906556	-1.215163	-1.012141	-0.209891
7	H	-2.703584	-1.990469	-1.609399	0.147747
8	H	-2.504678	-0.105355	-1.677745	0.132891
9	C	-0.382554	-2.573449	-0.777599	-0.093003
10	H	-0.816841	-1.618835	2.561638	0.337249
11	H	0.411164	-2.767733	-1.499366	0.130731
12	H	-0.000423	-2.619010	0.242496	0.116547
13	H	-1.190066	-3.288735	-0.915930	0.133632
14	C	-0.057019	-0.159203	-0.713020	0.019190
15	O	-2.312332	0.119205	1.526475	-0.214269
16	C	-0.517962	1.200704	-0.676606	0.209191
17	C	1.352012	-0.182870	-0.379621	-0.036733
18	C	1.704335	1.176452	-0.148566	0.078687
19	N	0.580922	1.962502	-0.328616	-0.427267
20	O	-1.658397	1.674890	-0.871749	-0.436620
21	H	0.538997	2.966488	-0.216575	0.359519
22	C	2.986850	1.576563	0.199243	-0.118978
23	C	3.966088	0.594495	0.334001	-0.126032
24	C	3.648948	-0.749575	0.121890	-0.129558
25	C	2.360263	-1.148108	-0.229641	-0.120663
26	H	3.208209	2.626804	0.362932	0.113448
27	H	4.977427	0.877757	0.607509	0.103961
28	H	4.419156	-1.506507	0.233752	0.102344
29	H	2.165252	-2.202472	-0.379417	0.104648

1a

log file: r2.log
E(RM06-2X) = -568.225456526 A.U.
Zero-point correction= 0.310959
Thermal correction to Energy= 0.331562
Thermal correction to Enthalpy= 0.332506
Thermal correction to Gibbs Free Energy= 0.262067
Sum of electronic and zero-point Energies= -991.203362
Sum of electronic and thermal Energies= -991.182759
Sum of electronic and thermal Enthalpies= -991.181815
Sum of electronic and thermal Free Energies= -991.252255

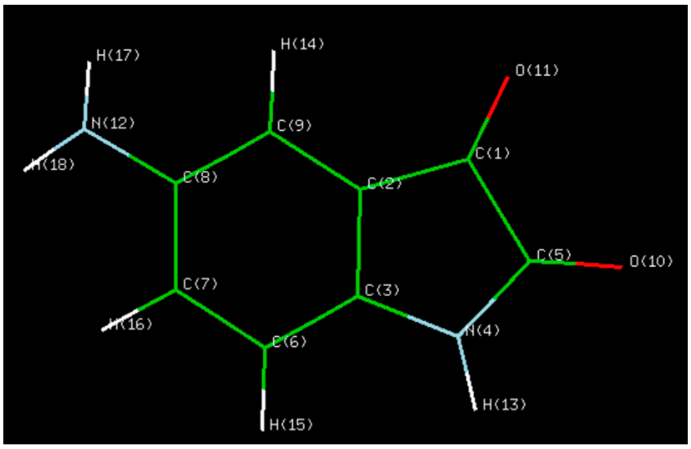

		x	y	z	q
1	C	-1.395208	0.975546	-0.001438	0.196163
2	C	-0.036354	0.424463	-0.006136	-0.044513
3	C	-0.164065	-0.969200	-0.002438	0.090422
4	N	-1.526314	-1.348075	0.002866	-0.409595
5	C	-2.343289	-0.264861	0.003717	0.273013
6	C	0.960817	-1.774268	-0.000164	-0.096679
7	C	2.208075	-1.148025	-0.003029	-0.089544
8	C	2.355492	0.251717	-0.007663	0.087027
9	C	1.202658	1.049520	-0.009158	-0.084006
10	O	-3.557326	-0.222823	0.008088	-0.372063
11	O	-1.796520	2.116031	-0.000644	-0.309389
12	N	3.621601	0.827861	-0.074993	-0.636614
13	H	-1.854189	-2.306404	0.005377	0.371352
14	H	1.274592	2.133712	-0.011405	0.126522
15	H	0.883914	-2.856232	0.006260	0.126110
16	H	3.104955	-1.761708	-0.000989	0.121832
17	H	3.666179	1.768749	0.297446	0.325226
18	H	4.359563	0.248363	0.306498	0.324735

21a

log file: ageo11_ts2_m1.log
E(RM06-2X) = -891.849942175 A.U.
Zero-point correction= 0.310959
Thermal correction to Energy= 0.331562
Thermal correction to Enthalpy= 0.332506
Thermal correction to Gibbs Free Energy= 0.262067
Sum of electronic and zero-point Energies= -991.203362
Sum of electronic and thermal Energies= -991.182759
Sum of electronic and thermal Enthalpies= -991.181815
Sum of electronic and thermal Free Energies= -991.252255

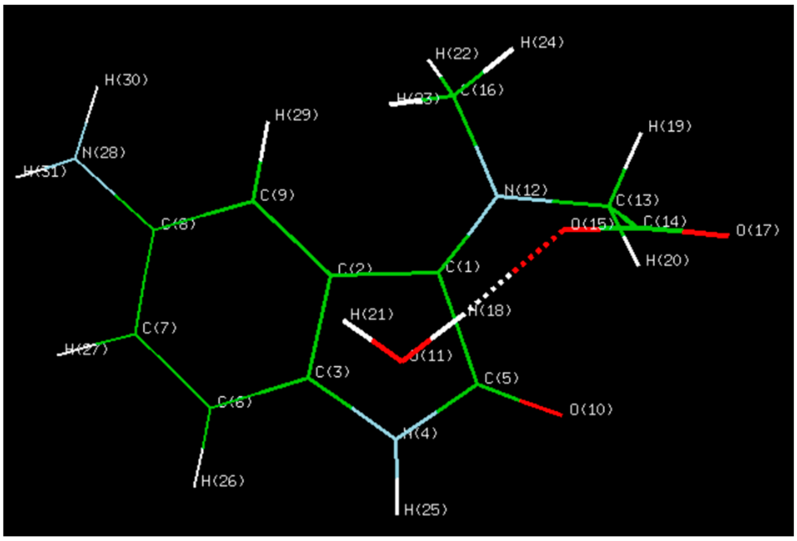

		x	y	z	q
1	C	0.283548	-0.053185	-0.511380	0.220705
2	C	-1.145300	0.036986	-0.263823	-0.023603
3	C	-1.606598	-1.291419	-0.207335	0.101074
4	N	-0.552637	-2.194616	-0.395069	-0.407696
5	C	0.635123	-1.558649	-0.524950	0.296804
6	C	-2.944006	-1.579399	-0.011275	-0.090059
7	C	-3.828130	-0.513664	0.129867	-0.080136
8	C	-3.402382	0.825235	0.065874	0.089932
9	C	-2.044451	1.101325	-0.138562	-0.081785
10	O	1.739160	-2.054383	-0.608146	-0.332559
11	O	0.707178	-0.774297	2.081891	-0.676440
12	N	1.155893	0.876077	-0.702645	-0.205041
13	C	2.560303	0.613801	-1.037138	-0.044285
14	C	3.444944	0.515423	0.234686	0.199762
15	O	2.884489	0.710516	1.344262	-0.459777
16	C	0.816264	2.284333	-0.464707	-0.086127
17	O	4.641267	0.278622	0.005262	-0.499631
18	H	1.514350	-0.254838	1.875169	0.288755
19	H	2.902047	1.456470	-1.640488	0.137430
20	H	2.644581	-0.300769	-1.616400	0.128156
21	H	0.093418	-0.122243	2.442817	0.336390
22	H	0.178696	2.649156	-1.273725	0.135938
23	H	0.309987	2.373856	0.497090	0.133202
24	H	1.741170	2.856048	-0.439626	0.137613
25	H	-0.624384	-3.197864	-0.274751	0.378267
26	H	-3.295027	-2.604603	0.029484	0.130923
27	H	-4.883500	-0.717337	0.289144	0.126291
28	N	-4.309437	1.860525	0.269517	-0.634233
29	H	-1.727374	2.135064	-0.191300	0.126941
30	H	-4.036854	2.740966	-0.150554	0.326688
31	H	-5.266494	1.623805	0.036833	0.326507

TS7a

log file: ageo11_ts2.log
E(RM06-2X) = -891.818859871 A.U.
Zero-point correction= 0.310959
Thermal correction to Energy= 0.331562
Thermal correction to Enthalpy= 0.332506
Thermal correction to Gibbs Free Energy= 0.262067
Sum of electronic and zero-point Energies= -991.203362
Sum of electronic and thermal Energies= -991.182759

Sum of electronic and thermal Enthalpies= -991.181815
Sum of electronic and thermal Free Energies= -991.252255

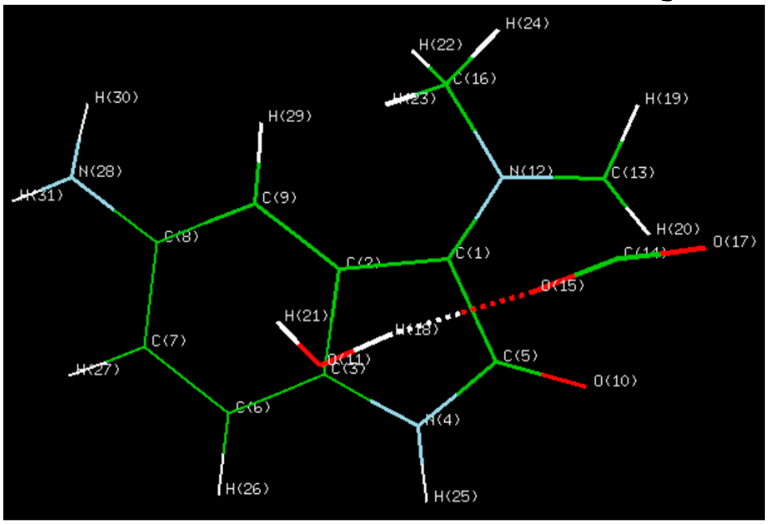

		x	y	z	q
1	C	0.421923	0.096765	-0.722658	0.098601
2	C	-1.004629	0.095618	-0.440054	-0.027638
3	C	-1.375305	-1.267580	-0.396253	0.079022
4	N	-0.257190	-2.066380	-0.633611	-0.419424
5	C	0.864124	-1.313859	-0.838845	0.250450
6	C	-2.675138	-1.664771	-0.139269	-0.103752
7	C	-3.635650	-0.681848	0.083883	-0.113166
8	C	-3.305113	0.682121	0.045020	0.077191
9	C	-1.983602	1.071760	-0.217393	-0.109003
10	O	1.996454	-1.754787	-1.042435	-0.389302
11	O	-0.252018	-0.683141	2.514844	-0.705501
12	N	1.278612	1.116344	-0.808976	-0.211437
13	C	2.599178	0.982630	-1.047226	-0.063756
14	C	3.361369	0.204718	0.766700	0.334052
15	O	2.417115	-0.052051	1.445487	-0.280501
16	C	0.794641	2.454947	-0.431016	-0.086122
17	O	4.536333	0.249909	0.584994	-0.316932
18	H	0.561763	-0.397537	2.076751	0.299378
19	H	3.148178	1.908709	-1.149855	0.131288
20	H	2.917775	0.102729	-1.585808	0.124776
21	H	-0.935538	-0.130623	2.114649	0.312594
22	H	0.084554	2.799030	-1.184134	0.134757
23	H	0.315488	2.393418	0.547325	0.130633
24	H	1.643193	3.133060	-0.389413	0.136574
25	H	-0.246647	-3.077768	-0.625012	0.367450
26	H	-2.937522	-2.717280	-0.108826	0.121691
27	H	-4.662481	-0.969169	0.291202	0.114722
28	N	-4.274698	1.648307	0.331926	-0.643364
29	H	-1.760726	2.131177	-0.243805	0.115520
30	H	-4.089705	2.548685	-0.094461	0.320515
31	H	-5.219264	1.345216	0.125560	0.320697

22a

log file: ageo11_ts2_m2.log
E(RM06-2X) = -891.840221892 A.U.
Zero-point correction= 0.310959
Thermal correction to Energy= 0.331562
Thermal correction to Enthalpy= 0.332506
Thermal correction to Gibbs Free Energy= 0.262067

Sum of electronic and zero-point Energies= -991.203362
Sum of electronic and thermal Energies= -991.182759
Sum of electronic and thermal Enthalpies= -991.181815
Sum of electronic and thermal Free Energies= -991.252255

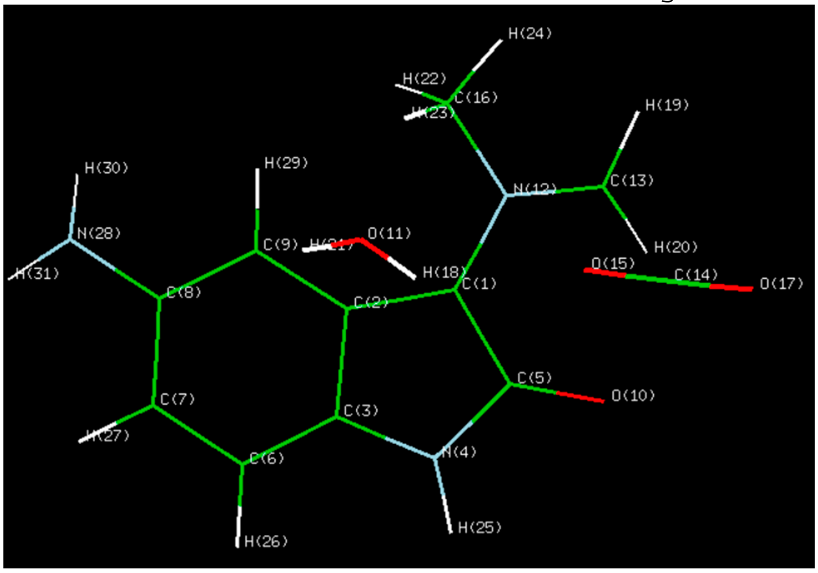

		x	y	z	q
1	C	0.380129	0.076850	-0.762798	0.014565
2	C	-1.025363	-0.030444	-0.454864	-0.034364
3	C	-1.248308	-1.410319	-0.203675	0.062322
4	N	-0.043674	-2.089056	-0.341098	-0.431215
5	C	0.977799	-1.228790	-0.684971	0.206594
6	C	-2.500285	-1.905235	0.122178	-0.116103
7	C	-3.567889	-1.015715	0.215309	-0.139741
8	C	-3.383596	0.356158	-0.023915	0.066763
9	C	-2.115232	0.848919	-0.359646	-0.131188
10	O	2.170667	-1.576440	-0.831268	-0.444221
11	O	-0.190642	0.834582	2.421252	-0.690308
12	N	1.148088	1.214380	-0.967482	-0.210950
13	C	2.341139	1.209588	-1.480629	0.018230
14	C	3.507696	-0.117844	0.951195	0.424304
15	O	2.537633	0.145979	1.539328	-0.214364
16	C	0.587422	2.494917	-0.497692	-0.092726
17	O	4.518066	-0.341542	0.421328	-0.234766
18	H	0.366561	0.269599	1.868227	0.301421
19	H	2.892530	2.140007	-1.506825	0.149330
20	H	2.745591	0.284284	-1.865395	0.137178
21	H	-1.056735	0.774878	1.994791	0.312434
22	H	-0.197452	2.808777	-1.185889	0.130949
23	H	0.185467	2.341182	0.504482	0.116551
24	H	1.380817	3.239381	-0.484419	0.133842
25	H	0.092753	-3.082443	-0.214435	0.357463
26	H	-2.643432	-2.966026	0.305043	0.113920
27	H	-4.557525	-1.379989	0.475238	0.105324
28	N	-4.458477	1.245206	0.135367	-0.650121
29	H	-2.011068	1.911857	-0.546396	0.106974
30	H	-4.367263	2.084272	-0.425994	0.315837
31	H	-5.358662	0.811368	-0.034010	0.316081

1b

log file: r3.log
E(RM06-2X) = -717.310066265 A.U.
Zero-point correction= 0.310959

Thermal correction to Energy= 0.331562
Thermal correction to Enthalpy= 0.332506
Thermal correction to Gibbs Free Energy= 0.262067
Sum of electronic and zero-point Energies= -991.203362
Sum of electronic and thermal Energies= -991.182759
Sum of electronic and thermal Enthalpies= -991.181815
Sum of electronic and thermal Free Energies= -991.252255

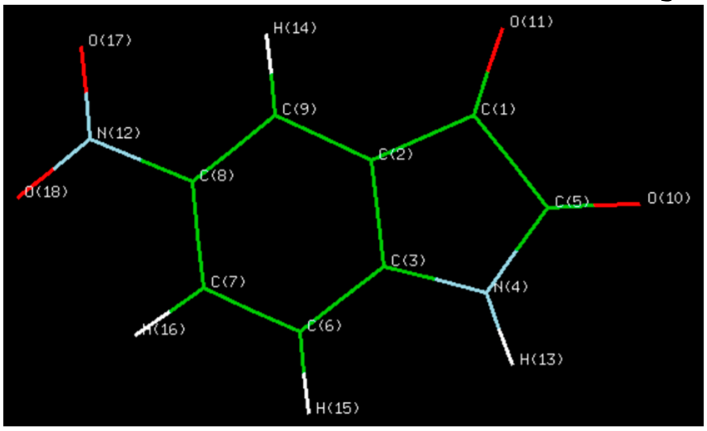

		x	y	z	q
1	C	-1.906732	-1.030498	-0.000026	0.206385
2	C	-0.604425	-0.350381	-0.000009	-0.027020
3	C	-0.863220	1.033722	-0.000013	0.140570
4	N	-2.232792	1.280506	-0.000022	-0.394800
5	C	-2.958775	0.119034	-0.000007	0.287287
6	C	0.168925	1.963666	-0.000003	-0.075713
7	C	1.469547	1.474185	0.000010	-0.042379
8	C	1.708066	0.097907	0.000018	0.071043
9	C	0.685124	-0.845490	0.000010	-0.035856
10	O	-4.159673	-0.016127	0.000024	-0.341077
11	O	-2.194804	-2.201304	0.000007	-0.289090
12	N	3.087891	-0.369562	0.000033	0.094163
13	H	-2.646568	2.206649	0.000040	0.380406
14	H	0.889915	-1.910056	0.000015	0.144707
15	H	-0.029672	3.028968	-0.000003	0.139752
16	H	2.310471	2.157573	0.000019	0.139021
17	O	3.285912	-1.574095	-0.000020	-0.198727
18	O	3.980953	0.462451	-0.000013	-0.198668

21b

log file: ngeo11_ts2_m1.log
E(RM06-2X) = -1040.93319248 A.U.
Zero-point correction= 0.310959
Thermal correction to Energy= 0.331562
Thermal correction to Enthalpy= 0.332506
Thermal correction to Gibbs Free Energy= 0.262067
Sum of electronic and zero-point Energies= -991.203362
Sum of electronic and thermal Energies= -991.182759
Sum of electronic and thermal Enthalpies= -991.181815
Sum of electronic and thermal Free Energies= -991.252255

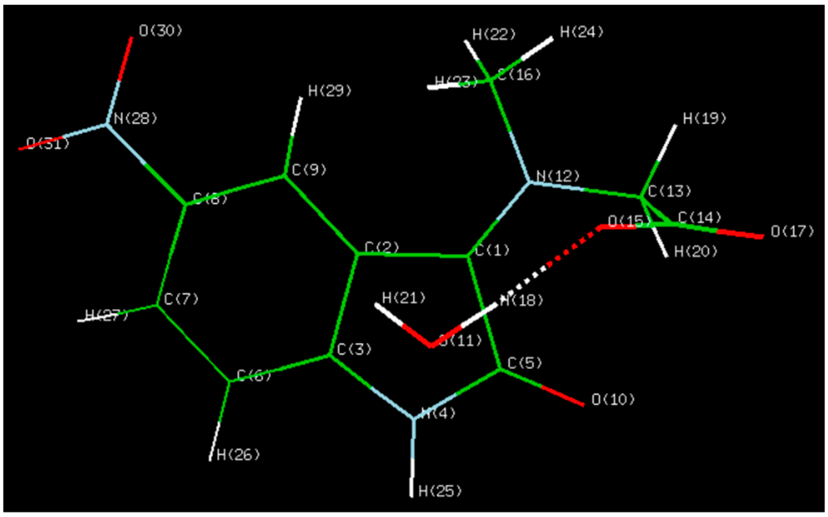

		x	y	z	q
1	C	-0.784922	0.150409	-0.516117	0.225442
2	C	0.647422	0.270185	-0.291711	-0.008376
3	C	0.915135	1.659045	-0.218054	0.147842
4	N	-0.245221	2.388975	-0.374531	-0.393229
5	C	-1.342409	1.586106	-0.509654	0.307992
6	C	2.197012	2.157205	-0.028651	-0.070907
7	C	3.234029	1.241687	0.076011	-0.037018
8	C	2.967312	-0.124471	-0.018198	0.072915
9	C	1.691309	-0.643371	-0.204404	-0.035941
10	O	-2.497308	1.930813	-0.582440	-0.307169
11	O	-1.228532	0.797084	2.042942	-0.668095
12	N	-1.511393	-0.894384	-0.702348	-0.197745
13	C	-2.942422	-0.834736	-1.022564	-0.041664
14	C	-3.817540	-0.845803	0.262467	0.201752
15	O	-3.219650	-0.938867	1.364874	-0.457126
16	C	-0.965681	-2.237734	-0.467361	-0.082254
17	O	-5.036534	-0.786117	0.044419	-0.495007
18	H	-1.980501	0.186662	1.877221	0.290736
19	H	-3.169623	-1.722492	-1.614815	0.140505
20	H	-3.159190	0.053647	-1.608814	0.130828
21	H	-0.562661	0.241947	2.468263	0.340263
22	H	-0.311524	-2.516256	-1.297266	0.140429
23	H	-0.419410	-2.241414	0.476914	0.136665
24	H	-1.799312	-2.933516	-0.406113	0.140841
25	H	-0.314869	3.395781	-0.272882	0.386772
26	H	2.379458	3.223490	0.031216	0.143321
27	H	4.253091	1.578489	0.224474	0.142180
28	N	4.080747	-1.060574	0.084926	0.095426
29	H	1.556855	-1.713888	-0.270882	0.140894
30	O	3.840140	-2.253644	-0.006847	-0.196107
31	O	5.199791	-0.606982	0.257770	-0.194175

TS7b

```
log file: ngeo11_ts2.log
E(RM06-2X) =  -1040.90802803     A.U.
Zero-point correction=                          0.310959
Thermal correction to Energy=                   0.331562
Thermal correction to Enthalpy=                 0.332506
Thermal correction to Gibbs Free Energy=        0.262067
Sum of electronic and zero-point Energies=         -991.203362
Sum of electronic and thermal Energies=            -991.182759
```

Sum of electronic and thermal Enthalpies= -991.181815
Sum of electronic and thermal Free Energies= -991.252255

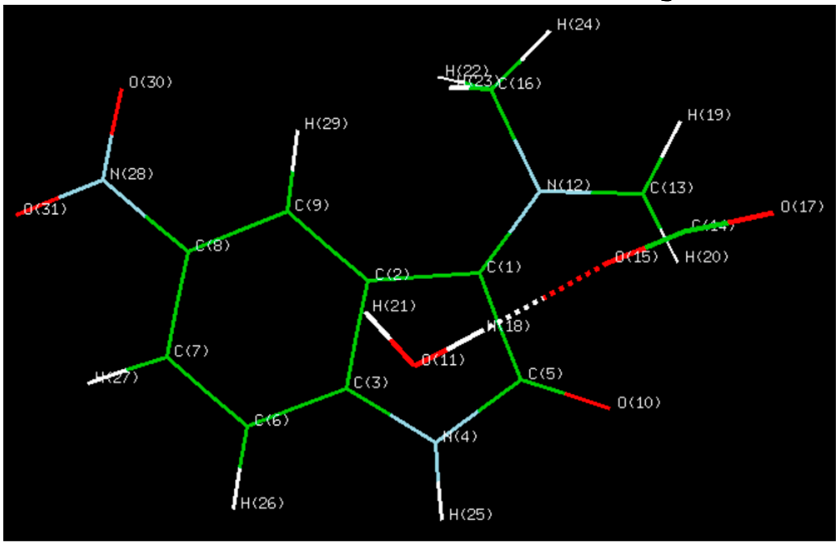

		x	y	z	q
1	C	-0.896066	-0.000933	-0.716097	0.105454
2	C	0.526288	0.161290	-0.471833	-0.013872
3	C	0.752582	1.566413	-0.458063	0.126809
4	N	-0.431312	2.226115	-0.682216	-0.404824
5	C	-1.487449	1.350897	-0.817478	0.262546
6	C	2.005191	2.126006	-0.240200	-0.086005
7	C	3.074346	1.268634	-0.026452	-0.067879
8	C	2.853853	-0.107946	-0.039832	0.060942
9	C	1.605245	-0.689361	-0.256585	-0.066700
10	O	-2.657638	1.678347	-0.976978	-0.366628
11	O	-0.441243	1.265913	2.345081	-0.694614
12	N	-1.620310	-1.112448	-0.845520	-0.206094
13	C	-2.959218	-1.130178	-1.015307	-0.053694
14	C	-3.724735	-0.642356	0.840804	0.332761
15	O	-2.821479	-0.192373	1.476857	-0.282307
16	C	-0.952459	-2.403090	-0.603707	-0.082870
17	O	-4.872907	-0.944438	0.741295	-0.318793
18	H	-1.257340	0.795656	2.125588	0.305016
19	H	-3.386136	-2.107077	-1.196753	0.135789
20	H	-3.409858	-0.255807	-1.463693	0.129616
21	H	0.246955	0.601015	2.218169	0.326965
22	H	-0.175893	-2.543800	-1.356646	0.137506
23	H	-0.521396	-2.394638	0.398972	0.134899
24	H	-1.690359	-3.196813	-0.685069	0.139513
25	H	-0.552537	3.231641	-0.660739	0.376043
26	H	2.138384	3.201662	-0.233803	0.134108
27	H	4.070583	1.654469	0.149995	0.131558
28	N	3.986583	-0.992007	0.187948	0.090604
29	H	1.522118	-1.766262	-0.244762	0.128445
30	O	3.794688	-2.199435	0.175721	-0.207982
31	O	5.084238	-0.491507	0.382496	-0.206316

22b

log file: ngeo11_ts2_m2.log
E(RM06-2X) = -1040.93352134 A.U.
Zero-point correction= 0.310959
Thermal correction to Energy= 0.331562
Thermal correction to Enthalpy= 0.332506

```
Thermal correction to Gibbs Free Energy=        0.262067
Sum of electronic and zero-point Energies=         -991.203362
Sum of electronic and thermal Energies=            -991.182759
Sum of electronic and thermal Enthalpies=          -991.181815
Sum of electronic and thermal Free Energies=       -991.252255
```

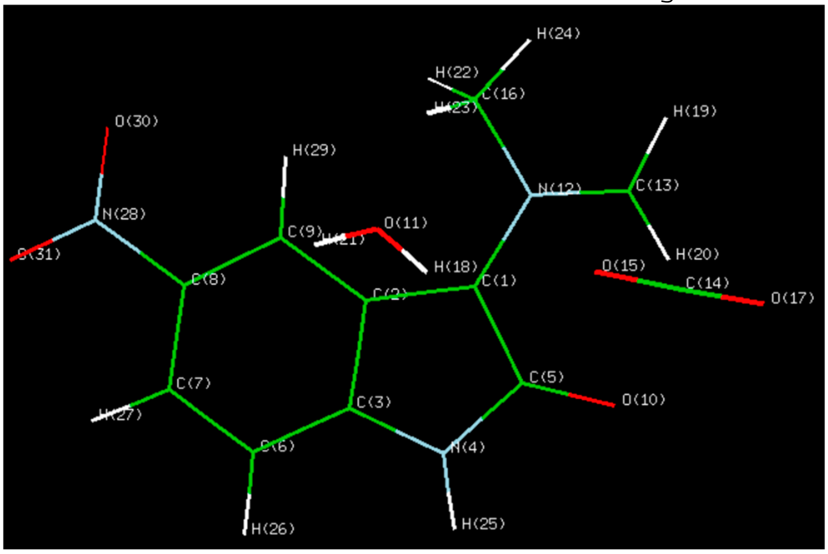

		x	y	z	q
1	C	-0.850995	-0.019532	-0.749276	0.020183
2	C	0.535945	0.227456	-0.460975	-0.021481
3	C	0.630238	1.634365	-0.219223	0.108829
4	N	-0.619055	2.180941	-0.341182	-0.413253
5	C	-1.568616	1.217048	-0.673990	0.215310
6	C	1.828185	2.271746	0.085915	-0.098519
7	C	2.980985	1.504543	0.166871	-0.095180
8	C	2.890584	0.130740	-0.063855	0.052967
9	C	1.702348	-0.531125	-0.376703	-0.088753
10	O	-2.781678	1.460887	-0.813450	-0.427083
11	O	-0.301867	-0.750381	2.435621	-0.682547
12	N	-1.499288	-1.233323	-0.954782	-0.206821
13	C	-2.679629	-1.341795	-1.477976	0.038498
14	C	-4.028160	-0.123979	0.937962	0.426085
15	O	-3.044556	-0.316406	1.531182	-0.213886
16	C	-0.811664	-2.448140	-0.476635	-0.090577
17	O	-5.043322	0.027739	0.392719	-0.232115
18	H	-0.860165	-0.204028	1.865665	0.303551
19	H	-3.138436	-2.321395	-1.513406	0.156088
20	H	-3.169350	-0.459177	-1.866037	0.143250
21	H	0.596428	-0.499058	2.183970	0.326246
22	H	-0.002543	-2.685997	-1.167397	0.134164
23	H	-0.427531	-2.247464	0.524501	0.117183
24	H	-1.526343	-3.268366	-0.456273	0.136423
25	H	-0.846998	3.160493	-0.227354	0.366744
26	H	1.854677	3.341802	0.260427	0.126474
27	H	3.936165	1.953955	0.405156	0.122331
28	N	4.105922	-0.660884	0.022940	0.087331
29	H	1.723664	-1.598619	-0.545049	0.120572
30	O	4.036627	-1.866715	-0.175915	-0.216405
31	O	5.156303	-0.093530	0.291628	-0.215594

1c

log file: r4.log

E(RM06-2X) = -717.004275886   A.U.

Zero-point correction= 0.310959
Thermal correction to Energy= 0.331562
Thermal correction to Enthalpy= 0.332506
Thermal correction to Gibbs Free Energy= 0.262067
Sum of electronic and zero-point Energies= -991.203362
Sum of electronic and thermal Energies= -991.182759
Sum of electronic and thermal Enthalpies= -991.181815
Sum of electronic and thermal Free Energies= -991.252255

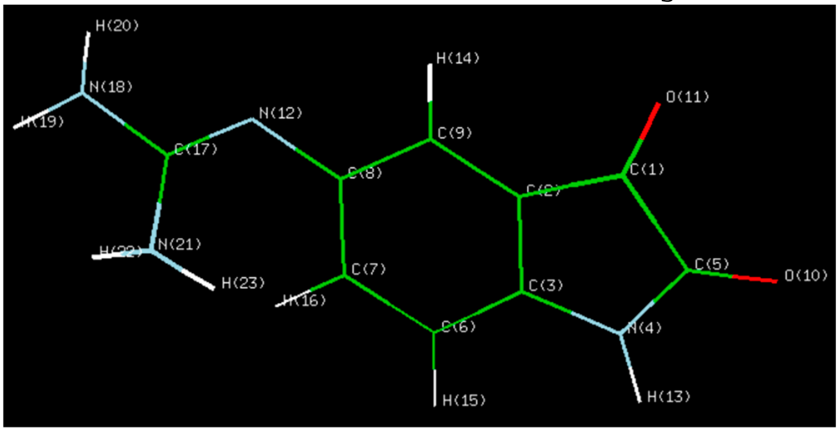

		x	y	z	q
1	C	-2.394837	-1.048045	0.087420	0.195914
2	C	-1.087847	-0.411388	-0.100225	-0.045253
3	C	-1.312491	0.971499	-0.150523	0.097898
4	N	-2.685472	1.260383	-0.004478	-0.408147
5	C	-3.420457	0.127286	0.142292	0.274343
6	C	-0.259790	1.848710	-0.332594	-0.098030
7	C	1.021600	1.304517	-0.457184	-0.082469
8	C	1.276238	-0.081302	-0.397303	0.077895
9	C	0.185103	-0.947645	-0.225454	-0.077287
10	O	-4.620037	0.010225	0.288428	-0.368943
11	O	-2.713924	-2.209736	0.188954	-0.309243
12	N	2.547253	-0.631039	-0.604069	-0.455558
13	H	-3.078932	2.193852	-0.014872	0.372078
14	H	0.342149	-2.021767	-0.193821	0.121327
15	H	-0.418239	2.920511	-0.386554	0.125704
16	H	1.858608	1.977699	-0.619268	0.116144
17	C	3.554529	-0.246446	0.113847	0.351302
18	N	4.780005	-0.826472	-0.099204	-0.618092
19	H	5.591202	-0.274276	0.147437	0.339679
20	H	4.858904	-1.304876	-0.986763	0.333550
21	N	3.535890	0.693706	1.113448	-0.612824
22	H	4.272754	0.620154	1.803466	0.342602
23	H	2.629216	0.905623	1.509783	0.327410

21c

log file: g2geo11_ts2_m1.log
E(RM06-2X) = -1040.63087975 A.U.
Zero-point correction= 0.310959
Thermal correction to Energy= 0.331562
Thermal correction to Enthalpy= 0.332506
Thermal correction to Gibbs Free Energy= 0.262067
Sum of electronic and zero-point Energies= -991.203362
Sum of electronic and thermal Energies= -991.182759
Sum of electronic and thermal Enthalpies= -991.181815
Sum of electronic and thermal Free Energies= -991.252255

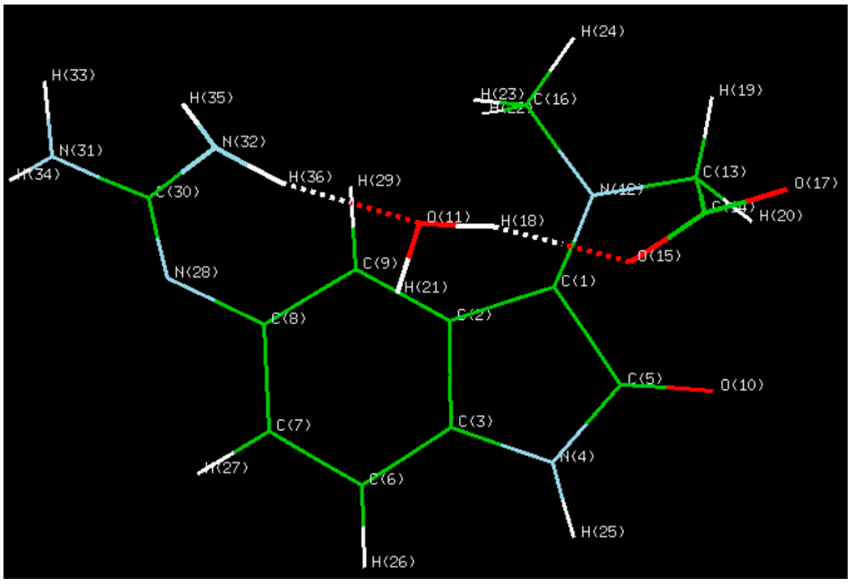

		x	y	z	q
1	C	-1.253824	0.385062	-0.749271	0.213631
2	C	0.104138	0.849480	-0.570508	-0.024812
3	C	0.002967	2.105089	0.059435	0.112297
4	N	-1.336352	2.487499	0.182412	-0.402573
5	C	-2.179349	1.522220	-0.262098	0.296951
6	C	1.123478	2.778788	0.511773	-0.091034
7	C	2.357525	2.151750	0.363291	-0.070372
8	C	2.498390	0.874126	-0.216422	0.081170
9	C	1.356129	0.241877	-0.721756	-0.073915
10	O	-3.390124	1.569057	-0.307844	-0.329031
11	O	0.263661	-1.343040	1.732413	-0.670332
12	N	-1.699916	-0.749817	-1.181382	-0.209703
13	C	-3.079684	-1.150586	-0.920697	-0.042831
14	C	-3.360212	-1.237870	0.616863	0.203052
15	O	-2.474626	-0.810013	1.397504	-0.437901
16	C	-0.771168	-1.784780	-1.650277	-0.084726
17	O	-4.471987	-1.716455	0.888767	-0.492804
18	H	-0.689821	-1.261016	1.530320	0.293724
19	H	-3.246107	-2.122897	-1.378863	0.136455
20	H	-3.772681	-0.424941	-1.343502	0.131765
21	H	0.519391	-0.438888	1.958782	0.332010
22	H	-0.122327	-1.364830	-2.418953	0.135158
23	H	-0.180775	-2.144289	-0.801145	0.128418
24	H	-1.349268	-2.602391	-2.073268	0.138272
25	H	-1.660877	3.358134	0.586512	0.379795
26	H	1.038471	3.749061	0.988398	0.130686
27	H	3.255438	2.646316	0.721274	0.120386
28	N	3.779286	0.328478	-0.318350	-0.458338
29	H	1.468096	-0.716812	-1.207833	0.120564
30	C	3.992500	-0.907705	0.023547	0.351618
31	N	5.276232	-1.388283	-0.051077	-0.618161
32	N	3.078148	-1.812423	0.487899	-0.615163
33	H	5.381371	-2.385338	-0.187383	0.339790
34	H	5.893902	-0.823311	-0.618413	0.332938
35	H	3.449680	-2.599957	1.002587	0.342327
36	H	2.172990	-1.488126	0.814985	0.300713

TS7c

log file: g2geo11_ts2.log

E(RM06-2X) = -1040.60307828 A.U.
Zero-point correction= 0.310959
Thermal correction to Energy= 0.331562
Thermal correction to Enthalpy= 0.332506
Thermal correction to Gibbs Free Energy= 0.262067
Sum of electronic and zero-point Energies= -991.203362
Sum of electronic and thermal Energies= -991.182759
Sum of electronic and thermal Enthalpies= -991.181815
Sum of electronic and thermal Free Energies= -991.252255

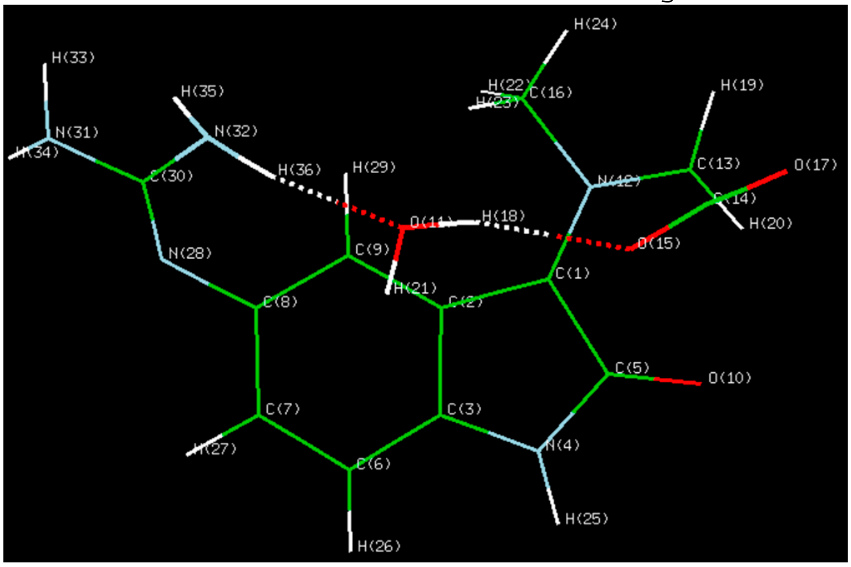

		x	y	z	q
1	C	-1.269430	0.457335	-0.772253	0.095678
2	C	0.113370	0.829045	-0.525776	-0.029826
3	C	0.059540	2.093164	0.102207	0.085525
4	N	-1.267482	2.504816	0.208467	-0.418585
5	C	-2.128269	1.559923	-0.281641	0.249271
6	C	1.201523	2.745041	0.539670	-0.106972
7	C	2.427090	2.107219	0.367549	-0.109493
8	C	2.523242	0.841650	-0.238009	0.068083
9	C	1.359445	0.223829	-0.719334	-0.103926
10	O	-3.357107	1.637856	-0.263321	-0.391005
11	O	0.365168	-1.216079	1.897001	-0.658087
12	N	-1.775645	-0.693156	-1.220791	-0.213133
13	C	-3.091478	-0.983407	-1.257229	-0.064087
14	C	-3.525664	-1.304278	0.807123	0.338009
15	O	-2.592559	-0.868729	1.406469	-0.276678
16	C	-0.839016	-1.799849	-1.482967	-0.087642
17	O	-4.594383	-1.823635	0.762280	-0.313752
18	H	-0.557446	-1.237921	1.600234	0.310944
19	H	-3.347204	-1.933469	-1.705835	0.130485
20	H	-3.782585	-0.155616	-1.309343	0.123996
21	H	0.583169	-0.273973	1.877335	0.328866
22	H	-0.205977	-1.534904	-2.330474	0.134485
23	H	-0.233196	-1.962999	-0.589193	0.124872
24	H	-1.412739	-2.692908	-1.716385	0.135678
25	H	-1.583503	3.358865	0.649418	0.367036
26	H	1.136229	3.717439	1.017017	0.120081
27	H	3.339892	2.582605	0.712768	0.107403
28	N	3.795637	0.271727	-0.399325	-0.459963
29	H	1.458325	-0.729758	-1.220134	0.109659
30	C	4.010779	-0.951286	-0.028112	0.345759
31	N	5.286255	-1.457772	-0.145777	-0.623845

32	N	3.107337	-1.832979	0.507686	-0.620960
33	H	5.356880	-2.457473	-0.287820	0.336199
34	H	5.877297	-0.917464	-0.763840	0.329672
35	H	3.500926	-2.586463	1.056220	0.338470
36	H	2.231486	-1.470031	0.871411	0.297767

22c

log file: g2geo11_ts2_m2.log
E(RM06-2X) = -1040.62208424 A.U.
Zero-point correction= 0.310959
Thermal correction to Energy= 0.331562
Thermal correction to Enthalpy= 0.332506
Thermal correction to Gibbs Free Energy= 0.262067
Sum of electronic and zero-point Energies= -991.203362
Sum of electronic and thermal Energies= -991.182759
Sum of electronic and thermal Enthalpies= -991.181815
Sum of electronic and thermal Free Energies= -991.252255

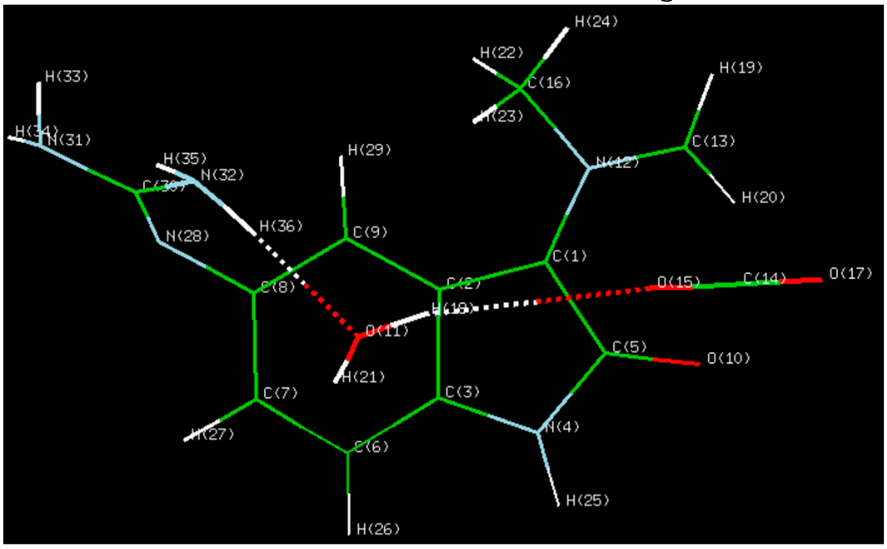

		x	y	z	q
1	C	-1.277459	0.124335	-0.896665	0.011600
2	C	0.083289	0.559039	-0.693557	-0.036364
3	C	-0.016690	1.889313	-0.201439	0.071974
4	N	-1.357357	2.230821	-0.115376	-0.429760
5	C	-2.167172	1.183131	-0.508304	0.205970
6	C	1.100818	2.639076	0.133495	-0.112437
7	C	2.359278	2.051732	-0.004621	-0.128212
8	C	2.498505	0.739231	-0.486694	0.060674
9	C	1.360982	0.007283	-0.855428	-0.125082
10	O	-3.415896	1.204621	-0.451500	-0.443270
11	O	0.953659	-0.079749	2.334763	-0.653487
12	N	-1.751297	-1.142088	-1.201573	-0.210556
13	C	-2.979117	-1.406477	-1.536908	0.014624
14	C	-3.900616	-0.809378	1.286775	0.421539
15	O	-2.796448	-0.823391	1.655896	-0.225595
16	C	-0.802003	-2.263464	-1.074974	-0.088082
17	O	-5.019305	-0.844095	0.969273	-0.237155
18	H	0.136096	-0.260734	1.850482	0.327025
19	H	-3.264291	-2.441656	-1.669172	0.148429
20	H	-3.673082	-0.590778	-1.678933	0.136112
21	H	1.235521	0.778588	1.984036	0.313781
22	H	-0.114343	-2.238779	-1.920763	0.132942
23	H	-0.253750	-2.155149	-0.138515	0.127126

24	H	-1.358963	-3.197854	-1.082492	0.134899
25	H	-1.723556	3.103122	0.241018	0.358146
26	H	0.993562	3.652612	0.508205	0.114725
27	H	3.253180	2.605714	0.264060	0.100993
28	N	3.791389	0.194839	-0.638694	-0.464471
29	H	1.504931	-0.995132	-1.240469	0.101789
30	C	4.111653	-0.831202	0.080705	0.341475
31	N	5.389930	-1.342260	-0.014241	-0.627889
32	N	3.303694	-1.488100	0.975291	-0.628247
33	H	5.480859	-2.334238	0.166095	0.333908
34	H	5.876902	-1.040873	-0.848294	0.327796
35	H	3.784943	-2.020240	1.688738	0.334174
36	H	2.482608	-0.991894	1.316384	0.290898

1d

log file: r5.log
E(RM06-2X) = -717.475875954 A.U.
Zero-point correction= 0.310959
Thermal correction to Energy= 0.331562
Thermal correction to Enthalpy= 0.332506
Thermal correction to Gibbs Free Energy= 0.262067
Sum of electronic and zero-point Energies= -991.203362
Sum of electronic and thermal Energies= -991.182759
Sum of electronic and thermal Enthalpies= -991.181815
Sum of electronic and thermal Free Energies= -991.252255

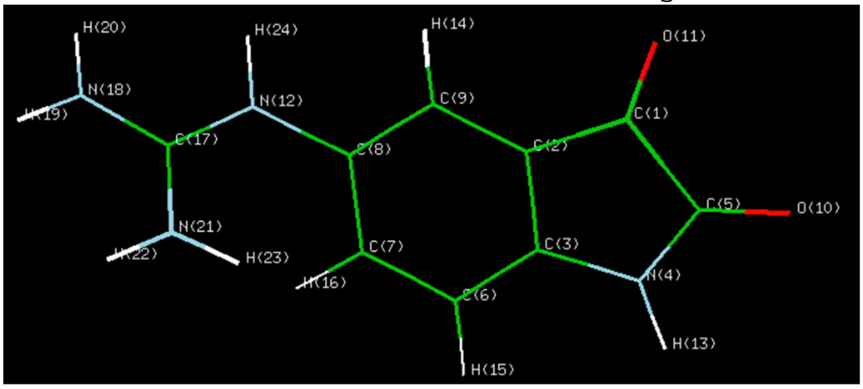

		x	y	z	q
1	C	-2.412429	-1.057561	0.087368	0.202411
2	C	-1.110555	-0.408375	-0.110286	-0.033235
3	C	-1.345726	0.974473	-0.154493	0.121502
4	N	-2.709934	1.252839	0.003516	-0.401832
5	C	-3.442111	0.111713	0.154141	0.281714
6	C	-0.305818	1.871310	-0.340257	-0.082633
7	C	0.976074	1.344032	-0.490047	-0.058068
8	C	1.216322	-0.034373	-0.422581	0.090545
9	C	0.167111	-0.931475	-0.230676	-0.050929
10	O	-4.637077	-0.006415	0.309934	-0.352716
11	O	-2.716805	-2.221180	0.187284	-0.296469
12	N	2.538852	-0.535839	-0.594714	-0.403024
13	H	-3.107753	2.185229	0.000306	0.376816
14	H	0.343969	-2.001731	-0.181635	0.141113
15	H	-0.479962	2.940219	-0.387035	0.135271
16	H	1.810221	2.016043	-0.668043	0.133183
17	C	3.586724	-0.219253	0.182106	0.428300
18	N	4.785430	-0.698184	-0.127318	-0.550667
19	H	5.582094	-0.512597	0.466807	0.377947
20	H	4.929274	-1.221932	-0.979985	0.377492

21	N	3.435197	0.575558	1.235049	-0.553173
22	H	4.233195	0.806651	1.811618	0.378240
23	H	2.514241	0.807841	1.581562	0.359598
24	H	2.681406	-1.262520	-1.288707	0.378612

21d

log file: p1geo11_ts2_m1.log
E(RM06-2X) = -1041.10417335 A.U.
Zero-point correction= 0.310959
Thermal correction to Energy= 0.331562
Thermal correction to Enthalpy= 0.332506
Thermal correction to Gibbs Free Energy= 0.262067
Sum of electronic and zero-point Energies= -991.203362
Sum of electronic and thermal Energies= -991.182759
Sum of electronic and thermal Enthalpies= -991.181815
Sum of electronic and thermal Free Energies= -991.252255

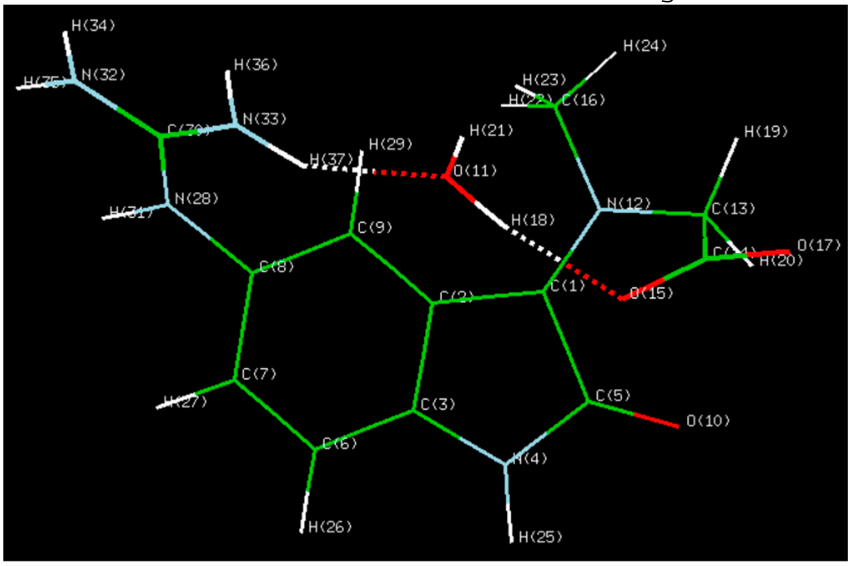

		x	y	z	q
1	C	-1.326097	0.455612	-0.714122	0.215896
2	C	0.043528	0.898203	-0.552188	-0.016805
3	C	-0.022580	2.124975	0.140466	0.131794
4	N	-1.344587	2.504441	0.338031	-0.396905
5	C	-2.217576	1.559768	-0.109898	0.300867
6	C	1.115546	2.781830	0.580574	-0.077358
7	C	2.337000	2.149429	0.382368	-0.047654
8	C	2.415994	0.908034	-0.261894	0.092847
9	C	1.280597	0.284353	-0.764524	-0.053145
10	O	-3.426066	1.617415	-0.093384	-0.319795
11	O	0.240959	-1.808304	1.107162	-0.632868
12	N	-1.803628	-0.624500	-1.239059	-0.208440
13	C	-3.145731	-1.070741	-0.879365	-0.039707
14	C	-3.270647	-1.254534	0.673824	0.208380
15	O	-2.346800	-0.795247	1.392950	-0.429409
16	C	-0.933818	-1.589284	-1.921794	-0.087964
17	O	-4.313959	-1.825891	1.019561	-0.484152
18	H	-0.629728	-1.384612	1.255391	0.299522
19	H	-3.336589	-2.016400	-1.381944	0.138257
20	H	-3.890485	-0.336314	-1.184029	0.134959
21	H	0.163788	-2.668786	1.539777	0.355359
22	H	-0.261451	-1.056125	-2.593140	0.134488
23	H	-0.374644	-2.149845	-1.166284	0.124154
24	H	-1.563071	-2.260595	-2.501571	0.138641

25	H	-1.643501	3.348801	0.812812	0.383667
26	H	1.049937	3.730073	1.101106	0.139261
27	H	3.248924	2.610358	0.749225	0.140097
28	N	3.682612	0.276879	-0.393581	-0.405856
29	H	1.378543	-0.663358	-1.278025	0.134958
30	C	3.980559	-0.933154	0.118316	0.424308
31	H	4.463153	0.849655	-0.696967	0.377604
32	N	5.222822	-1.387108	-0.017821	-0.554651
33	N	3.066769	-1.672208	0.729573	-0.563109
34	H	5.492734	-2.271487	0.390487	0.376457
35	H	5.929865	-0.833825	-0.482399	0.375991
36	H	3.331979	-2.587359	1.070065	0.372918
37	H	2.098913	-1.383426	0.884606	0.317417

TS7d

log file: p3geo11_ts2.log

E(RM06-2X) = -1041.07916376 A.U.

Zero-point correction= 0.310959

Thermal correction to Energy= 0.331562

Thermal correction to Enthalpy= 0.332506

Thermal correction to Gibbs Free Energy= 0.262067

Sum of electronic and zero-point Energies= -991.203362

Sum of electronic and thermal Energies= -991.182759

Sum of electronic and thermal Enthalpies= -991.181815

Sum of electronic and thermal Free Energies= -991.252255

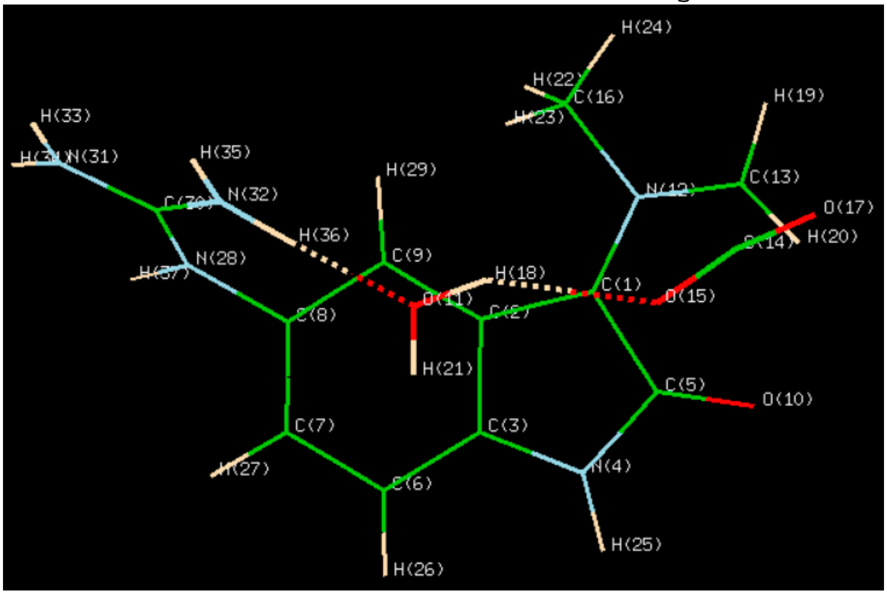

		x	y	z	q
1	C	-1.353247	0.501189	-0.779116	0.102752
2	C	0.039479	0.850881	-0.569117	-0.019670
3	C	0.023005	2.085494	0.124766	0.107856
4	N	-1.287283	2.502211	0.297774	-0.411434
5	C	-2.180499	1.581961	-0.192559	0.257171
6	C	1.179667	2.706288	0.571003	-0.091749
7	C	2.392570	2.058010	0.356460	-0.082998
8	C	2.427330	0.840377	-0.325944	0.080314
9	C	1.270583	0.243961	-0.823634	-0.082598
10	O	-3.402353	1.669118	-0.113377	-0.377605
11	O	0.498255	-1.097619	1.875473	-0.635435
12	N	-1.882437	-0.617997	-1.271275	-0.210732
13	C	-3.193957	-0.931437	-1.200794	-0.056218
14	C	-3.405669	-1.423551	0.818140	0.332365

15	O	-2.467740	-0.945659	1.382040	-0.283106
16	C	-0.961503	-1.687145	-1.692400	-0.083816
17	O	-4.426823	-2.037141	0.838784	-0.319699
18	H	-0.362025	-1.239385	1.451753	0.318131
19	H	-3.479348	-1.841663	-1.710528	0.133797
20	H	-3.896061	-0.113354	-1.128948	0.127663
21	H	0.518452	-0.142804	2.031195	0.340825
22	H	-0.344852	-1.319125	-2.513009	0.136385
23	H	-0.339427	-1.967038	-0.838544	0.132636
24	H	-1.546201	-2.540707	-2.024700	0.138565
25	H	-1.575530	3.333214	0.799135	0.372089
26	H	1.133847	3.651332	1.100886	0.129346
27	H	3.317034	2.489469	0.726381	0.127607
28	N	3.676563	0.176398	-0.506711	-0.408317
29	H	1.363875	-0.686443	-1.367364	0.124015
30	C	3.962804	-1.030209	0.012832	0.421087
31	N	5.176127	-1.538555	-0.188972	-0.557930
32	N	3.059481	-1.717278	0.699012	-0.567202
33	H	5.429349	-2.432982	0.207186	0.374713
34	H	5.851989	-1.056056	-0.765028	0.374277
35	H	3.316374	-2.624557	1.065349	0.369837
36	H	2.148609	-1.340086	0.976018	0.312533
37	H	4.452672	0.712246	-0.879758	0.374546

22d

log file: p1geo11_ts2_m2.log
E(RM06-2X) = -1041.10185752 A.U.
Zero-point correction= 0.310959
Thermal correction to Energy= 0.331562
Thermal correction to Enthalpy= 0.332506
Thermal correction to Gibbs Free Energy= 0.262067
Sum of electronic and zero-point Energies= -991.203362
Sum of electronic and thermal Energies= -991.182759
Sum of electronic and thermal Enthalpies= -991.181815
Sum of electronic and thermal Free Energies= -991.252255

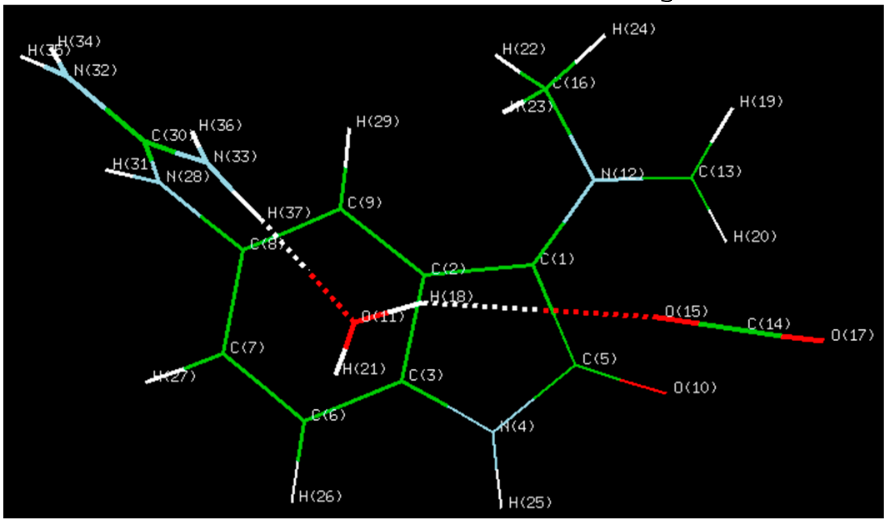

		x	y	z	q
1	C	-1.339594	0.087303	-0.891963	0.014932
2	C	0.027203	0.505108	-0.717704	-0.027762
3	C	-0.044121	1.848004	-0.240187	0.092681
4	N	-1.370256	2.204922	-0.131976	-0.422225
5	C	-2.205227	1.162176	-0.499056	0.211190
6	C	1.081642	2.601264	0.063431	-0.100651
7	C	2.333597	2.006370	-0.084211	-0.105622

8	C	2.423896	0.696999	-0.559331	0.069238
9	C	1.297964	-0.055163	-0.893098	-0.104086
10	O	-3.449579	1.210013	-0.424660	-0.432765
11	O	1.069136	-0.034353	2.339330	-0.629910
12	N	-1.837993	-1.166422	-1.219954	-0.208423
13	C	-3.082213	-1.413306	-1.498654	0.024289
14	C	-3.944708	-0.767404	1.357906	0.423166
15	O	-2.827810	-0.816894	1.683310	-0.227100
16	C	-0.886014	-2.291446	-1.202313	-0.086106
17	O	-5.073857	-0.762395	1.079311	-0.234792
18	H	0.244522	-0.372797	1.963675	0.341319
19	H	-3.373629	-2.440263	-1.674749	0.151839
20	H	-3.782517	-0.592022	-1.556323	0.138238
21	H	1.082380	0.895821	2.069388	0.326391
22	H	-0.212159	-2.192467	-2.053879	0.134238
23	H	-0.326778	-2.262681	-0.266755	0.129471
24	H	-1.442603	-3.222401	-1.281246	0.136984
25	H	-1.717116	3.091038	0.211162	0.362949
26	H	0.982641	3.619579	0.425040	0.122624
27	H	3.238065	2.548559	0.170407	0.117940
28	N	3.712798	0.083967	-0.678309	-0.410682
29	H	1.445160	-1.062390	-1.262714	0.115176
30	C	4.157922	-0.846137	0.178081	0.417681
31	H	4.360465	0.426964	-1.378777	0.372106
32	N	5.356882	-1.391500	-0.022666	-0.561919
33	N	3.407213	-1.234397	1.200857	-0.571968
34	H	5.731726	-2.062089	0.633127	0.372847
35	H	5.910584	-1.140609	-0.829589	0.372367
36	H	3.772400	-1.932451	1.834436	0.367234
37	H	2.541150	-0.751341	1.465404	0.309071

32

log file: geo15_ts3_m1.log
E(RM06-2X) = -1297.38234485 A.U.
Zero-point correction= 0.310959
Thermal correction to Energy= 0.331562
Thermal correction to Enthalpy= 0.332506
Thermal correction to Gibbs Free Energy= 0.262067
Sum of electronic and zero-point Energies= -991.203362
Sum of electronic and thermal Energies= -991.182759
Sum of electronic and thermal Enthalpies= -991.181815
Sum of electronic and thermal Free Energies= -991.252255

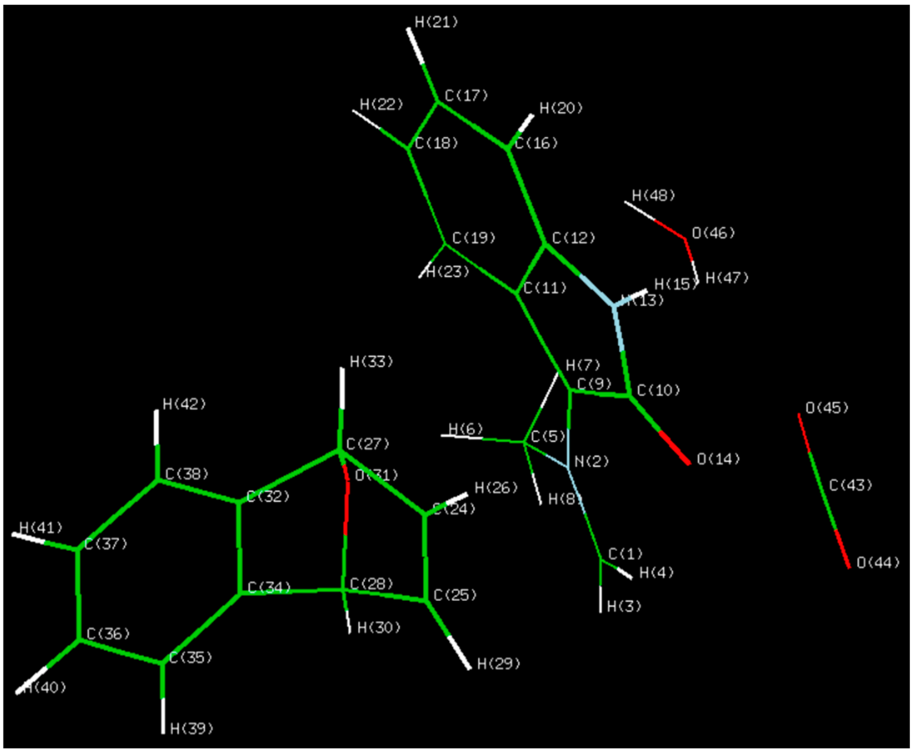

		x	y	z	q
1	C	-1.193127	-2.298806	0.682189	0.011584
2	N	-1.270944	-1.037285	0.987224	-0.214227
3	H	-1.130472	-3.016781	1.489450	0.146596
4	H	-1.216781	-2.592609	-0.356476	0.129722
5	C	-1.203436	-0.650800	2.406213	-0.098305
6	H	-0.237714	-0.178016	2.584700	0.119499
7	H	-2.025432	0.036709	2.609685	0.114825
8	H	-1.298237	-1.547146	3.016078	0.131227
9	C	-1.416431	-0.019571	0.062379	0.031798
10	C	-1.800551	-0.270118	-1.298928	0.212315
11	C	-1.223008	1.399534	0.237682	-0.025791
12	C	-1.500384	1.979446	-1.032262	0.082043
13	N	-1.849590	0.970089	-1.910991	-0.427093
14	O	-2.084205	-1.340994	-1.877794	-0.437889
15	H	-2.098915	1.093617	-2.882986	0.359171
16	C	-1.400124	3.341511	-1.276275	-0.117395
17	C	-1.014619	4.170652	-0.223512	-0.125741
18	C	-0.735780	3.630430	1.034436	-0.125899
19	C	-0.831843	2.259457	1.276427	-0.110890
20	H	-1.617518	3.740256	-2.262485	0.113682
21	H	-0.931088	5.240448	-0.385754	0.104169
22	H	-0.434077	4.286373	1.845197	0.103175
23	H	-0.599915	1.884321	2.265512	0.106341
24	C	1.340064	-0.730401	-1.204628	-0.101852
25	C	1.544450	-1.861165	-0.527223	-0.105323
26	H	0.966212	-0.591850	-2.211360	0.117534
27	C	1.889682	0.388756	-0.305389	0.024434
28	C	2.207495	-1.430448	0.791582	0.026375
29	H	1.390832	-2.886461	-0.837978	0.120083
30	H	2.091036	-2.078397	1.659009	0.126027
31	O	1.581971	-0.148087	0.996088	-0.264728
32	C	3.415493	0.224441	-0.287496	-0.028500
33	H	1.489154	1.393479	-0.432831	0.115312
34	C	3.623824	-0.963296	0.430999	-0.028828

35	C	4.891281	-1.458459	0.646554	-0.104659
36	C	5.975660	-0.727342	0.126751	-0.109928
37	C	5.769735	0.448180	-0.584450	-0.109833
38	C	4.470802	0.942541	-0.805875	-0.104205
39	H	5.058315	-2.376881	1.201813	0.111550
40	H	6.987614	-1.086629	0.287472	0.107821
41	H	6.622778	0.996469	-0.972586	0.107851
42	H	4.315896	1.862529	-1.362026	0.111878
43	C	-4.026643	-2.426307	-0.400785	0.425901
44	O	-3.879141	-3.460407	-0.911122	-0.233920
45	O	-4.234709	-1.420543	0.147926	-0.213922
46	O	-4.008175	1.135570	1.672800	-0.689007
47	H	-3.779793	0.587217	0.909113	0.300548
48	H	-3.409307	1.890004	1.589292	0.316489

TS13

log file: geo15_ts3.log
E(RM06-2X) = -1297.37152273 A.U.
Zero-point correction= 0.310959
Thermal correction to Energy= 0.331562
Thermal correction to Enthalpy= 0.332506
Thermal correction to Gibbs Free Energy= 0.262067
Sum of electronic and zero-point Energies= -991.203362
Sum of electronic and thermal Energies= -991.182759
Sum of electronic and thermal Enthalpies= -991.181815
Sum of electronic and thermal Free Energies= -991.252255

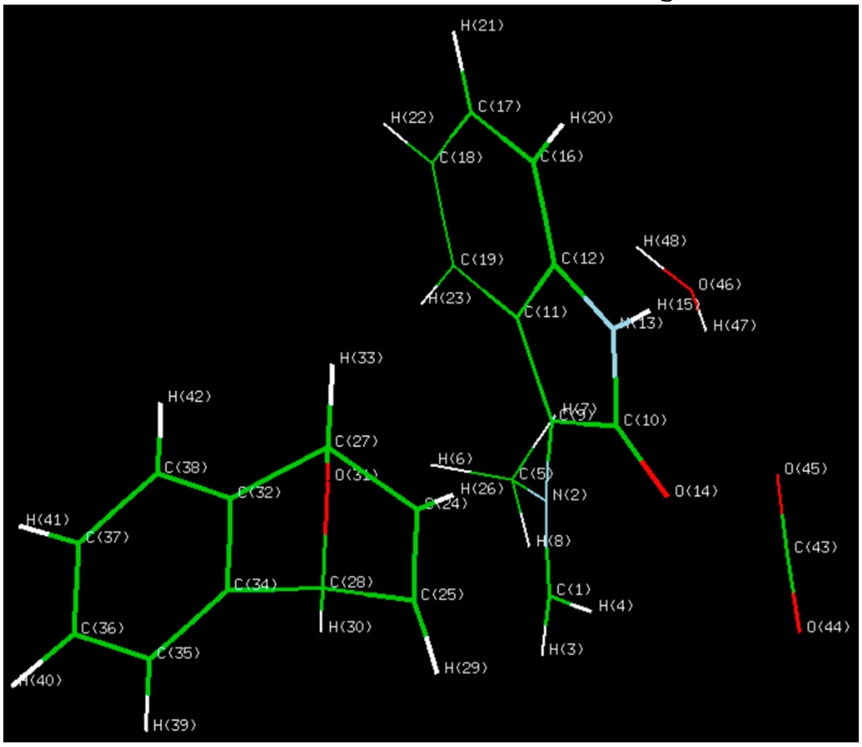

		x	y	z	q
1	C	-0.579366	-2.253562	0.518460	-0.003494
2	N	-1.060190	-1.071894	0.903223	-0.259015
3	H	-0.437964	-2.998805	1.294019	0.135823
4	H	-0.784922	-2.578286	-0.489067	0.121939
5	C	-1.117400	-0.752268	2.333086	-0.106891
6	H	-0.215211	-0.219068	2.636239	0.110782
7	H	-2.007019	-0.146452	2.513093	0.107873
8	H	-1.198790	-1.686484	2.887349	0.124113

9	C	-1.239407	-0.077393	-0.020820	0.061632
10	C	-1.671289	-0.340497	-1.384325	0.227075
11	C	-1.300485	1.353519	0.198407	-0.023598
12	C	-1.688437	1.921218	-1.042531	0.087614
13	N	-1.903999	0.897693	-1.951210	-0.424623
14	O	-1.860776	-1.418074	-1.976277	-0.426712
15	H	-2.199549	1.014345	-2.911231	0.361088
16	C	-1.797786	3.290165	-1.237627	-0.113937
17	C	-1.519709	4.127521	-0.156922	-0.116604
18	C	-1.129784	3.595377	1.074416	-0.121311
19	C	-1.006682	2.217273	1.261446	-0.100939
20	H	-2.094606	3.688869	-2.202592	0.115747
21	H	-1.605346	5.202602	-0.278580	0.107154
22	H	-0.910981	4.262121	1.902482	0.105463
23	H	-0.688050	1.837473	2.224718	0.108898
24	C	1.145206	-0.518287	-0.947047	-0.098340
25	C	1.385921	-1.667687	-0.230942	-0.105292
26	H	0.898100	-0.443153	-1.998622	0.110968
27	C	1.792265	0.607034	-0.140080	0.021091
28	C	2.179785	-1.181669	0.995811	0.025592
29	H	1.524016	-2.655778	-0.653282	0.116862
30	H	2.139073	-1.783756	1.902759	0.124720
31	O	1.585020	0.114669	1.197863	-0.262782
32	C	3.307485	0.406520	-0.257565	-0.028634
33	H	1.406925	1.619187	-0.258570	0.114880
34	C	3.559649	-0.757875	0.485094	-0.028761
35	C	4.831838	-1.277332	0.593575	-0.104638
36	C	5.874707	-0.596600	-0.060705	-0.110038
37	C	5.625016	0.555296	-0.797203	-0.109882
38	C	4.322434	1.074247	-0.909499	-0.104439
39	H	5.033185	-2.177516	1.167141	0.111334
40	H	6.889511	-0.975431	0.014330	0.107728
41	H	6.447236	1.064852	-1.290434	0.107807
42	H	4.133014	1.974752	-1.486565	0.111608
43	C	-3.586099	-2.734071	-0.386178	0.429782
44	O	-3.271714	-3.751900	-0.851128	-0.231247
45	O	-3.949524	-1.748335	0.115092	-0.211396
46	O	-4.069543	0.844342	1.602939	-0.686782
47	H	-3.858126	0.318327	0.819888	0.307367
48	H	-3.516048	1.630433	1.501812	0.314411

34

log file: geo15_ts3_m2.log
E(RM06-2X) = -1297.46827104   A.U.
Zero-point correction=   0.310959
Thermal correction to Energy=   0.331562
Thermal correction to Enthalpy=   0.332506
Thermal correction to Gibbs Free Energy=   0.262067
Sum of electronic and zero-point Energies=   -991.203362
Sum of electronic and thermal Energies=   -991.182759
Sum of electronic and thermal Enthalpies=   -991.181815
Sum of electronic and thermal Free Energies=   -991.252255

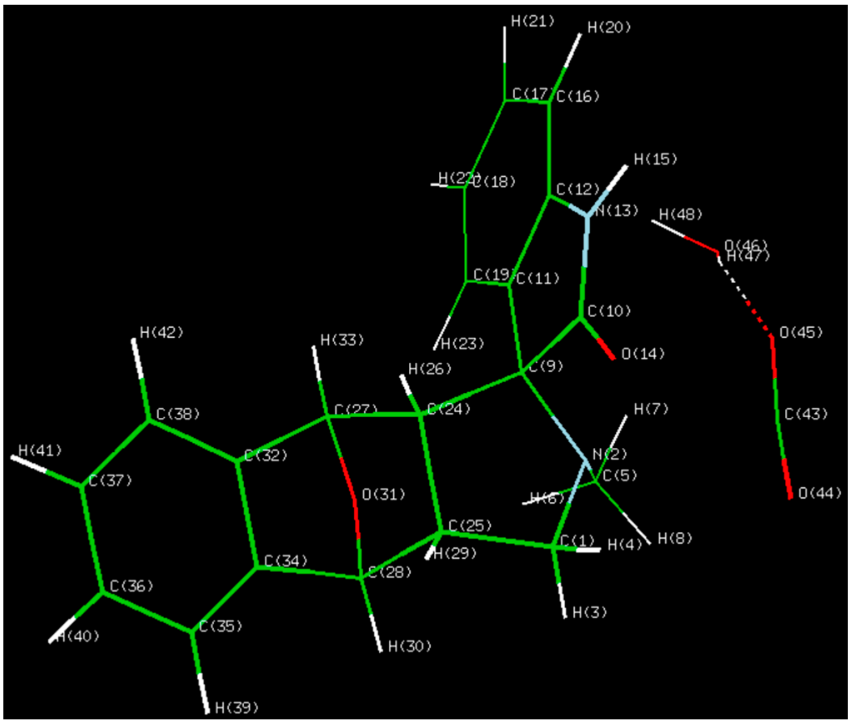

		x	y	z	q
1	C	0.268728	-2.279942	0.444360	-0.057404
2	N	-0.848084	-1.340792	0.594949	-0.351705
3	H	0.426880	-2.822519	1.381340	0.111987
4	H	0.023603	-3.013444	-0.330702	0.089791
5	C	-1.219237	-1.047684	1.974894	-0.136867
6	H	-0.427841	-0.555515	2.556782	0.081625
7	H	-2.120430	-0.430433	1.995970	0.087511
8	H	-1.463376	-1.998407	2.458461	0.101603
9	C	-0.587706	-0.219184	-0.323304	0.099606
10	C	-1.246110	-0.591839	-1.675452	0.273324
11	C	-1.269851	1.094333	-0.007805	-0.015454
12	C	-2.108317	1.434047	-1.079162	0.101491
13	N	-2.045526	0.439884	-2.061582	-0.411862
14	O	-1.081715	-1.639128	-2.282453	-0.385710
15	H	-2.621292	0.413136	-2.894996	0.368047
16	C	-2.864877	2.595774	-1.094635	-0.100500
17	C	-2.762403	3.444684	0.010484	-0.088201
18	C	-1.927748	3.133006	1.084306	-0.104324
19	C	-1.173932	1.952881	1.078731	-0.079163
20	H	-3.510420	2.832362	-1.933772	0.122585
21	H	-3.343683	4.361183	0.029935	0.116817
22	H	-1.862543	3.807354	1.931559	0.113292
23	H	-0.528319	1.713548	1.918416	0.115463
24	C	0.960696	-0.146119	-0.575610	-0.071397
25	C	1.506162	-1.484392	0.012076	-0.078360
26	H	1.175626	-0.006448	-1.637051	0.107182
27	C	1.708817	0.893025	0.292490	0.019933
28	C	2.385998	-0.951621	1.179415	0.024318
29	H	2.111006	-2.051544	-0.697056	0.103814
30	H	2.513290	-1.626365	2.025760	0.126407
31	O	1.685972	0.244815	1.576529	-0.272465
32	C	3.183707	0.818669	-0.054797	-0.026754
33	H	1.270781	1.888101	0.343043	0.118262
34	C	3.630489	-0.364807	0.543278	-0.026280
35	C	4.938434	-0.791421	0.402266	-0.100757

36	C	5.811322	0.017804	-0.338190	-0.106295
37	C	5.366295	1.196614	-0.933830	-0.107234
38	C	4.032485	1.608989	-0.808347	-0.099946
39	H	5.287190	-1.715636	0.853622	0.112977
40	H	6.850486	-0.277169	-0.448074	0.109170
41	H	6.063831	1.805763	-1.500581	0.108991
42	H	3.687364	2.523684	-1.281523	0.113271
43	C	-3.033750	-2.572986	-0.185948	0.427452
44	O	-2.551857	-3.628238	-0.107435	-0.228712
45	O	-3.623005	-1.571099	-0.288736	-0.215262
46	O	-4.307965	0.742463	1.548525	-0.694904
47	H	-4.013168	0.248552	0.772122	0.302929
48	H	-3.830370	1.580666	1.480411	0.301728

33

log file: geo14_ts1_m1.log
E(RM06-2X) = -1297.37994833 A.U.
Zero-point correction= 0.310959
Thermal correction to Energy= 0.331562
Thermal correction to Enthalpy= 0.332506
Thermal correction to Gibbs Free Energy= 0.262067
Sum of electronic and zero-point Energies= -991.203362
Sum of electronic and thermal Energies= -991.182759
Sum of electronic and thermal Enthalpies= -991.181815
Sum of electronic and thermal Free Energies= -991.252255

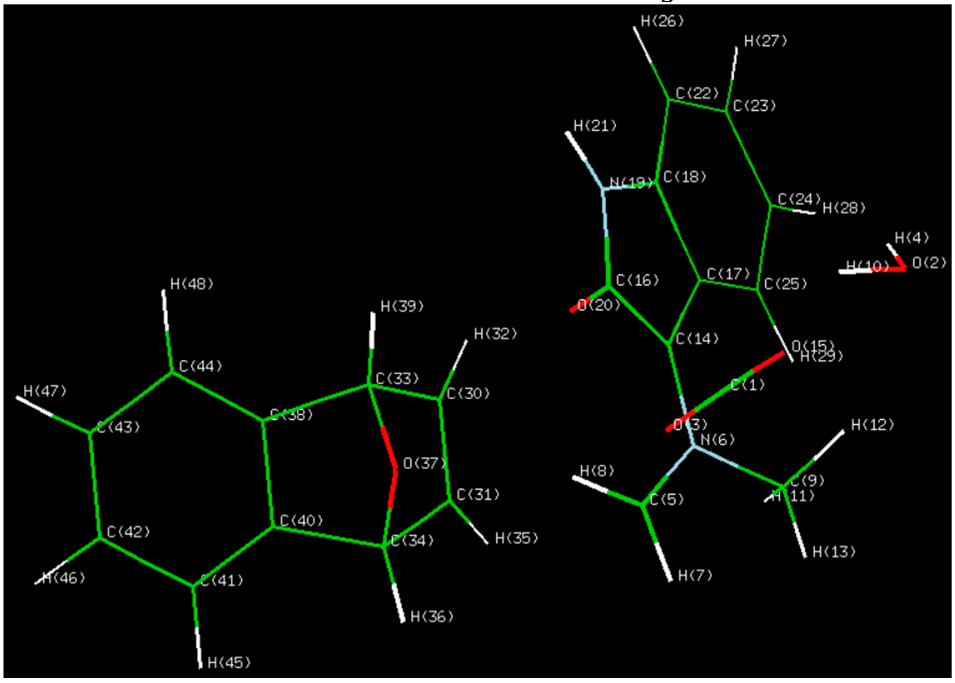

		x	y	z	q
1	C	-2.077475	-3.372345	-0.977305	0.424214
2	O	-4.871085	-0.584194	0.152733	-0.688264
3	O	-1.196580	-4.098212	-1.198253	-0.234289
4	H	-4.775921	0.361030	0.332684	0.315966
5	C	-0.533318	-1.963877	1.211084	0.016579
6	N	-1.434937	-1.029794	1.264019	-0.214945
7	H	-0.525997	-2.711541	1.994392	0.148353
8	H	0.175854	-1.973676	0.394244	0.122488
9	C	-2.343978	-0.979883	2.423231	-0.093669
10	H	-4.048444	-0.805569	-0.305601	0.301631
11	H	-2.031851	-0.165273	3.078040	0.129794

12	H	-3.357714	-0.817108	2.055464	0.115544
13	H	-2.279333	-1.924176	2.960311	0.133238
14	C	-1.617122	-0.075110	0.275572	0.023930
15	O	-3.006072	-2.709522	-0.743068	-0.215254
16	C	-1.163533	-0.276891	-1.071627	0.212870
17	C	-2.295021	1.195789	0.356542	-0.032202
18	C	-2.223602	1.746215	-0.954358	0.080911
19	N	-1.551203	0.854441	-1.769960	-0.426319
20	O	-0.585185	-1.252322	-1.596980	-0.430928
21	H	-1.383987	0.971794	-2.760279	0.359244
22	C	-2.768071	2.979501	-1.282781	-0.117664
23	C	-3.420009	3.697268	-0.280889	-0.126187
24	C	-3.509063	3.180600	1.014473	-0.126673
25	C	-2.952965	1.944647	1.345247	-0.114764
26	H	-2.689490	3.362178	-2.295786	0.113797
27	H	-3.860915	4.661631	-0.511869	0.104127
28	H	-4.019060	3.750125	1.785304	0.103090
29	H	-3.042032	1.583527	2.362818	0.106196
30	C	1.459650	0.751043	0.334272	-0.101796
31	C	1.811200	0.126354	1.458834	-0.105635
32	H	0.836317	1.625981	0.198868	0.114135
33	C	2.252734	0.054755	-0.784096	0.024320
34	C	2.827920	-0.944028	1.029423	0.027240
35	H	1.545504	0.355216	2.483090	0.119608
36	H	2.948760	-1.820993	1.662922	0.127920
37	O	2.311003	-1.290916	-0.269885	-0.275389
38	C	3.719865	0.458652	-0.585526	-0.028061
39	H	1.844403	0.082388	-1.792539	0.121599
40	C	4.095800	-0.193678	0.599044	-0.028205
41	C	5.363504	-0.059482	1.121042	-0.104122
42	C	6.275529	0.752238	0.421080	-0.109466
43	C	5.903656	1.398364	-0.751200	-0.109426
44	C	4.604121	1.260272	-1.273496	-0.103675
45	H	5.659811	-0.563419	2.036422	0.111897
46	H	7.285376	0.871280	0.801795	0.108041
47	H	6.626563	2.016023	-1.275422	0.108062
48	H	4.319545	1.766963	-2.191051	0.112136

TS14

log file: geo14_ts1.log
E(RM06-2X) = -1297.36865559 A.U.
Zero-point correction= 0.310959
Thermal correction to Energy= 0.331562
Thermal correction to Enthalpy= 0.332506
Thermal correction to Gibbs Free Energy= 0.262067
Sum of electronic and zero-point Energies= -991.203362
Sum of electronic and thermal Energies= -991.182759
Sum of electronic and thermal Enthalpies= -991.181815
Sum of electronic and thermal Free Energies= -991.252255

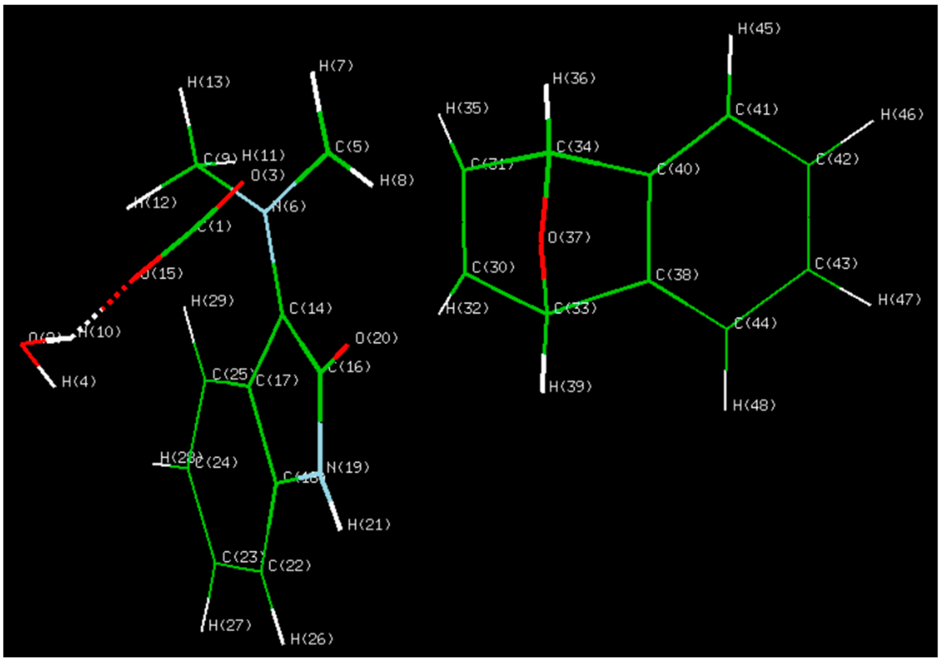

		x	y	z	q
1	C	2.473535	3.258631	-0.748748	0.429508
2	O	4.717996	0.188322	0.884657	-0.687383
3	O	1.683241	4.096924	-0.900763	-0.229292
4	H	4.401735	-0.717310	0.765894	0.313049
5	C	0.218470	1.931997	1.043718	-0.001674
6	N	1.184117	1.022904	1.158371	-0.261342
7	H	0.133256	2.660601	1.842737	0.136743
8	H	-0.133186	2.179467	0.053048	0.119538
9	C	1.759761	0.787660	2.483914	-0.099819
10	H	4.146717	0.705059	0.300391	0.307570
11	H	1.182443	0.018897	3.007195	0.119692
12	H	2.797986	0.475618	2.368705	0.110170
13	H	1.717896	1.717642	3.049186	0.127041
14	C	1.343413	0.066521	0.187890	0.058488
15	O	3.312479	2.469624	-0.578671	-0.211206
16	C	1.173485	0.356240	-1.227428	0.226926
17	C	1.923637	-1.259881	0.305590	-0.025724
18	C	1.992122	-1.769663	-1.017548	0.086292
19	N	1.532028	-0.800411	-1.893238	-0.423890
20	O	0.843594	1.407645	-1.800964	-0.422504
21	H	1.531506	-0.873024	-2.901942	0.361182
22	C	2.451064	-3.045665	-1.308477	-0.114445
23	C	2.868944	-3.848114	-0.246953	-0.117464
24	C	2.808470	-3.375536	1.065545	-0.122715
25	C	2.330713	-2.095949	1.354487	-0.105190
26	H	2.486330	-3.397737	-2.334658	0.115859
27	H	3.242377	-4.847493	-0.446104	0.107127
28	H	3.133642	-4.012753	1.881828	0.105243
29	H	2.292720	-1.769011	2.386347	0.109066
30	C	-1.181699	-0.453927	0.407440	-0.099151
31	C	-1.524196	0.593483	1.226870	-0.106591
32	H	-0.785193	-1.418201	0.696796	0.105823
33	C	-1.978232	-0.227722	-0.881132	0.023177
34	C	-2.580120	1.374187	0.423153	0.026332
35	H	-1.504219	0.578938	2.310793	0.112785
36	H	-2.730536	2.425961	0.664317	0.126588
37	O	-2.062600	1.209559	-0.906905	-0.278214

38	C	-3.433027	-0.562929	-0.525251	-0.027962
39	H	-1.579116	-0.622497	-1.814914	0.122312
40	C	-3.823199	0.481788	0.328156	-0.028047
41	C	-5.084012	0.519891	0.882663	-0.104353
42	C	-5.971587	-0.524569	0.563445	-0.109534
43	C	-5.583879	-1.560168	-0.277723	-0.109655
44	C	-4.292689	-1.594280	-0.835982	-0.103848
45	H	-5.393087	1.326639	1.540964	0.111607
46	H	-6.974830	-0.518081	0.978668	0.107939
47	H	-6.287683	-2.353112	-0.511819	0.107971
48	H	-3.995300	-2.405609	-1.493971	0.111977

35

log file: geo14_ts1_m2.log

E(RM06-2X) = -1297.46675307 A.U.

Zero-point correction= 0.310959

Thermal correction to Energy= 0.331562

Thermal correction to Enthalpy= 0.332506

Thermal correction to Gibbs Free Energy= 0.262067

Sum of electronic and zero-point Energies= -991.203362

Sum of electronic and thermal Energies= -991.182759

Sum of electronic and thermal Enthalpies= -991.181815

Sum of electronic and thermal Free Energies= -991.252255

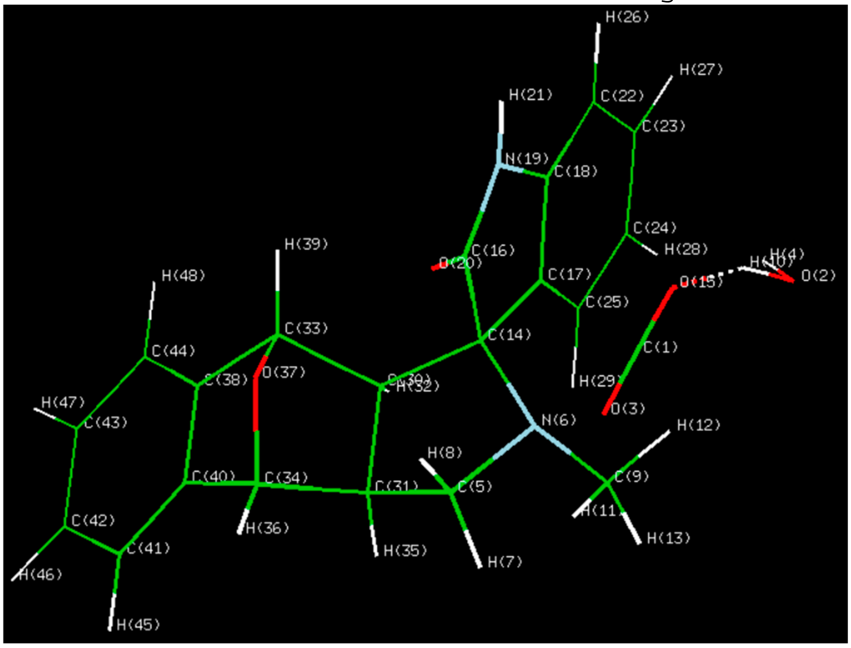

		x	y	z	q
1	C	-2.912716	-2.621132	-0.204109	0.424316
2	O	-4.515676	0.639660	1.330206	-0.694505
3	O	-2.382067	-3.644677	-0.054469	-0.232112
4	H	-4.073532	1.498934	1.297073	0.303081
5	C	0.249846	-2.093471	0.925769	-0.060605
6	N	-0.961678	-1.257310	0.905956	-0.349515
7	H	0.213282	-2.775792	1.778069	0.111535
8	H	0.280666	-2.685685	0.006212	0.094146
9	C	-1.401174	-0.890675	2.250588	-0.131670
10	H	-4.096819	0.140331	0.616590	0.302913
11	H	-0.658553	-0.311934	2.822736	0.090745
12	H	-2.330684	-0.319752	2.194628	0.089513
13	H	-1.607059	-1.814692	2.797691	0.104937
14	C	-0.610274	-0.118995	0.026143	0.098677

15	O	-3.542441	-1.657501	-0.395082	-0.218097
16	C	-1.019522	-0.553238	-1.410932	0.275289
17	C	-1.401629	1.164734	0.181340	-0.017331
18	C	-2.046434	1.454153	-1.027057	0.100246
19	N	-1.772606	0.445631	-1.955279	-0.413088
20	O	-0.790825	-1.623813	-1.949050	-0.375118
21	H	-2.224784	0.352772	-2.857288	0.367539
22	C	-2.811525	2.596657	-1.208814	-0.101324
23	C	-2.909947	3.482083	-0.133093	-0.089744
24	C	-2.245054	3.233034	1.068413	-0.105469
25	C	-1.474533	2.074887	1.226051	-0.082429
26	H	-3.307493	2.793976	-2.153282	0.122221
27	H	-3.504992	4.383415	-0.241510	0.116271
28	H	-2.322603	3.941929	1.885942	0.112737
29	H	-0.951415	1.892966	2.159925	0.117932
30	C	0.922942	0.129701	0.224846	-0.073130
31	C	1.452393	-1.136546	0.957114	-0.081847
32	H	1.054608	1.044347	0.804798	0.102901
33	C	1.827115	0.147909	-1.037593	0.022201
34	C	2.614246	-1.559689	0.025887	0.023389
35	H	1.790904	-0.927281	1.973614	0.101943
36	H	2.912339	-2.606951	0.078687	0.127148
37	O	2.060534	-1.252023	-1.265995	-0.284687
38	C	3.199031	0.596140	-0.562304	-0.025869
39	H	1.418263	0.628444	-1.927484	0.125286
40	C	3.705093	-0.509040	0.134155	-0.026359
41	C	4.928967	-0.452665	0.775012	-0.101072
42	C	5.659519	0.740896	0.685072	-0.107075
43	C	5.155648	1.839412	-0.007782	-0.107193
44	C	3.902257	1.784147	-0.634135	-0.100686
45	H	5.322072	-1.303069	1.324516	0.112886
46	H	6.633388	0.809029	1.160252	0.108962
47	H	5.742657	2.751005	-0.064872	0.108975
48	H	3.508039	2.646781	-1.163321	0.113153

36

log file: nn1.log
E(RM06-2X) = -272.616131651 A.U.
Zero-point correction= 0.310959
Thermal correction to Energy= 0.331562
Thermal correction to Enthalpy= 0.332506
Thermal correction to Gibbs Free Energy= 0.262067
Sum of electronic and zero-point Energies= -991.203362
Sum of electronic and thermal Energies= -991.182759
Sum of electronic and thermal Enthalpies= -991.181815
Sum of electronic and thermal Free Energies= -991.252255

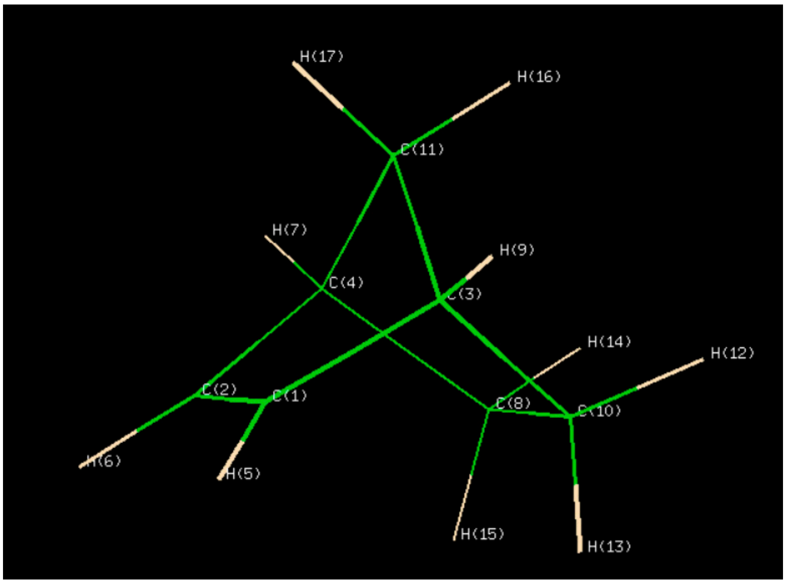

		x	y	z	q
1	C	1.276042	0.668758	-0.498352	-0.118472
2	C	1.275870	-0.668920	-0.498528	-0.118472
3	C	0.085078	1.125250	0.325809	-0.088149
4	C	0.084839	-1.125319	0.325557	-0.088150
5	H	1.916614	1.326288	-1.077256	0.102924
6	H	1.916416	-1.326454	-1.077453	0.102924
7	H	0.112987	-2.152670	0.691233	0.091858
8	C	-1.178368	-0.776795	-0.523378	-0.160372
9	H	0.113461	2.152531	0.691657	0.091860
10	C	-1.178108	0.777142	-0.523361	-0.160374
11	C	0.026544	-0.000157	1.376478	-0.158262
12	H	-2.077838	1.171632	-0.041646	0.084374
13	H	-1.120553	1.203186	-1.528090	0.081823
14	H	-2.078050	-1.170933	-0.041272	0.084372
15	H	-1.121396	-1.202918	-1.528107	0.081831
16	H	-0.902292	-0.000087	1.957265	0.087075
17	H	0.889270	-0.000327	2.048321	0.083222

38

log file: geo23_ts1_m1.log
E(RM06-2X) = -1109.12714236 A.U.

Zero-point correction=	0.310959
Thermal correction to Energy=	0.331562
Thermal correction to Enthalpy=	0.332506
Thermal correction to Gibbs Free Energy=	0.262067
Sum of electronic and zero-point Energies=	-991.203362
Sum of electronic and thermal Energies=	-991.182759
Sum of electronic and thermal Enthalpies=	-991.181815
Sum of electronic and thermal Free Energies=	-991.252255

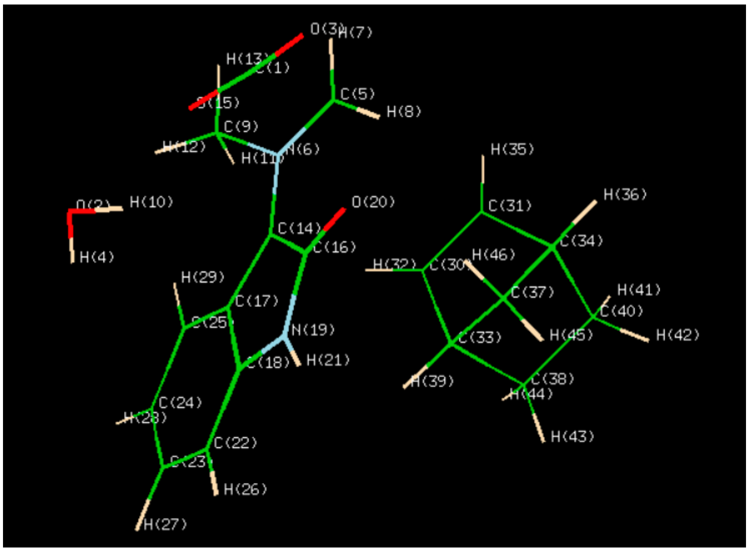

		x	y	z	q
1	C	2.816880	2.851354	-0.607204	0.424722
2	O	3.891606	-0.918584	0.638980	-0.687933
3	O	2.383092	3.901425	-0.854024	-0.233900
4	H	3.389617	-1.744329	0.672460	0.317618
5	C	0.469646	2.201919	1.170733	0.020495
6	N	0.847009	0.962633	1.251874	-0.213396
7	H	0.640584	2.847650	2.022377	0.150151
8	H	0.012590	2.556958	0.257858	0.134494
9	C	1.404580	0.471761	2.523984	-0.092416
10	H	3.326637	-0.338011	0.110115	0.300649
11	H	0.651472	-0.140703	3.022203	0.129727
12	H	2.296717	-0.114447	2.301543	0.116111
13	H	1.652618	1.326717	3.149947	0.134066
14	C	0.769035	0.057507	0.204515	0.027533
15	O	3.315422	1.834641	-0.335507	-0.214485
16	C	0.685764	0.485192	-1.161935	0.213004
17	C	0.810833	-1.383856	0.242663	-0.028055
18	C	0.741392	-1.799086	-1.117040	0.083428
19	N	0.673080	-0.673294	-1.918279	-0.424850
20	O	0.666368	1.637297	-1.649264	-0.435161
21	H	0.629381	-0.669561	-2.928323	0.360101
22	C	0.742302	-3.133944	-1.495901	-0.116299
23	C	0.824493	-4.100519	-0.494111	-0.125683
24	C	0.895225	-3.722260	0.849151	-0.125855
25	C	0.884643	-2.379687	1.229126	-0.112666
26	H	0.685245	-3.407540	-2.545052	0.114119
27	H	0.832964	-5.151936	-0.762660	0.104297
28	H	0.956748	-4.485496	1.618786	0.103241
29	H	0.936400	-2.130636	2.282420	0.106489
30	C	-2.245678	-0.009885	1.046990	-0.112968
31	C	-2.779299	1.200764	1.252937	-0.118907
32	H	-1.689496	-0.616463	1.756140	0.097347
33	C	-2.711180	-0.491445	-0.315108	-0.089979
34	C	-3.617692	1.539709	0.031374	-0.088422
35	H	-2.749377	1.785584	2.167029	0.100867
36	H	-3.862859	2.593126	-0.111781	0.091241
37	C	-2.794739	0.848422	-1.072222	-0.161388
38	C	-4.230886	-0.811655	-0.153551	-0.160669
39	H	-2.122163	-1.291687	-0.769306	0.082953
40	C	-4.856369	0.591088	0.088155	-0.160925

41	H	-5.380350	0.664717	1.044522	0.081108
42	H	-5.565826	0.847607	-0.704448	0.083932
43	H	-4.622126	-1.264913	-1.069362	0.084095
44	H	-4.413368	-1.507113	0.669619	0.081485
45	H	-3.332733	0.769759	-2.023624	0.086119
46	H	-1.818904	1.314838	-1.232443	0.074549

TS15

log file: geo23_ts1.log
E(RM06-2X) = -1109.11286197 A.U.
Zero-point correction= 0.310959
Thermal correction to Energy= 0.331562
Thermal correction to Enthalpy= 0.332506
Thermal correction to Gibbs Free Energy= 0.262067
Sum of electronic and zero-point Energies= -991.203362
Sum of electronic and thermal Energies= -991.182759
Sum of electronic and thermal Enthalpies= -991.181815
Sum of electronic and thermal Free Energies= -991.252255

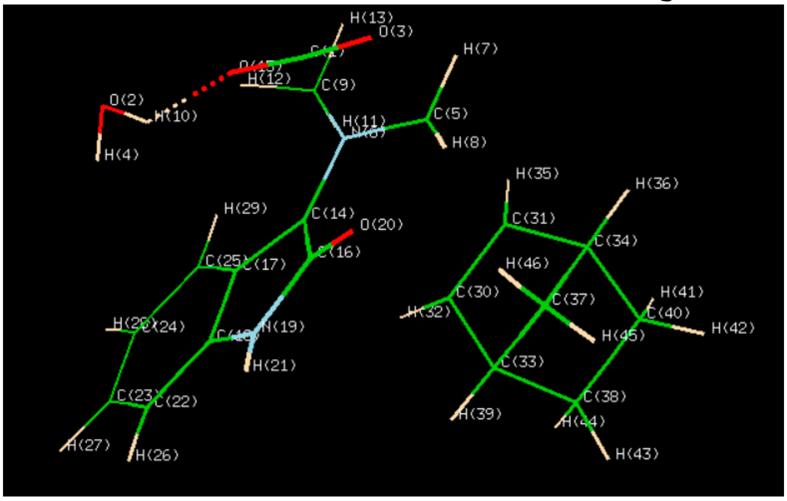

		x	y	z	q
1	C	0.727555	3.575659	-0.616073	0.428929
2	O	3.741816	1.296991	0.940989	-0.688561
3	O	-0.290926	4.130875	-0.692446	-0.230078
4	H	3.771804	0.331237	0.906503	0.313457
5	C	-1.072136	1.532188	1.047339	0.003381
6	N	0.145386	0.991388	1.157029	-0.269845
7	H	-1.397487	2.159954	1.870337	0.135604
8	H	-1.429900	1.759871	0.055024	0.121421
9	C	0.726628	0.868263	2.493238	-0.101418
10	H	3.036579	1.521405	0.318603	0.305111
11	H	0.416224	-0.076094	2.952935	0.117210
12	H	1.813043	0.911540	2.410909	0.109324
13	H	0.374654	1.698863	3.104319	0.125228
14	C	0.617277	0.175731	0.153084	0.050153
15	O	1.777869	3.083470	-0.518465	-0.212397
16	C	0.363558	0.447187	-1.245255	0.220232
17	C	1.564802	-0.917909	0.232916	-0.027200
18	C	1.789452	-1.333510	-1.107767	0.083097
19	N	1.058257	-0.517014	-1.952423	-0.425831
20	O	-0.275309	1.372860	-1.785789	-0.423636
21	H	1.075704	-0.549811	-2.962790	0.359815
22	C	2.616729	-2.397147	-1.436465	-0.116186
23	C	3.259280	-3.072682	-0.399028	-0.122058
24	C	3.055622	-2.689122	0.928474	-0.124739

25	C	2.209096	-1.628663	1.256679	-0.108692
26	H	2.759134	-2.683270	-2.473995	0.114646
27	H	3.920426	-3.902205	-0.628361	0.105462
28	H	3.559230	-3.226473	1.725983	0.104000
29	H	2.070749	-1.368760	2.299353	0.107522
30	C	-1.671123	-1.154342	0.424291	-0.087263
31	C	-2.302532	-0.194227	1.192267	-0.101655
32	H	-0.941231	-1.870674	0.786817	0.095509
33	C	-2.537181	-1.409834	-0.788667	-0.088110
34	C	-3.609966	0.122739	0.470661	-0.085607
35	H	-2.233219	-0.173928	2.277961	0.103211
36	H	-4.095547	1.059146	0.753388	0.092647
37	C	-3.184236	-0.029859	-0.999019	-0.163452
38	C	-3.762523	-2.204859	-0.232204	-0.157712
39	H	-2.048612	-1.874935	-1.646897	0.091828
40	C	-4.498058	-1.145650	0.637274	-0.159847
41	H	-4.586680	-1.443816	1.685480	0.082757
42	H	-5.505978	-0.956249	0.255849	0.085095
43	H	-4.392888	-2.543420	-1.060588	0.086174
44	H	-3.453683	-3.085385	0.337408	0.083263
45	H	-4.040949	-0.070942	-1.679491	0.089090
46	H	-2.477938	0.725989	-1.345755	0.080112

39

log file: geo23_ts1_m2.log
E(RM06-2X) = -1109.20324726 A.U.
Zero-point correction= 0.310959
Thermal correction to Energy= 0.331562
Thermal correction to Enthalpy= 0.332506
Thermal correction to Gibbs Free Energy= 0.262067
Sum of electronic and zero-point Energies= -991.203362
Sum of electronic and thermal Energies= -991.182759
Sum of electronic and thermal Enthalpies= -991.181815
Sum of electronic and thermal Free Energies= -991.252255

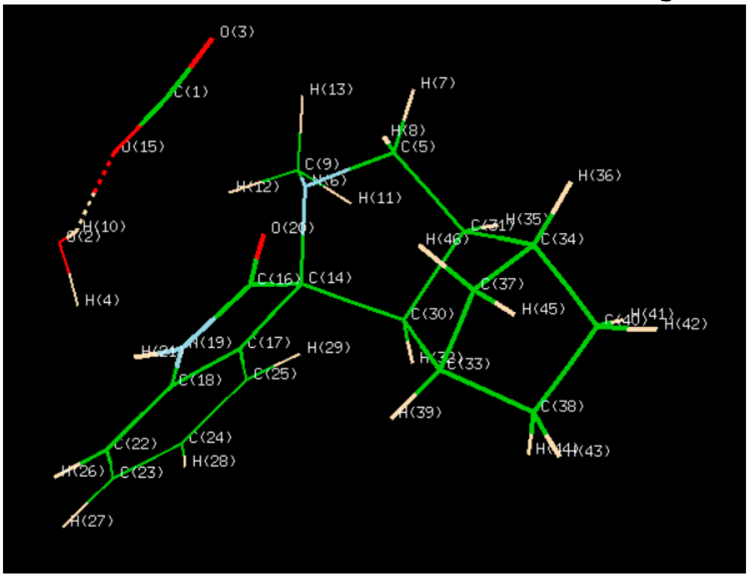

		x	y	z	q
1	C	-1.279356	3.052261	0.302854	0.423681
2	O	-3.978397	0.517902	-1.126072	-0.695649
3	O	-0.456797	3.859288	0.147687	-0.233880
4	H	-3.844030	-0.438145	-1.069433	0.301185
5	C	1.498361	1.550173	-1.003755	-0.061988

6	N	0.077751	1.171050	-0.901057	-0.350515
7	H	1.637484	2.220356	-1.855813	0.107965
8	H	1.779690	2.094349	-0.096575	0.093357
9	C	-0.547965	1.011870	-2.211778	-0.132272
10	H	-3.402306	0.872008	-0.435788	0.303408
11	H	-0.084021	0.235747	-2.840858	0.089958
12	H	-1.609583	0.782979	-2.092795	0.088391
13	H	-0.468558	1.967198	-2.738125	0.103622
14	C	0.074047	-0.038996	-0.045124	0.097218
15	O	-2.178699	2.336970	0.506739	-0.218005
16	C	-0.102411	0.473022	1.411782	0.277865
17	C	-1.107635	-0.982907	-0.164091	-0.016893
18	C	-1.749681	-1.074347	1.076496	0.099231
19	N	-1.109612	-0.236991	1.994636	-0.413384
20	O	0.483450	1.404988	1.942414	-0.366660
21	H	-1.462085	-0.019301	2.919396	0.367009
22	C	-2.841653	-1.901072	1.294157	-0.101664
23	C	-3.286146	-2.674527	0.219176	-0.090532
24	C	-2.639328	-2.628326	-1.016580	-0.105917
25	C	-1.535529	-1.789062	-1.209188	-0.083647
26	H	-3.325316	-1.946112	2.264158	0.121906
27	H	-4.141771	-3.328615	0.354381	0.115951
28	H	-2.991933	-3.247972	-1.834317	0.112431
29	H	-1.031615	-1.767018	-2.170622	0.117059
30	C	1.425873	-0.782200	-0.333707	-0.073299
31	C	2.298344	0.242148	-1.115670	-0.083395
32	H	1.181941	-1.655950	-0.944096	0.094365
33	C	2.337125	-1.204617	0.839933	-0.088430
34	C	3.644619	0.191313	-0.371222	-0.088266
35	H	2.411929	-0.044331	-2.166112	0.091282
36	H	4.307059	1.031526	-0.595925	0.091291
37	C	3.198855	0.048520	1.093523	-0.169365
38	C	3.396720	-2.151792	0.238836	-0.159035
39	H	1.801888	-1.617973	1.699889	0.090902
40	C	4.270279	-1.196225	-0.631275	-0.160141
41	H	4.249291	-1.459345	-1.693375	0.083137
42	H	5.314377	-1.216761	-0.305664	0.084332
43	H	3.993113	-2.605680	1.035862	0.085193
44	H	2.945238	-2.961189	-0.342275	0.084207
45	H	4.037821	-0.161878	1.765710	0.087208
46	H	2.640852	0.903095	1.474974	0.080774

37

log file: on2.log
E(RM06-2X) = -308.507465825 A.U.
Zero-point correction= 0.310959
Thermal correction to Energy= 0.331562
Thermal correction to Enthalpy= 0.332506
Thermal correction to Gibbs Free Energy= 0.262067
Sum of electronic and zero-point Energies= -991.203362
Sum of electronic and thermal Energies= -991.182759
Sum of electronic and thermal Enthalpies= -991.181815
Sum of electronic and thermal Free Energies= -991.252255

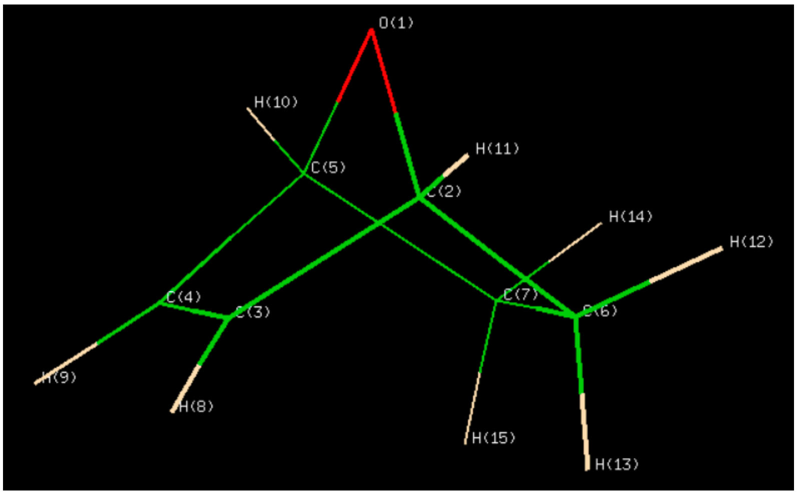

		x	y	z	q
1	O	0.009000	-0.000008	1.320918	-0.305137
2	C	0.078481	1.068738	0.365693	0.012668
3	C	1.287271	0.666754	-0.464651	-0.114770
4	C	1.287266	-0.666756	-0.464659	-0.114770
5	C	0.078475	-1.068743	0.365680	0.012667
6	C	-1.179172	0.775311	-0.503380	-0.161189
7	C	-1.179180	-0.775298	-0.503380	-0.161188
8	H	1.918460	1.347657	-1.022663	0.115150
9	H	1.918454	-1.347655	-1.022678	0.115150
10	H	0.101701	-2.036586	0.864994	0.117640
11	H	0.101714	2.036574	0.865019	0.117640
12	H	-2.071212	1.170613	-0.012294	0.092225
13	H	-1.104354	1.215098	-1.499637	0.090843
14	H	-2.071216	-1.170585	-0.012270	0.092225
15	H	-1.104391	-1.215088	-1.499637	0.090843

40

log file: geo26_ts1_m1.log
E(RM06-2X) = -1145.02222772 A.U.

Zero-point correction=	0.310959
Thermal correction to Energy=	0.331562
Thermal correction to Enthalpy=	0.332506
Thermal correction to Gibbs Free Energy=	0.262067
Sum of electronic and zero-point Energies=	-991.203362
Sum of electronic and thermal Energies=	-991.182759
Sum of electronic and thermal Enthalpies=	-991.181815
Sum of electronic and thermal Free Energies=	-991.252255

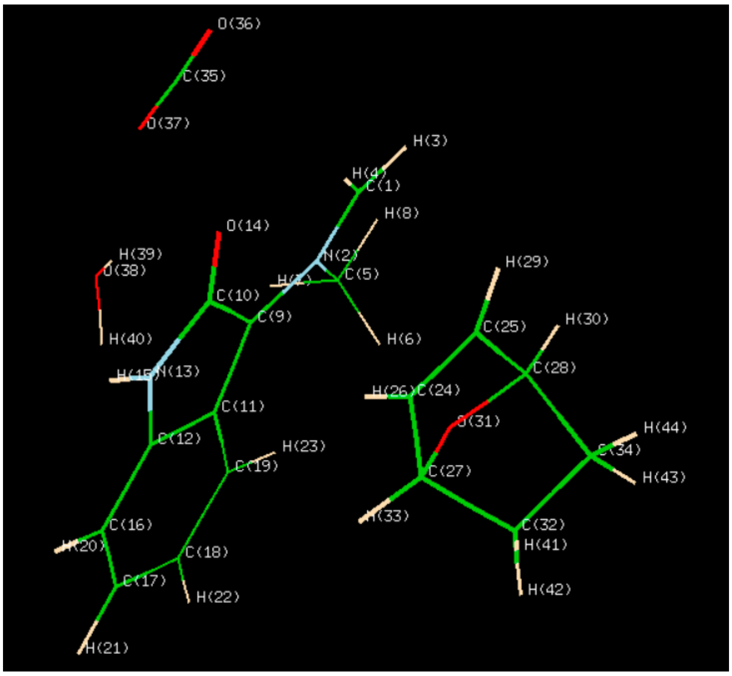

		x	y	z	q
1	C	-0.661422	-2.226303	0.784326	0.011078
2	N	-0.579138	-0.952431	1.031959	-0.213567
3	H	-0.630320	-2.912174	1.620902	0.146118
4	H	-0.776349	-2.559279	-0.236337	0.129034
5	C	-0.380369	-0.517478	2.424333	-0.098835
6	H	0.638702	-0.143046	2.517992	0.118783
7	H	-1.111687	0.260949	2.644811	0.114750
8	H	-0.526316	-1.372415	3.081468	0.130880
9	C	-0.661378	0.038296	0.070092	0.031913
10	C	-1.119337	-0.225994	-1.265257	0.211900
11	C	-0.314991	1.435616	0.174664	-0.025996
12	C	-0.581398	1.989118	-1.109509	0.081524
13	N	-1.064005	0.986206	-1.930236	-0.427444
14	O	-1.531974	-1.284401	-1.787297	-0.437748
15	H	-1.338011	1.095087	-2.897259	0.358967
16	C	-0.355712	3.322894	-1.417501	-0.117866
17	C	0.150892	4.151566	-0.416991	-0.126333
18	C	0.423752	3.637206	0.852925	-0.126608
19	C	0.200897	2.294112	1.159078	-0.111771
20	H	-0.570644	3.700537	-2.412544	0.113451
21	H	0.334260	5.199903	-0.629371	0.103954
22	H	0.821218	4.291488	1.622758	0.102835
23	H	0.433967	1.939527	2.155503	0.106044
24	C	1.964681	-1.030990	-1.279496	-0.107517
25	C	2.057952	-2.146872	-0.551220	-0.110781
26	H	1.599425	-0.908744	-2.292564	0.112508
27	C	2.661614	0.043390	-0.459825	0.012095
28	C	2.800070	-1.740035	0.712719	0.013750
29	H	1.797031	-3.162386	-0.823913	0.114517
30	H	2.616976	-2.318420	1.618437	0.115520
31	O	2.377156	-0.378612	0.881999	-0.276192
32	C	4.188770	-0.247051	-0.542081	-0.161736
33	H	2.359452	1.079252	-0.614164	0.106222
34	C	4.290058	-1.538191	0.312404	-0.161770
35	C	-3.528820	-2.084304	-0.227262	0.425372
36	O	-3.510020	-3.145991	-0.700770	-0.234603

37	O	-3.611184	-1.043273	0.287898	-0.214934
38	O	-3.044046	1.490469	1.770054	-0.689053
39	H	-2.884477	0.922735	1.003114	0.300057
40	H	-2.380221	2.186285	1.670422	0.317765
41	H	4.529374	-0.373670	-1.571515	0.090375
42	H	4.749373	0.572439	-0.086631	0.091536
43	H	4.898487	-1.374049	1.204711	0.091452
44	H	4.690750	-2.395859	-0.231352	0.090382

TS16

log file: geo26_ts1.log
E(RM06-2X) = -1145.00996068 A.U.

Zero-point correction=	0.310959
Thermal correction to Energy=	0.331562
Thermal correction to Enthalpy=	0.332506
Thermal correction to Gibbs Free Energy=	0.262067
Sum of electronic and zero-point Energies=	-991.203362
Sum of electronic and thermal Energies=	-991.182759
Sum of electronic and thermal Enthalpies=	-991.181815
Sum of electronic and thermal Free Energies=	-991.252255

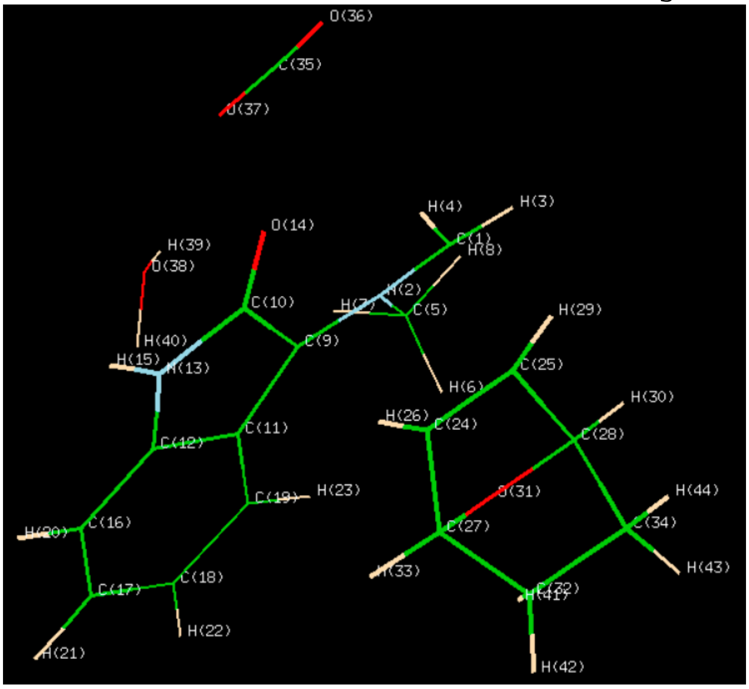

		x	y	z	q
1	C	0.102296	-2.236774	0.528272	-0.003809
2	N	-0.307451	-1.026765	0.917838	-0.263597
3	H	0.276150	-2.965884	1.312820	0.134426
4	H	-0.221996	-2.586333	-0.439182	0.120366
5	C	-0.213703	-0.657657	2.332670	-0.107693
6	H	0.732259	-0.150256	2.528416	0.108972
7	H	-1.058544	-0.010857	2.575387	0.107259
8	H	-0.274148	-1.567865	2.928543	0.123106
9	C	-0.515543	-0.051733	-0.022938	0.060192
10	C	-1.063852	-0.334624	-1.338261	0.224600
11	C	-0.487023	1.386808	0.148932	-0.023920
12	C	-0.941330	1.934625	-1.078861	0.086661
13	N	-1.276387	0.895790	-1.931247	-0.425762
14	O	-1.355309	-1.418833	-1.876834	-0.430720
15	H	-1.643404	0.996687	-2.867992	0.360376
16	C	-0.998444	3.300565	-1.314198	-0.114908

17	C	-0.597624	4.157210	-0.288889	-0.118080
18	C	-0.140059	3.645130	0.927712	-0.122572
19	C	-0.070063	2.269372	1.154236	-0.102413
20	H	-1.349150	3.682341	-2.267937	0.115169
21	H	-0.639940	5.230827	-0.442234	0.106556
22	H	0.174304	4.325792	1.712613	0.104817
23	H	0.302578	1.906120	2.104425	0.108168
24	C	1.762172	-0.649511	-1.116345	-0.096767
25	C	1.990105	-1.791471	-0.375913	-0.105534
26	H	1.440849	-0.601731	-2.150270	0.108250
27	C	2.541772	0.443262	-0.419096	0.009709
28	C	2.916927	-1.320960	0.741869	0.014334
29	H	2.047557	-2.793106	-0.788338	0.114512
30	H	2.899803	-1.880099	1.677971	0.115256
31	O	2.461562	0.023882	0.951735	-0.274861
32	C	4.046187	0.197870	-0.723371	-0.161160
33	H	2.194756	1.471165	-0.528704	0.106428
34	C	4.314885	-1.078542	0.116250	-0.161888
35	C	-2.995283	-2.588611	-0.093618	0.429761
36	O	-2.744300	-3.642041	-0.516067	-0.231110
37	O	-3.295418	-1.564115	0.370355	-0.211257
38	O	-3.155587	1.042755	1.788425	-0.688553
39	H	-2.975138	0.474808	1.027075	0.304559
40	H	-2.592507	1.812760	1.632561	0.315437
41	H	4.233577	0.067883	-1.791270	0.091200
42	H	4.641607	1.041372	-0.364783	0.091603
43	H	5.051427	-0.888282	0.900530	0.091512
44	H	4.650729	-1.931449	-0.477389	0.091362

41

log file: geo26_ts1_m2.log
E(RM06-2X) = -1145.10198491 A.U.
Zero-point correction= 0.310959
Thermal correction to Energy= 0.331562
Thermal correction to Enthalpy= 0.332506
Thermal correction to Gibbs Free Energy= 0.262067
Sum of electronic and zero-point Energies= -991.203362
Sum of electronic and thermal Energies= -991.182759
Sum of electronic and thermal Enthalpies= -991.181815
Sum of electronic and thermal Free Energies= -991.252255

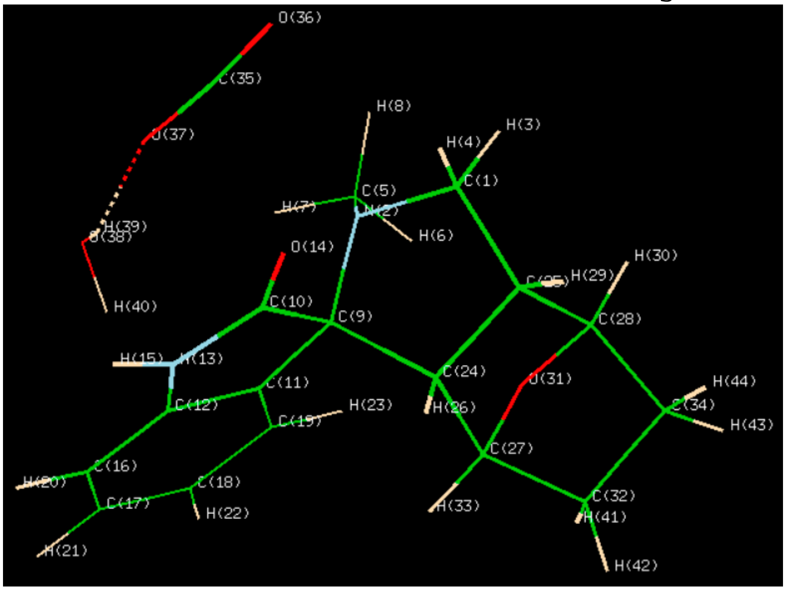

		x	y	z	q
1	C	1.415451	-1.951107	0.445402	-0.058795
2	N	0.163195	-1.212779	0.645887	-0.351408
3	H	1.756208	-2.369296	1.397338	0.110367
4	H	1.235929	-2.784702	-0.241616	0.088696
5	C	-0.090962	-0.813789	2.026296	-0.136981
6	H	0.652651	-0.116451	2.436012	0.082030
7	H	-1.085531	-0.368984	2.109087	0.088043
8	H	-0.087894	-1.721719	2.636914	0.101277
9	C	0.111094	-0.178977	-0.404730	0.098851
10	C	-0.624899	-0.814480	-1.609887	0.272730
11	C	-0.754341	1.031076	-0.128357	-0.015186
12	C	-1.761843	1.096986	-1.101235	0.100996
13	N	-1.638730	0.015434	-1.980556	-0.412499
14	O	-0.350610	-1.885727	-2.129789	-0.387492
15	H	-2.294989	-0.207943	-2.719512	0.367494
16	C	-2.709488	2.109146	-1.126275	-0.101615
17	C	-2.628159	3.091370	-0.136380	-0.089476
18	C	-1.627214	3.054383	0.834987	-0.105145
19	C	-0.681130	2.021950	0.841366	-0.079664
20	H	-3.482834	2.133061	-1.886796	0.121991
21	H	-3.356867	3.895892	-0.127199	0.116208
22	H	-1.579839	3.829979	1.592097	0.112685
23	H	0.097269	1.998357	1.597630	0.113486
24	C	1.585132	0.122081	-0.863681	-0.073968
25	C	2.433499	-0.991202	-0.177909	-0.080984
26	H	1.656257	0.122116	-1.953913	0.105709
27	C	2.214124	1.380316	-0.255890	0.009702
28	C	3.265156	-0.153189	0.818869	0.013133
29	H	3.088510	-1.517337	-0.875753	0.103589
30	H	3.527059	-0.663744	1.747198	0.116252
31	O	2.418652	0.975866	1.106134	-0.282729
32	C	3.651332	1.549267	-0.790241	-0.158703
33	H	1.603681	2.281699	-0.293658	0.109776
34	C	4.424080	0.508310	0.061947	-0.161010
35	C	-1.826992	-2.877113	0.286512	0.426451
36	O	-1.156521	-3.821555	0.389770	-0.230085
37	O	-2.598986	-2.008660	0.179185	-0.215871
38	O	-3.481063	0.365325	1.837563	-0.696248
39	H	-3.194661	-0.167615	1.084081	0.302722
40	H	-3.044219	1.216255	1.694453	0.300335
41	H	3.708852	1.353862	-1.863672	0.094950
42	H	4.010262	2.564083	-0.607256	0.093824
43	H	5.102944	0.998197	0.763747	0.092202
44	H	4.995136	-0.206462	-0.534174	0.094342
